# Fluorescent Probes for Disease Diagnosis

**DOI:** 10.1021/acs.chemrev.3c00776

**Published:** 2024-05-17

**Authors:** Xin Wang, Qi Ding, Robin R. Groleau, Luling Wu, Yuantao Mao, Feida Che, Oxana Kotova, Eoin M. Scanlan, Simon E. Lewis, Ping Li, Bo Tang, Tony D. James, Thorfinnur Gunnlaugsson

**Affiliations:** † College of Chemistry, Chemical Engineering and Materials Science, Key Laboratory of Molecular and Nano Probes, Ministry of Education, Collaborative Innovation Center of Functionalized Probes for Chemical Imaging in Universities of Shandong, Institutes of Biomedical Sciences, Shandong Normal University, Jinan 250014, People’s Republic of China; ‡ Department of Chemistry, University of Bath, Bath BA2 7AY, U.K.; § School of Chemistry and Trinity Biomedical Sciences Institute (TBSI), Trinity College Dublin, The University of Dublin, Dublin 2 D02 R590, Ireland; ∥ Advanced Materials and BioEngineering Research (AMBER) Centre, Trinity College Dublin, The University of Dublin, Dublin 2 D02 W9K7, Ireland; ⊥ Synthesis and Solid-State Pharmaceutical Centre (SSPC), School of Chemistry, Trinity College Dublin, The University of Dublin, Dublin 2 , Ireland; # Laoshan Laboratory, 168 Wenhai Middle Road, Aoshanwei Jimo, Qingdao 266237, Shandong, People’s Republic of China; ∇ School of Chemistry and Chemical Engineering, Henan Normal University, Xinxiang 453007, People’s Republic of China

## Abstract

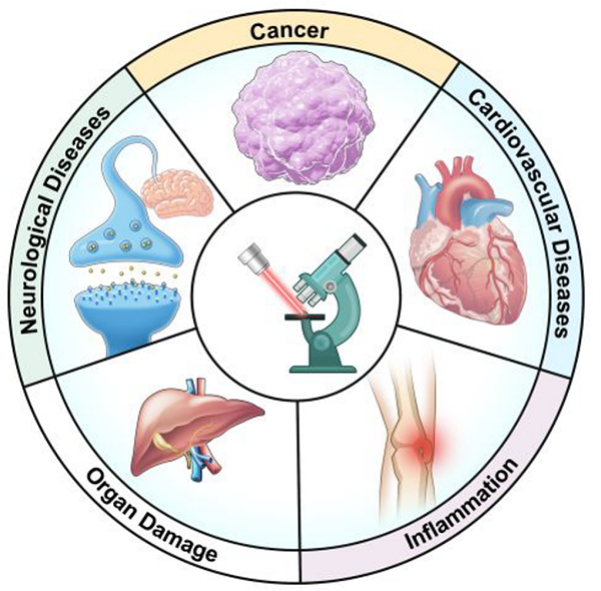

The identification and detection of disease-related biomarkers is essential for early clinical diagnosis, evaluating disease progression, and for the development of therapeutics. Possessing the advantages of high sensitivity and selectivity, fluorescent probes have become effective tools for monitoring disease-related active molecules at the cellular level and *in vivo*. In this review, we describe current fluorescent probes designed for the detection and quantification of key bioactive molecules associated with common diseases, such as organ damage, inflammation, cancers, cardiovascular diseases, and brain disorders. We emphasize the strategies behind the design of fluorescent probes capable of disease biomarker detection and diagnosis and cover some aspects of combined diagnostic/therapeutic strategies based on regulating disease-related molecules. This review concludes with a discussion of the challenges and outlook for fluorescent probes, highlighting future avenues of research that should enable these probes to achieve accurate detection and identification of disease-related biomarkers for biomedical research and clinical applications.

## 1 Introduction

The effective treatment of a disease is predicated on its timely and accurate diagnosis that is dependent on the understanding of its development and pathology. Most relevant to the topic of this review are the chemical imbalances of key regulatory bioactive molecules that govern the normal function of all cells and living organisms.^1,2^ The ability to detect and monitor disease-related bioactive molecules is key to elucidating the molecular and biological mechanisms of a given disease, and to enabling the development of effective disease diagnosis and treatment methods.

Although this field remains dominated by traditional imaging methods such as computed X-ray tomography (CT),^3^ magnetic resonance imaging (MRI),^4^ ultrasonic imaging (US),^5^ and nuclear medicine imaging (e.g., positron emission tomography, single photon emission computed tomography (SPECT)),^6^ fluorescence imaging technologies now play an indispensable role in preclinical and basic research, enabling high sensitivity and high spatiotemporal resolution noninvasive real-time imaging.^7,8^ To improve the accuracy and sensitivity of fluorescence imaging and expand the scope of applications of optical imaging, researchers are continuously looking to develop new chemical tools that can detect an ever-increasing array of disease-related bioactive molecules to monitor key physiological processes. Termed fluorescent probes, a multitude have now been successfully developed^9,10^ and are used routinely in immunofluorescence staining, live-cell imaging, drug delivery, and fluorescence-guided surgery.^11−16^

**Figure 1:**
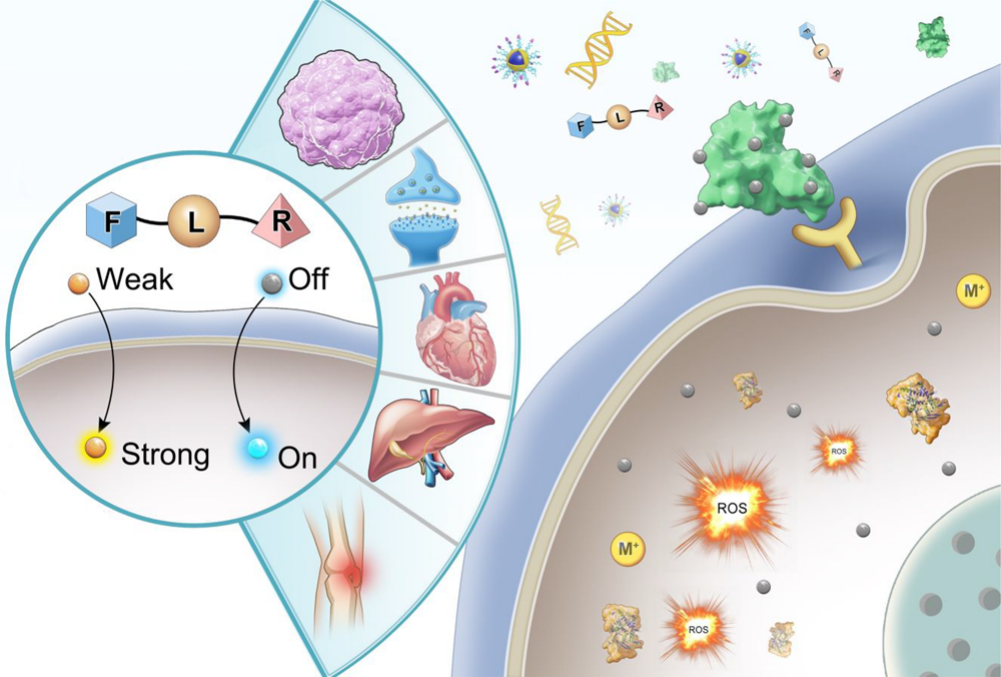
General structural features and design strategy of the fluorescent probes.

Structurally, a fluorescent probe is generally composed of three key elements: the recognition unit, the fluorophore (light-emitting substance), and the connector/linker. Additionally, some probes will also contain a targeting group, designed to interact with a specific tissue type or organelle, helping to localize the fluorescence signal (Figure 1).^17^

Simplest among them is the connector/linker, whose role is primarily to bridge the other components. The linker has a marked impact on the general properties of the probe, affecting for instance its overall size, diffusion rate *in vivo*, cell permeability, blood circulation time, etc. This module can be either stable under biological conditions, or labile—cleaving readily on detection of the desired analyte (examples of both will be seen throughout this review).^18−21^

The recognition unit enables the probe to “detect” the biomolecule by reacting with the desired analyte to elicit a fluorescent change. It is responsible for both specificity and selectivity, in essence the most important component of the disease-targeting design.

The fluorophore is the signal-generating part of the probe, with most fluorophores currently in use being either inorganic fluorescent materials or organic small molecule fluorescent dyes. Inorganic luminescent materials is normally based on either d- or f-metal ions, the latter can include rare earth elements, such as europium (Eu), terbium (Tb), samarium (Sm), erbium (Er), neodymium (Nd), etc.,^22,23^ or luminous quantum dots such as CdSe, CdTe, etc.^24^ Complementarily to these systems, a multitude of organic molecular fluorescent materials can be employed, boasting a wide range of varied (and variable) structures capable of producing tunable wavelengths suitable for almost any application. Commonly used organic fluorophore include cyanine dyes, rhodamines, coumarins, naphthalimides, borondipyrromethene difluorides (BODIPYs), and AIE (aggregation induced emission) fluorescent molecules such as tetraphenylethylene. The excitation and emission wavelengths of fluorophores are one of the primary considerations in probe design, with a focus on wavelengths outside of the range of tissue autofluorescence (approximately 300–700 nm).^25^ It is therefore unsurprising that the majority of clinically relevant optical imaging agents operate in the near-infrared (NIR) wavelength range (700–1700 nm), allowing tissue penetration of up to 5–10 mm.^26,27^ Many of these are now commercially available, covering the full range of NIR wavelengths, with longer-wavelength systems typically favored due to their improved signal-to-noise ratio.^28,29^

The field of fluorescent sensing has exploded over the last couple of decades, with an ever-increasing number of fluorescent probes for disease-associated bioactive molecule imaging being reported.^30−32^ Thanks to this transformative technology, the scientific community is now able to visualize physiological and pathological processes from the subcellular to the systemic level in real time, providing a powerful tool for clinical diagnosis and treatment.

The requirements for a successful disease-targeting/imaging fluorescent probe are manifold, including high cell/tissue specificity, high selectivity for the target biomarkers in the presence of competing species, photostability, biocompatibility, and long wavelength excitation (essential for *in vivo* imaging, *vide supra*).^33−35^ This review will focus on the latest developments in the field, looking closely at recent probes for targeting diseases in humans, including neurological diseases, cancers, organ damage, cardiovascular diseases, and inflammation. The relevant design strategies, working principles, and biomedical applications of these probes will be discussed, highlighting the need to identify individual biomarkers for different diseases, develop specific recognition reactions, and propose new fluorescent diagnostic and therapeutic strategies based on the regulation of bioactive molecules. This review will also highlight key challenges for developing more effective fluorescent probes with a view of applying fluorescence-based diagnostic imaging in clinical settings. We hope that this manuscript will help readers gain insight into the field and provide inspiration for the development of the next generation of fluorescent probes for human disease.

## 2 Fluorescent Probes for Neurological Diseases

As the command center of the body, the brain, spinal cord, and subsidiary nervous system components evidently play a key part in nearly every aspect of human biology.^36^ Minor changes in brain and nerve function can lead to sweeping issues and impairment, and so accurate and timely monitoring of key biomarker variations is critical.^37^ As such, a variety of fluorescent probes for neurological disease markers have been developed (see selected examples in Table 1), with a view of improving the diagnosis and treatment of neurological diseases. This section will look specifically at fluorescent probes for Alzheimer’s disease (AD), epilepsy, Wilson’s disease (WD), Parkinson’s disease (PD), depression, and stroke. Additionally, nanofluorescent probes for glioma will be discussed, as they represent an exciting new direction for the future development of relevant fluorescent probes.

**Table 1:** Selected Fluorescent Probes for Neurological Diseases

probe	λ_ex_/λ_em_ (nm)	LOD	bioactive molecule	biological model	ref
Alzheimer’s Disease					
**1**	565/635		Aβ fibrils	arcAβ mice	(42)
**2**	453/580		Aβ aggregates	HeLa cells, U87 cells, APP/PS1 transgenic mice	(43)
**3**	490/574	0.17 μM	H_2_O_2_	N2aSW cells, 5XFAD mice	(45)
**4**	560/589	45 nM	NO	HepG2 cells, SH-SY5Y cells	(46)
**5**	602/702	3.4 nM	ONOO^–^	PC12 cells, AD mouse	(47)
**6**	656/690	1.08 μg·mL^–1^	BChE and ROS	HEK293 cells, APP/PS1(B6) mice	(49)
**7**	600/710	16.8 nM	NFTs/Tau	SH-SY5Y cells, Tg-Tau mice	(50)
Epilepsy					
**8**	440//700	20.8 nM	Cys	BALB/c nude mice	(53)
**9**	540/750	43 nM	Cys in LDs and mitochondria	chronic epilepsy mice	(55)
**10**	460/685	151 nM	ONOO^–^	HT22 cells, rat epilepsy models	(56)
**11**	440/635	64.3 μM	norepinephrine	PC12 cells, epileptic mice	(57)
Wilson’s Disease					
**12**	670/800 553/– 823/–	80 nM	Cu^2+^	urine of WD patients	(59)
**13**	310/415	2.60 μM, 0.31 μM, 0.05 U/mL	Cu^2+^, pyrophosphate, alkaline phosphatase	HeLa cells	(60)
Depression					
**14**	700/780		polarity	PC12 cells, C57BL/6J mice	(65)
**15**	520/560	0.36 μM	AChE	PC12 cells, mice	(66)
**16**	390/460	0.32 μM	Zn^2+^, H^+^	PC12 cells, C57 mice	(67)
**17**	340/443	0.16 μM	Cys	PC12 cells, C57BL/6J mice	(68)
**18**	378/490	46 nM	Cys	HEK293 cells, depression mouse model	(69)
**19**	370/500	2.4 μΜ	OH•	PC12 cells, C57BL/6J mice	(70)
**20**, **21**	370/445; 610/670	2.413 mM (MI), 0.453 mM (LY)	H_2_O_2_	PC12 cells, C57BL/7J mice	(71)
**22**	570/690	10 nM	O_3_	RAW 264.7 cells, CUMS (chronic unpredictable mild stress) mouse model	(72)
**23**	620/685	222 nM	HClO	RAW 264.7 cells, PC12 cells, C57BL/6J mice	(73)
**24**	400/505	15 nM	HClO	PC12 cells, RAW 264.7 macrophages, zebrafish, mice	(76)
**25**	365/440,510	0.13 μM (MDA), 0.11 μM (FA)	malondialdehyde (MDA); formaldehyde (FA)	PC12 cells, C57 mice	(77)
**26**	720/750		norepinephrine	PC12 cells, depression mouse model	(78)
**27**	580/724		norepinephrine	PC12 cells	(79)
Parkinson’s Disease					
**28**	438/503	25.8 nM	HClO	SH-SY5Y cells, drosophila, PD mouse	(81)
**29**	510/670	4.59 nM	ONOO^–^	PC12 cells, SH-SY5Y cells, Parkin null *Drosophila*, WLZ3 *C. elegans*	(82)
**30**	395/500,650	0.27 μM	H_2_O_2_	living cells, zebrafish and *Drosophila*	(83)
**31**	630/770	0.48 μM	formaldehyde	PC12 cells, PD zebrafish, PD mice	(84)
**32**	385/516		viscosity, hydrogen sulfide	HeLa cells, PD mouse	(85)
**33**	343/464	0.4 μM	H_2_S	HeLa cells, DJ-1-KO mice	(86)
**34**	335/438		methionine sulfoxide reductase	PC 12 cells	(87)
Stroke					
**35**	500/557		ONOO^–^	RAW 264.7 cells, LPS-induced kidney injury of zebrafish	(90)
**36**	475/545	0.5 nM	ONOO^–^	EA.hy926 endothelial cells, intra_x005fluminal middle cerebral artery occlusion (MCAO) model	(91)
**37**	416/495	63.4 nM	viscosity/ONOO^–^	BV-2 cells, MCAO model	(92)
**38**	410/675		viscosity	BV-2 cells, MCAO model	(93)
**39**	440/544	0.017 μM	Fe^2+^	astrocyte cells, rat’s ischemic brain tissue	(94)
**40**	370/490		thioredoxin reductase	HeLa cells, zebra_x005ffishes, brain of mice with cerebral ischemia reperfusion injury (CIRI)	(95)
**41**	430/646	1.3 nM	H_2_S	PC12 cells, MCAO in living mice model	(96)
**42**	490/510	4.3 nM	glutathione	PC12 cells, MCAO in living mice model	(97)
**43**	736/1036		vascular	C57BL/6 mice, stroke mice model	(98)
Glioma					
**44**	980/448		N/A	U87MG cells, BCECs cells, glioblastoma-bearing mice	(100)
**45**	680/710		N/A	tiny brain glioma mice model	(101)
**46**	450/540		N/A	GBM cells, heterotopic glioma model	(102)
**47**	745/800		N/A	tumor-bearing mice	(103)
**48**	600/635		N/A	C6 cells, L929 cells, glioma-bearing mice	(106)
**49**	395/459		N/A	U87 cells, bEnd.3 cells, U87 cell xenograft-bearing mice	(107)
**50**	808/1055		N/A	C6 cells, glioma-bearing mouse	(108)
**51**	330/605		A32 DNA	U87 cells, HUVEC cells, human glioma tissues, orthotopic brain glioma model in mice	(109)
**52**	488/525		N/A	C6 cells, glioma-bearing mouse	(110)
**53**	550/570		N/A	C6 cells, bend.3 cells, glioblastoma bearing mice	(111)
**54**	808/1060,1340		N/A	U87-Luc cells, the tumor-bearing mice	(112)
**55**	397/1064		N/A	C6 cells, C57BL/6J mice	(113)

### 2.1 Fluorescent Probes for Alzheimer’s Disease

**Figure 2:**
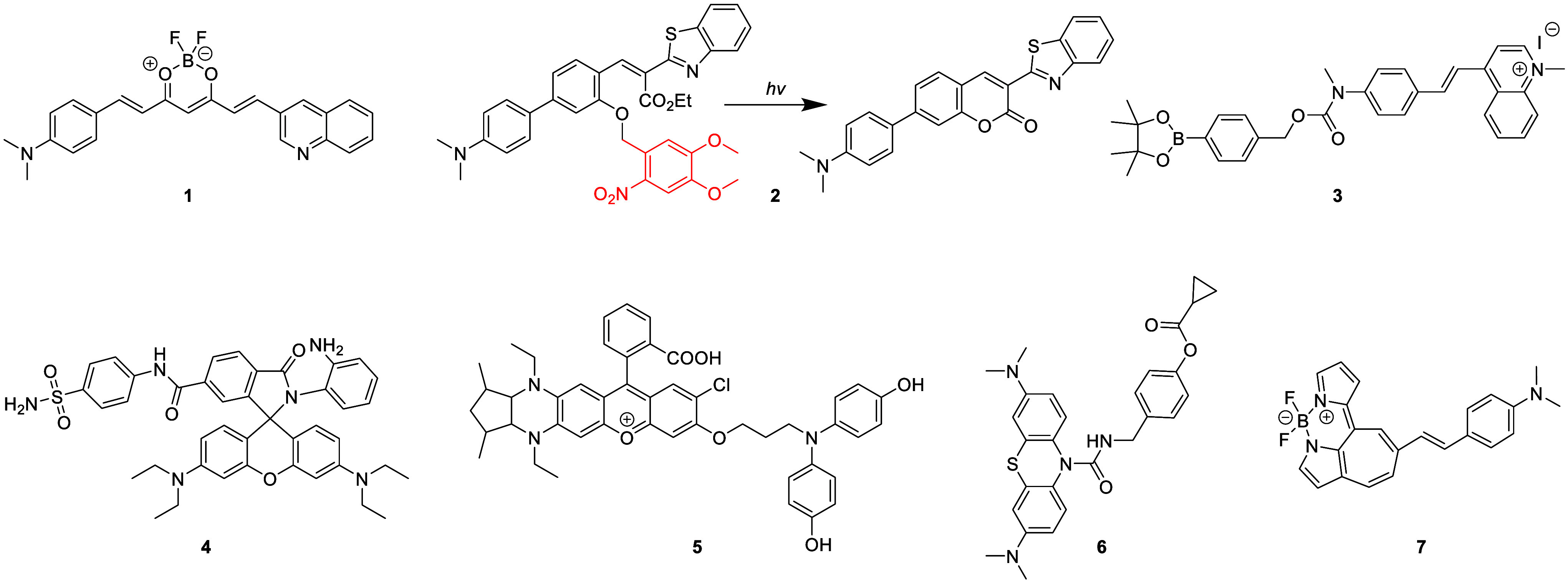
Selected fluorescent probes for Alzheimer’s disease.

Alzheimer’s disease (AD) is a progressive brain disorder that affects memory, thought, and behavior, eventually causing dementia and severe cognitive decline and decreased quality of life.^38^ AD is a complex condition, and although its pathogenesis remains unclear, it is currently believed to be caused by the abnormal accumulation of proteins (typically amyloid β, Aβ) in the brain, which form amyloid plaques, causing tissue death and breakdown of neural networks.^38^ Typical AD biomarkers targeted by fluorescent probes (Figure 2) therefore include Aβ, as well as general biomarkers of oxidative stress such as reactive oxygen species (ROS) and reactive nitrogen species (RNS).^39,40^

Aβ is a 36–43 amino acid polypeptide produced by the hydrolysis of amyloid precursor protein (APP) by β- and γ-secretase enzymes, whose deposition into amyloid plaques is closely associated with the onset and progression of AD.^41^ A recent example of an Aβ-specific fluorescent probe is the curcumin derivative probe **1** developed by Qian and co-workers in 2021,^42^ capable of multispectral photoacoustic tomography and fluorescence imaging of brain amyloidosis. The authors showed that probe **1** can specifically and quantitatively detect Aβ fibrils, distinguishing said fibrils from the monomeric form of Aβ. Immunohistochemical studies confirmed colocalization of probe **1** and Aβ deposits in the brain sections of arcAβ mice, demonstrating the high specificity of the probe. Probe **1** was successfully shown to detect Aβ deposits in animal models of AD pathology and could be employed to monitor the effects of Aβ treatment longitudinally and reveal the mechanisms of disease evolution.

Similarly, in 2022, probe **2** was developed by Wang et al.,^43^ and was found to be capable of detecting Aβ aggregates through a “photo-triggered” fluorescence mechanism, rather than simply colocalization. In this instance, the 6-nitroveratryl protecting group is photocleaved upon irradiation, causing cyclization of the resulting phenolic group onto the adjacent ester, which produces a fluorescent coumarin core. The newly formed fluorophore is weakly fluorescent and capable of binding with Aβ to produce a strong localized fluorescent response. Probe **2** was shown to detect Aβ aggregates *in vivo* in an AD model (APP/PS1 transgenic) mouse, with good blood–brain barrier (BBB) permeability after light exposure. This innovative “phototrigger” approach has the potential for use in designing targeted phototriggered dyes/fluorophores for the detection of specific proteins with a high signal-to-noise ratio.

Another prominent cause/marker of AD is oxidative stress, triggered by an imbalance in the production and accumulation of ROS or RNS.^44^ In 2023, Wong and co-workers^45^ developed fluorescent probe **3** that both targets Aβ and responds to changes in hydrogen peroxide (H_2_O_2_) concentrations in live cells and in AD mouse models in real time. This highly sensitive probe uses a methylquinolinium-based fluorescent unit, and a phenylboronate pinacol ester (BPin) as the H_2_O_2_ recognition unit. Probe **3** is fluorescent at relatively long wavelengths (λ_em_ = 574 nm) and can be used to locate Aβ directly. Once bound to the plaque, it is susceptible to reaction with H_2_O_2_, which oxidizes the BPin, leading to cleavage and release of a new species which emits at a longer wavelength (λ_em_ = 660 nm), enabling ratiometric visualization and measurement of H_2_O_2_ concentration changes at Aβ sites. This probe was shown to successfully image real-time changes in hydrogen peroxide content induced by Aβ species in both neuronal cells and in AD mouse models.

In 2022, Ma and colleagues^46^ developed Golgi-targeting fluorescent probe **4**, which can detect nitric oxide (NO) in AD mice. Probe **4** is composed of three main units: a 6-carboxyrhodamine B as the fluorophore, a 4-sulfamoylphenylamide as the Golgi-targeting group, and an *o*-diaminobenzene as the NO-sensing motif. This probe shows both impressive Golgi-targeting abilities and high specificity for NO detection. Using probe **4**, the researchers were able to note a significant increase in NO levels within the Golgi apparatus in Aβ-induced AD. Probe **4** therefore provides a valuable new tool for the *in situ* imaging of NO in the Golgi apparatus, possibly shedding light on the significance of NO in disease-related signaling pathways. Furthermore, this probe demonstrates the “modular design concepts” of fluorescent probes, wherein simple substitution of the 2-carboxyl reactive motif for another biological targeting unit designed to recognize another bioanalyte should allow for visualization of a variety of additional biomolecules in the Golgi apparatus.

**Figure 3:**
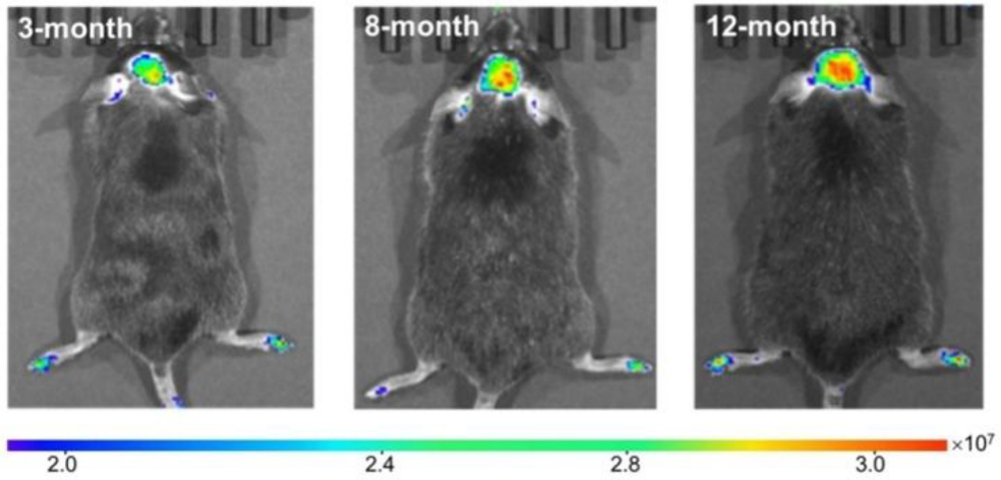
*In vivo* fluorescence imaging of AD mice of different ages (3, 8, and 12 month) by tail injection of probe **5**, showing increased ONOO^–^ concentrations in the brain with age. Reproduced with permission from ref (47). Copyright 2022 John Wiley & Sons.

Kim and co-workers created probe **5** in 2022, a NIR fluorescent probe for peroxynitrite (ONOO^–^).^47^ Probe **5** contains diamino-substituted rhodol dye NIR-Rd-3 as the fluorophore with the 4-aminophenol group acting as the ONOO^–^ recognition unit. This enabled *in situ* imaging of ONOO^–^ in AD mice (Figure 3), and allowed Kim et al. to demonstrate that ONOO^–^ can serve as a biomarker for AD.

Another salient target for AD diagnosis and study is the enzyme butyrylcholinesterase (BChE), whose levels are known to progressively increase during the course of the disease.^48^ In 2021, Ding et al.^49^ designed and synthesized the dual BChE and ROS “logic-gate” based fluorescence probe **6**, containing a BChE-specific cyclopropyl formate group and amide/ester bonds as ROS-reactive motifs. Only upon reacting with both ROS and BChE was the fluorescence turned on, creating a highly selective dual-analyte probe with excellent sensitivity (LOD = 1.08 μg·mL^–1^). This probe was shown to be suitable for imaging in both cells and early AD mice. Interestingly, this work also detailed similar fluorophores with variable linker length, demonstrating that a single methylene unit between the urea and phenol motifs was optimal in this instance.

Neurofibrillary tangles (NFTs), composed of abnormally hyperphosphorylated tau proteins, are also a hallmark of AD and other tauopathies. In 2022, Cui and co-workers^50^ developed the fused cycloheptatriene-BODIPY derivative probe **7**, which was used for high specificity NIR imaging of NFTs. It was found that probe **7** could efficiently cross the BBB and specifically bind to NFTs, recognizing them readily in the brains of tau mice.

### 2.2 Fluorescent Probes for Epilepsy

**Figure 4:**
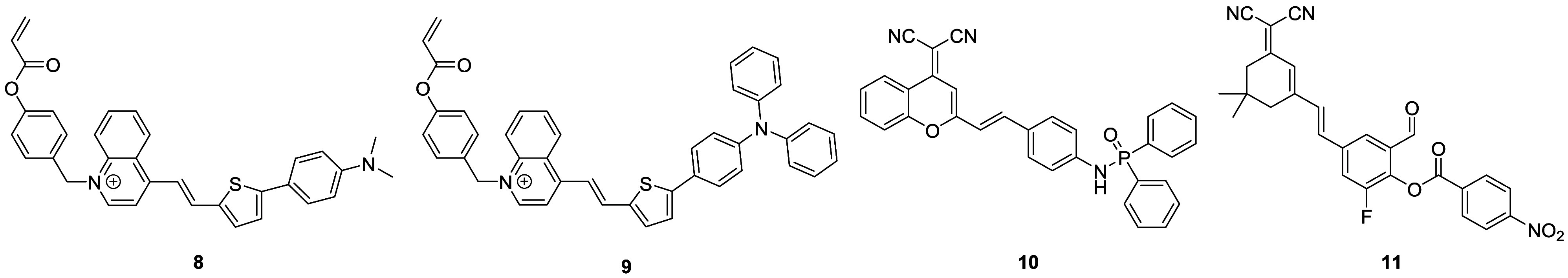
Selected fluorescent probes for epilepsy.

Fluorescent probes have also emerged as a popular method for studying epilepsy (Figure 4), a neurological disorder that causes uncontrolled seizures or convulsions due to electrical disturbances in the brain.^51^ These seizures can cause a wide range of symptoms, including loss of consciousness, muscle spasms, and sensory disorders. Unfortunately, epilepsy remains very difficult to treat, driving a need for new and better treatments, promoting research into fluorescent probes for the imaging and mechanistic understanding of epilepsy.

**Figure 5:**
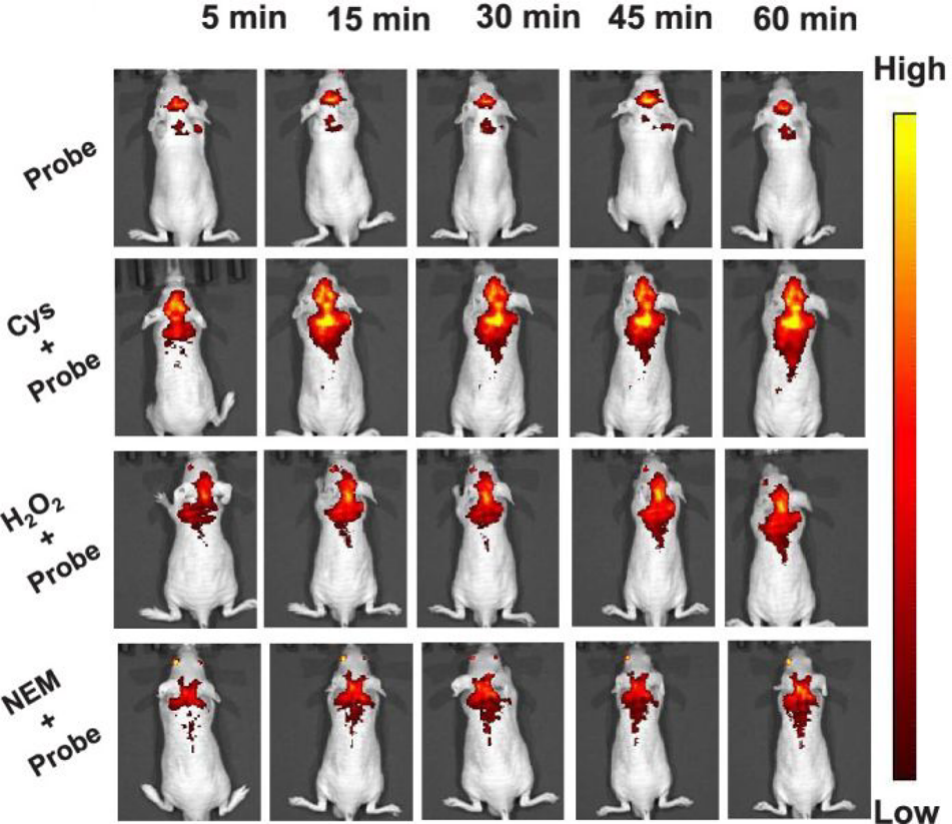
Mapping of Cys fluxes at 5, 15, 30, 45, and 60 min in live mice with probe **8** after intraperitoneal injection of various Cys-affecting agents. Reproduced with permission from ref (53). Copyright 2020 American Chemical Society.

As with AD, one of the hallmarks of epilepsy is oxidative stress within the brain.^52^ One way of studying oxidative stress is through the monitoring of the thiol cysteine (Cys), a key biological reducing agent directly involved in intracellular regulation of ROS. In this context, it has been proposed that decreased cysteine levels in the plasma might serve as a biomarker of temporal lobe epilepsy, as it might indicate sustained oxidative stress. In 2020, Li and co-workers^53^ reported probe **8**, a NIR-emitting imaging probe for tracking endogenous Cys in the brain during pentylenetetrazole (PTZ)-induced seizures. Probe **8** was constructed around the Mito-Q fluorophore, composed of a *N*,*N*-dimethylamino electron donor, a quinoline cation electron acceptor (this system being thus based around a *push*–*pull* intramolecular-charge-transfer, ICT, motif), and an acrylate motif (a Michael acceptor) as the Cys recognition site. Probe **8** exhibited good BBB penetration, quickly entering the brain to map Cys after intravenous injection. The ability of probe **8** to detect changes in Cys concentration, whether externally added or endogenously produced by oxidative stress, was demonstrated in different organisms, including cultured cells, zebrafish, and mice (Figure 5). These results provided new insights into the relationship between mitochondrial Cys levels and the onset, development, and treatment of epilepsy.

Another fluorescence mechanism that has been harnessed for Cys imaging is the use of aggregation-induced emission (AIE); a phenomenon by which molecules emit light only when they aggregate (or come together in close proximity).^54^ To this end, He et al. developed probe **9** in 2023,^55^ as an AIEgen-based dual-channel Cys-responsive NIR fluorescent probe for imaging in lipid droplets and mitochondria. Structurally related to **8**, probe **9** is constituted of three parts: a triphenylamine group serves as a lipid droplet-targeting moiety, a quinolinium group acts as a mitochondrial-targeting moiety, and an acrylate motif as before for Cys-specific recognition. Using probe **9**, He et al. were able to monitor (dual-channel) Cys in mitochondria and lipid droplets during cell apoptosis by using wash-free fluorescence bioimaging. Utilizing probe **9**, the authors monitored apoptotic events in epilepsy, showing that mitochondrial Cys plays a vital role in epilepsy, possibly revealing a new target for treatment and diagnosis of the condition.

As one of the major markers of oxidative stress, ONOO^–^ overexpression can be used as a highly informative indicator for the early diagnosis of epilepsy. With this in mind, Yu and co-workers^56^ developed NIR fluorescent probe **10** in 2021, able to track changes in ONOO^–^ levels in cells and *in vivo*. The probe is composed of a NIR dicyanomethylene-4*H*-pyran (DCM) fluorophore unit and a diphenylphosphinamide recognition moiety. On exposure to ONOO^–^, the phosphinamide P–N bond is cleaved, releasing a free electron-donating aniline to generate an ICT fluorescence system, which is associated with a significant increase in fluorescence emission at 685 nm. Probe **10** can also be used to track ONOO^–^ in epilepsy, specifically endogenous ONOO^–^ in the hippocampus of epileptic rat models induced by kainate. This probe was used to track the effects of the antioxidant compound resveratrol, with increased concentration of this agent causing a sharp decrease in fluorescence intensity, indicating a clear inhibition of ONOO^–^ overexpression. Probe **10** can therefore serve as a powerful tool, offering great potential for real-time tracking of ONOO^–^ fluctuations in nerve tissue, further aiding the diagnosis of epileptic disorders.

Finally, norepinephrine (noradrenaline, NE) is also a key biomarker of many neurological disorders, including epilepsy, as it is one of the main regulatory neurotransmitters. The lack of specificity that has previously plagued NE sensing was in part overcome by Yin et al. in 2023,^57^ who used an innovative “hunting–shooting” design strategy with probe **11**. The fluorophore in this instance is a 2-(cyclohex-2-en-1-ylidene) malononitrile derivative, with a pendant aldehyde acting as the reactive unit. Following reaction of the aldehyde with NE, loss of the 4-nitrobenzoate fluorescence-masking group is triggered, releasing a highly fluorescent species. This probe was able to successfully penetrate the BBB and was used to observe changes in the NE release within the brains of mice before and after epilepsy. This allowed anatomical studies of brain tissue to map distribution and level changes of NE in different regions of the brain before and after epilepsy.

The above cases represent excellent examples of successful approaches to the development of fluorescent probes capable of crossing the BBB and effectively imaging hard-to-sense biomarkers, thus provide promising tools for the diagnosis and study of the pathogenesis of diseases of the nervous system.

### 2.3 Fluorescent Probes for Wilson’s Disease

**Figure 6:**
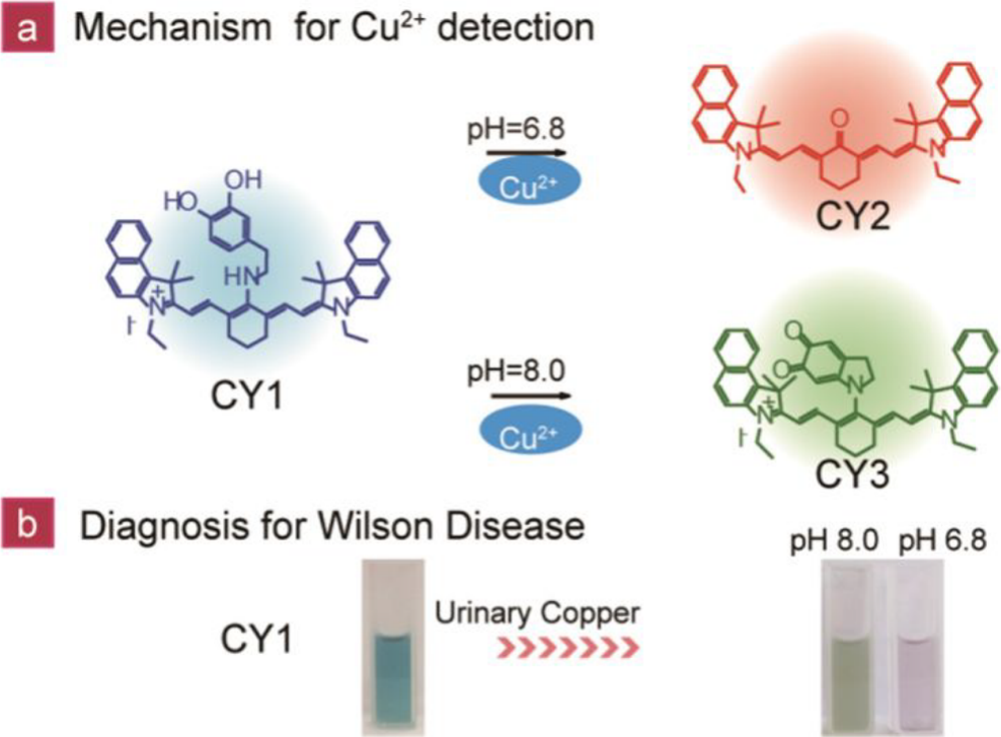
(a) Oxidation of **CY1** (**12**) to **CY2**/**CY3** by Cu^2+^ in acidic and alkaline solutions. (b) Use of **CY1** as a copper ion detection probe in urine from WD patients, showing a change from blue to green (alkaline) or pink (acidic) on detection of Cu^2+^. Reproduced with permission from ref (59). Copyright 2018 American Chemical Society.

Hepatolenticular degeneration, termed Wilson’s disease (WD), is an autosomal-recessive disease which causes excessive accumulation of copper in the body and increased excretion of urinary copper.^58^ Because urinary copper detection is part of routine examinations of WD patients, in 2018, Li et al.^59^ developed a dual colorimetric/fluorescent probe **12** (shown as **CY1** in its initial form in Figure 6) for detecting Cu^2+^, where a dopamine group acts as the coordinating/detecting group for Cu^2+^, and a cyanine dye is the fluorophore. In slightly acidic solution (pH < 6.8), the **CY1** form of **12** (λ_ex_ = 670 nm, λ_em_ = 800 nm) reacts oxidatively with Cu^2+^ to form **CY2** (λ_ex_ = 553 nm). In basic solution, **CY1** can be oxidized to form **CY3** (λ_ex_ = 823 nm) (Figure 6). The difference in absorption wavelengths of all three species allowed the concentration of Cu^2+^ to be monitored by UV–vis–IR spectroscopy, with exposure to Cu^2+^ leading to both marked color changes (change in absorption) and decrease in fluorescence signal from **CY1**. Thus, probe **12** is clearly a highly effective and promising colorimetric/fluorescent probe for the detection of Cu^2+^ in urine.

**Figure 7:**
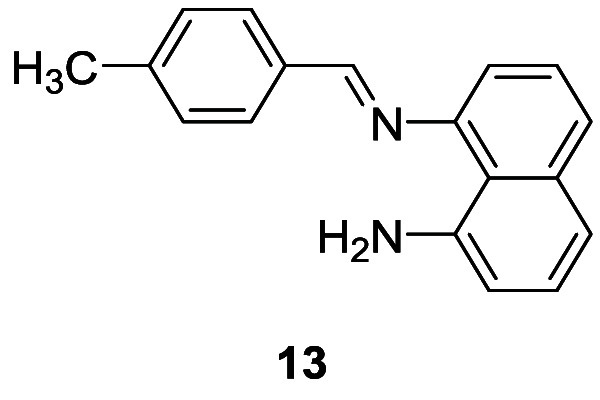
Schirhagl et al.’s probe **13** for sequential detection of Cu^2+^, PPi, and ALP.

Other biologically significant markers of WD include pyrophosphate (PPi), a product of the metabolism of adenosine triphosphate (ATP), and alkaline phosphatase (ALP), a dephosphorylating enzyme that effects this transformation. Raised concentrations of PPi and low levels of serum ALP are commonly observed in WD patients, with both of these biomarkers being currently used to diagnose the disease. In 2019, taking all three biomarkers into account, Schirhagl et al.^60^ designed probe **13** that was found to be capable of sequential and selective detection of Cu^2+^, PPi, and ALP *in vitro*, in living cells, and in synovial fluid samples with “off–on–off” fluorescence signals. The rather simple structure of probe **13** acts as a bidentate ligand, chelating Cu^2+^ between the imine and aniline nitrogen atoms, switching the fluorescence signal “off”. However, PPi has a stronger binding affinity to Cu^2+^ than **13**, so subsequent addition of PPi causes dissociation of the metal from the probe, which leads to a “turn-on” fluorescence response (Figure 7). Addition of ALP results in the hydrolysis of PPi, disrupting the PPi–Cu^2+^ complex, releasing Cu^2+^ which is chelated again by probe **13**, once again suppressing the fluorescence signal. It was hoped that by using this innovative fluorescence “off–on–off” approach, probe **13** could provide valuable insights into the balance of Cu^2+^, PPi, and ALP, and thus be used in the future diagnosis and study of WD.

### 2.4 Fluorescent Probes for Depression

**Figure 8:**
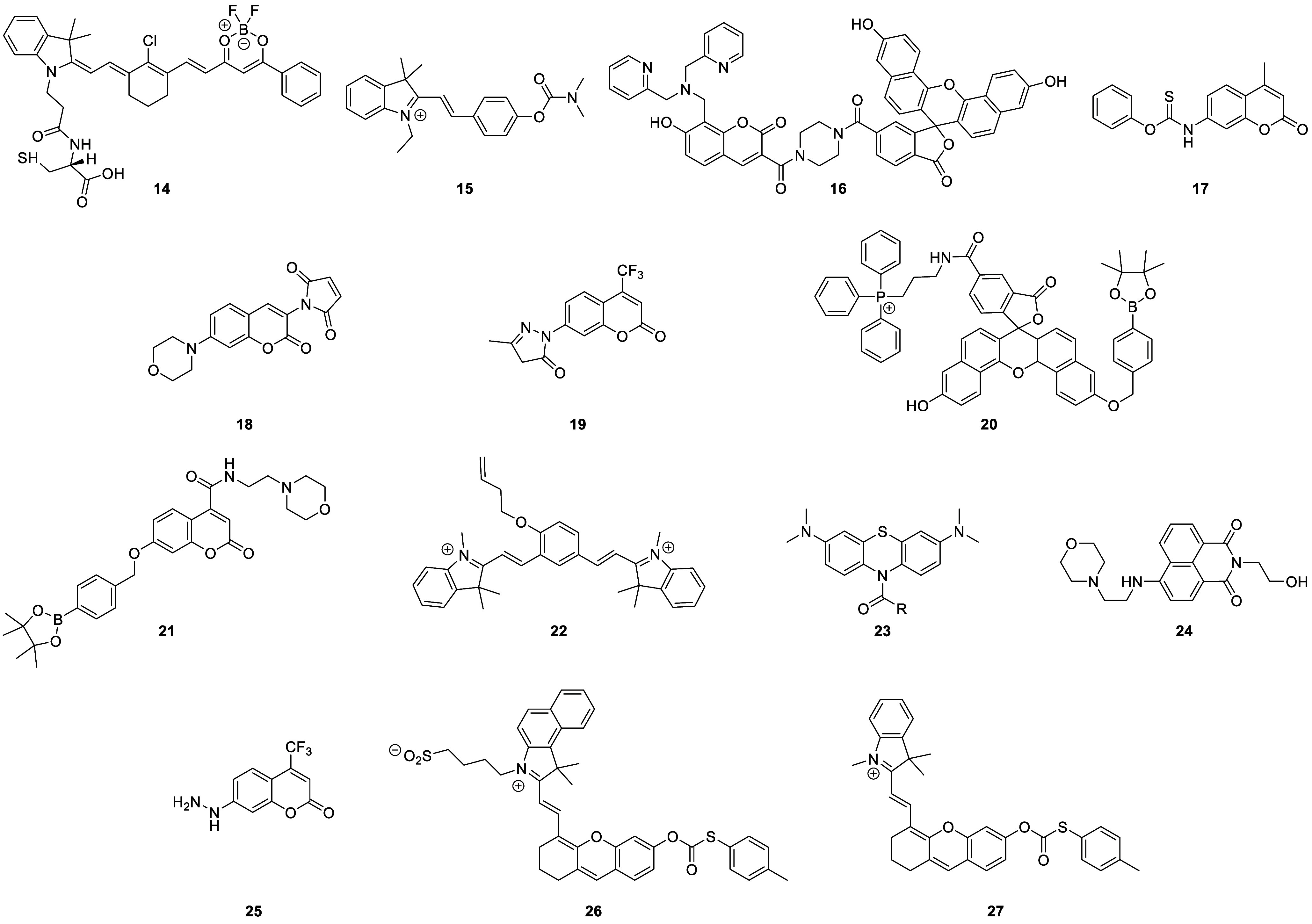
Selected fluorescent probes for depression.

Depression is a neurological disorder characterized primarily by a persistently low mood, and patients with major depressive disorders suffer from generally reduced quality of life and are at serious risk for self-harm.^61^ Unfortunately, the pathogenesis of depression is essentially unknown, severely hindering progress in developing new treatments (e.g., antidepressants). Enormous efforts are therefore ongoing to try and understand the molecular and biological mechanisms of depression,^62−64^ including the development of a multitude of new probes for the sensing, visualization, and tracking of depression-correlated biomarkers (Figure 8).

A key factor identified as having a close link to the onset and progression of depression is brain-derived neurotrophic factors (BDNF, also known as abrineurin). As the Golgi apparatus plays a vital role in the processing of proBDNF, it is believed that its microenvironment, including its polarity, could be closely linked to BDNF levels, and as such, changes in the polarity of the Golgi apparatus may be indicative of the development of depression. Therefore, to monitor this factor, in 2019, Tang and co-workers reported cysteine-derived probe **14**, which targets the Golgi apparatus and detects its polarity using merocyanine and benzoyl difluoroboronate moieties as electron-donating and accepting groups, respectively, which facilitate excited state ICT.^65^ In polar environments, the excited state energy can dissipate due to dipole–dipole interaction between the solvated probe and the solvent, resulting in weak fluorescence arising from probe **14**. However, in nonpolar media, stronger fluorescence is observed as the polarity decreases. By using a l-cysteine targeting motif, this probe was able to specifically target the Golgi apparatus to detect these polarity changes in depression, and it was discovered that the brains of depressed phenotype mice had a significantly higher Golgi apparatus polarity than nondepressed mice, which may result in reduced BDNF synthesis, potentially providing a new method for diagnosing depression and an innovative tool for exploring its occurrence and development mechanisms.

**Figure 9:**
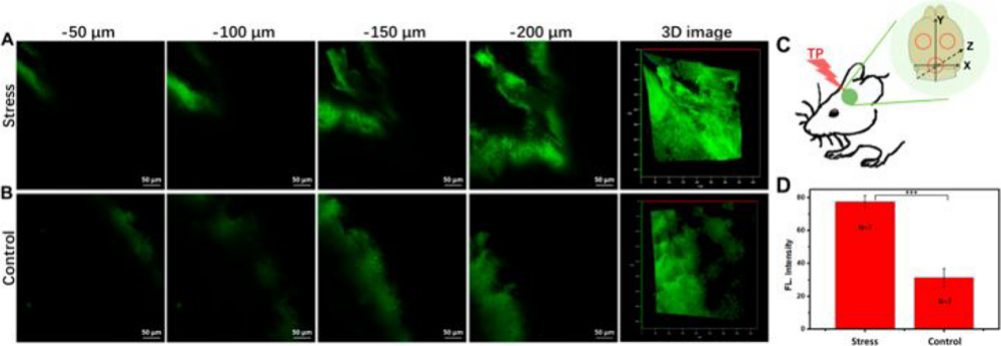
*In situ* TP fluorescence imaging with probe **15** in the brains of stress (A, 14 consecutive days of chronic-restraint stress) and control (B) mice. (C) Sketch of three different TP fluorescence imaging areas. (D) Relative fluorescence intensities of mice in A and B. Fluorescence emission window: 480–650 nm. Scale bar = 50 μm. Reproduced with permission from ref (66). Copyright 2019 American Chemical Society.

Another factor associated with depressive symptoms is acetylcholinesterase (AChE), with AChE inhibitors shown to be effective in alleviating depressive symptoms in some individuals. To detect AChE in the brains of depressed mice, the two-photon (TP) fluorescence probe **15** was developed in 2019.^66^ This probe uses neostigmine, an inhibitor of AChE, as the AChE recognition group and merocyanine as the fluorophore. Probe **15** itself exhibits only weak fluorescence due to the masking of the phenol group with a dimethyl carbamate reducing its electron-donating ability, thus suppressing the ICT-based *push–pull* electronic effect of merocyanine required for fluorescence. On exposure to AChE, the ester is cleaved/hydrolyzed, concomitantly releasing a strongly donating phenol to produce the active fluorophore. Probe **15** exhibited excellent selectivity for AChE. Furthermore, acetylcholinesterase activity was found to be positively correlated with the depressive phenotype (Figure 9). Therefore, probe **15** can be used as an effective tool to explore acetylcholinesterase-related diseases and provide valuable information for the treatment of depression.

The *N*-methyl-d-aspartic acid (NMDA) receptor, a subtype of ionic glutamate receptors and an ion channel protein, plays a crucial role in the development of neurons and synaptic plasticity. Regulatory binding partners of NMDA receptors such as Zn^2+^ and H^+^ are closely associated with NMDA receptor activity and consequently are expected to affect depression. To explore this relationship, a two-color fluorescent probe **16**, was developed in 2020 to simultaneously monitor Zn^2+^ and H^+^ levels in the brains of depressed mice.^67^ The probe incorporated fluorescein as the fluorophore, DPA (2,2′-dipicolylamine) as a zinc-coordinating Zn^2+^ recognition group, and naphthalene fluorescein as an acid-sensitive proton recognition unit. It was shown that the DPA motif quenches the fluorescence of the coumarin core through photoinduced electron transfer (PeT). Upon binding to Zn^2+^ PeT is blocked, allowing for bright blue fluorescence at 460 nm. Meanwhile, upon reaction with H^+^, the red fluorescence intensity at 680 nm decreased due to the reversible acid-catalyzed ring closure of the naphthofluorescein group from the open fluorescent quinone to the closed nonfluorescent spironolactone forms. Results of this study indicated an increase in both Zn^2+^ ad H^+^ levels in PC12 cells under oxidative stress. Additionally, both Zn^2+^ concentration and pH were found to be reduced in the brains of mice with depression-like behavior, which suggests that changes in Zn^2+^ and H^+^ levels, and therefore NDMA receptor activity, could be linked to depression.

As already shown in earlier examples, excessive production of ROS can cause oxidative stress in the brain, resulting in damage to a host of biomolecules such as proteins and nucleic acids, which may contribute to the development of depression. Imaging of the endogenous reductant Cys (*vide supra*) can therefore be used to indirectly assess oxidative stress. With this in mind, the TP fluorescence probe **17** was developed in 2020.^68^ Upon selective nucleophilic addition of Cys to the thiocarbonyl, a stable five-membered thioazoline ring is formed, creating a (ICT-based) *push–pull* electronic effect with the coumarin which increases fluorescence. With this probe, Cys levels could be tracked in the brains of mice with depression-like behavior to establish a negative correlation between Cys levels and the degree of depression-like behavior, consistent with a positive correlation between increased ROS/oxidative stress and depressive states.

In 2022, Ma et al.^69^ investigated alternatives for tracking Cys in depression, developing fluorescent probe **18** that specifically monitors Cys. With coumarin as the fluorophore and a maleimide group as the Cys-recognition group, the fluorescence emission is suppressed by PeT between the fluorophore and maleimide group. On Michael addition of the thiol to the maleimide, PeT is weakened, restoring fluorescent properties to the probe. Ma and co-workers observed a negative correlation between the level of Cys and the degree of depression, as was seen with probe **18** above. The design principles illustrated in these two complementary studies illustrate the overall approach and highlights the ease with which such probes can be readily developed for the study of biomercaptans in neural diseases, specifically Cys-related depression in this case.

**Figure 10:**
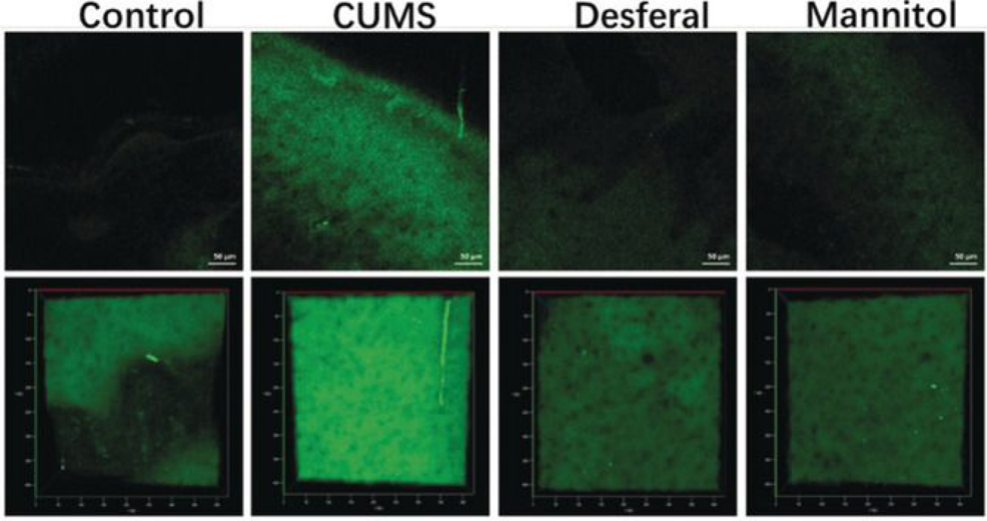
*In situ* TP imaging of hydroxyl radical by probe **19** in mice. Control: the mice without CUMS. CUMS: the mice susceptive to CUMS. Desferal: The susceptible mice injected with desferrioxamine. Mannitol: The susceptible mice injected with mannitol. The fluorescence images were obtained with an 800 nm light source. The 3D images (second row) were generated from a stack of cross sections (*xy* sections, 400 μm) with an axial (*z*) increment of 2 μm. Fluorescence emission windows: 400–650 nm. Scale bar = 50 μm. Reproduced with permission from ref (70). Copyright 2019 John Wiley & Sons.

Of the many ROS found endogenously, hydroxyl radical (•OH) has the highest oxidative capacity, able to severely damage biomacromolecules, accelerate cell aging, and, ultimately, lead to neurological diseases. Understanding the link between variations in •OH concentration and depression could provide insights into the molecular mechanisms of this disorder. To this end, probe **19** was developed for the detection of •OH in 2019.^70^ Here, a coumarin bearing a β-trifluoromethyl substituent was used as the fluorophore, with this group acting as a strong inductively electron-withdrawing group to both enhance the coumarin’s *push–pull* effect and increase its lipophilicity to help it cross the BBB; both desirable features for this type of imaging agent. In this system, hydroxyl radical-driven one-electron oxidation of the 3-methyl-pyrazolone recognition unit lead to pyrazolone ring-opening, creating a potent ICT fluorescence system, with a large increase in the fluorescence emission. Increases in •OH content in the brains of mice with depression-like behavior, were successfully imaged (Figure 10) using probe **19**. This work was used to suggest/support that inactivation of SIRT1 by •OH could account for the depressive phenotype, demonstrating that probe **19** can prove a useful tool for exploring •OH-related diseases and in helping to investigate the molecular mechanism of depression.

Continuing with the imaging of ROS in depression models, a number of probes have been developed to image ROS other than •OH, such as H_2_O_2_, ozone (O_3_), hypochlorous acid (HClO), or hypobromous acid (HBrO). For instance, fluorescent probes **20** and **21** were developed in 2022 to detect H_2_O_2_ in mitochondria and lysosomes, respectively.^71^ Both are based on the popular benzyl pinacolboronate ester peroxide recognition units discussed previously, with probe **20** built around fluorescein (as the fluorophore) and triphenylphosphonium as the mitochondrial-targeting moiety, while probe **21** is composed of a coumarin fluorescent core and a morpholine based lysosome-targeting unit. Fluorescence imaging revealed that mitochondrial H_2_O_2_ mediates decreased glucoencephalosidase activity in the lysosomes of the brains of mice.

Ozone, itself a ROS, is known to react with unsaturated fatty acids via ozonolysis, generating multiple new reactive carbon-rich ROS that can cause further damage to key cellular components. In 2019, ozone was directly observed in the brains of mice using the NIR fluorescent probe **22**.^72^ This probe utilizes a cyanine-7-type core as the precursor of the fluorophore and a 3-butenyl functionality as the unsaturated “recognition” group, which reacts with O_3_ via a cycloaddition (ozonolysis). The system undergoes a specific cycloaddition reaction with the terminal olefin of the 3-butenyl group, causing oxidation, fragmentation, and rearrangement to produce the associated quinone. This results in an enhanced degree of unsaturation, causing a bright NIR fluorescence emission to be produced on reaction with O_3_. *In situ* imaging of O_3_ in the brain tissue of mice with depression phenotypes exhibited an increase when compared to normal mice, and ozone appeared to induce depression by triggering excess IL-8.

Multifunctional fluorescence platform **23** has enabled the monitoring of HClO, a useful method for indirectly assessing the release of neurotransmitters and effects of antidepressants.^73^ This platform was built around methylene blue (MB), known to have excellent anti-inflammatory properties and optical characteristics, with neurotransmitters or antidepressants covalently linked to MB through a urea linkage designed to be specifically cleaved only by HClO.^74,75^ Behavioral tests and biochemical analysis indicated that probe **23** effectively reduced ROS levels, alleviated oxidative stress/inflammation, and reduced symptoms of depression in mice. Compared to commonly used antidepressants, probe **23** exhibited better antidepressant effects, fewer side effects, and a shorter treatment time due to the synergistic treatment strategy. In addition, probe **23** successfully achieved preliminary diagnosis of depression in mice. This innovative HClO-triggered fluorescence strategy should provide a new and promising platform for the diagnosis and treatment of depression.

The analogous bromine-based hypobromous acid is also of concern, and so the TP fluorescence probe **24** was designed in 2022 for real-time monitoring and visualization of HOBr levels in living systems.^76^ This probe is built around a 1,8-naphthalimide fluorophore and *N*-(2-aminoethyl)-morpholine lysosome-targeting group, with the 1,2-aminoethanol motif aiding with solubility. Upon oxidation of the morpholine by HOBr, S_E_Ar is triggered to generate a polysubstituted fluorescent naphthalimide. This probe boasts excellent selectivity, a fast response time (5 s), and high sensitivity (LOD = 15 nM). Probe **24** was shown to successfully detect increased HOBr levels in a range of other systems, such as inflamed tissue, breast cancer models, as well as brains of mice with depression.

Malondialdehyde (MDA) and formaldehyde (FA) are highly reactive, toxic, and lipophilic reactive carbonyl species (RCS) that can easily penetrate the BBB, causing protein dysfunction within the brain, potentially leading to the development of brain diseases such as depression. To address this issue, a TP fluorescence probe **25**, which has the ability to detect MDA and FA simultaneously with spectrally resolved signals, was developed in 2022.^77^ The hydrazine group of probe **25** was used as the recognition motif, reacting with MDA to create a pyrazole, and with FA, to form a hydrazine group, allowing for accurate and facile discrimination between MDA and FA. As with probe **19**, introduction of the trifluoromethyl group was expected to enable the probe to cross the BBB for visualization of both MDA and FA in live tissue. This was achieved, and probe **25** was employed for simultaneous imaging of both MDA and FA in living cells and *in vivo*, demonstrating for the first time that higher concentrations of MDA and FA are found in the brains of depressed mice than in normal healthy mice.

Norepinephrine levels also have a close association with depression, and so in 2023 Zhao et al.^78^ synthesized probe **26**, which enabled the NIR fluorescent photoacoustic (PA) imaging. In designing this probe, a cyanine was used as the fluorophore, adding a sulfonic acid group for solubility and biocompatibility, while using the phenolic hydroxyl group as the reactive site, by functionalization with an NE-selective tolylthioester motif. Brain visualization techniques employing probe **26** could therefore be used to diagnose depression in model mice and monitor the effects of drug intervention on NE levels.

A very similar probe, **27**, was developed by Yin et al. in 2022.^79^ Although no significant difference in baseline norepinephrine content between “normal” and “depressed” cells was observed, when exposed to high potassium levels depressed cells were shown to secrete less NE than normal cells. This study also evaluated the effects of antidepressants and G-protein-coupled receptor antagonists on depressed and normal cells, with findings suggesting that depression is associated with exocytosis of NE, and that inhibition of NE receptors may affect its release.

### 2.5 Fluorescent Probes for Parkinson’s Disease

**Figure 11:**
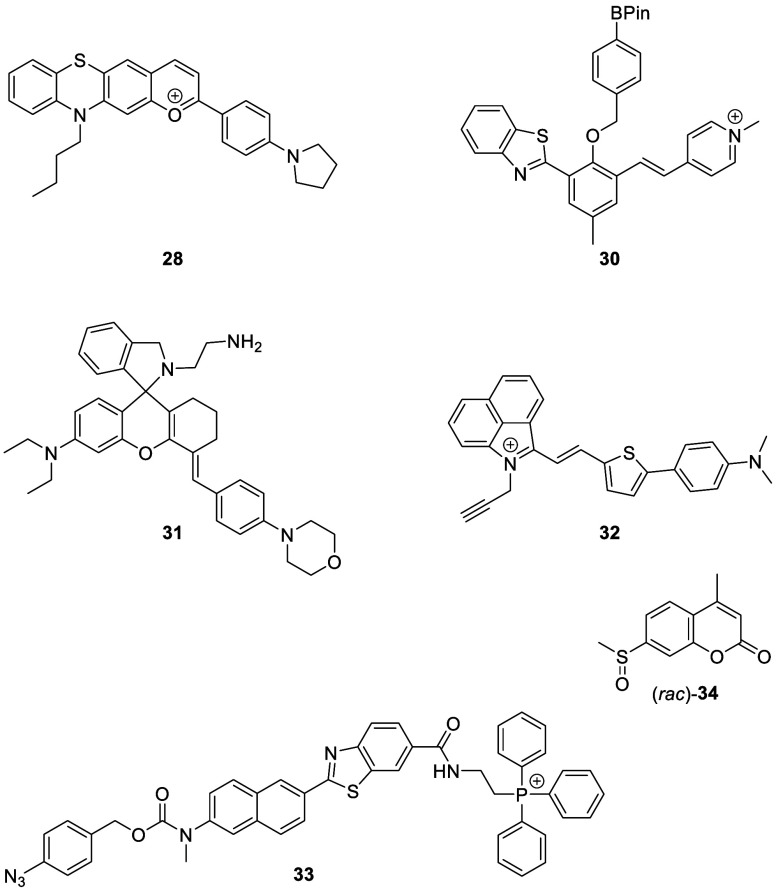
Selected fluorescent probes for Parkinson’s disease.

Parkinson’s disease (PD) is a neurodegenerative disorder characterized by clinical signs of motor deficiencies with resting tremor and/or limb paralysis. Currently, treatments for PD are limited and significantly improved approaches need to be developed.^80^ As such, suites of fluorescent tools for the imaging relevant molecular biomarkers are actively being developed (Figure 11).

**Figure 12:**
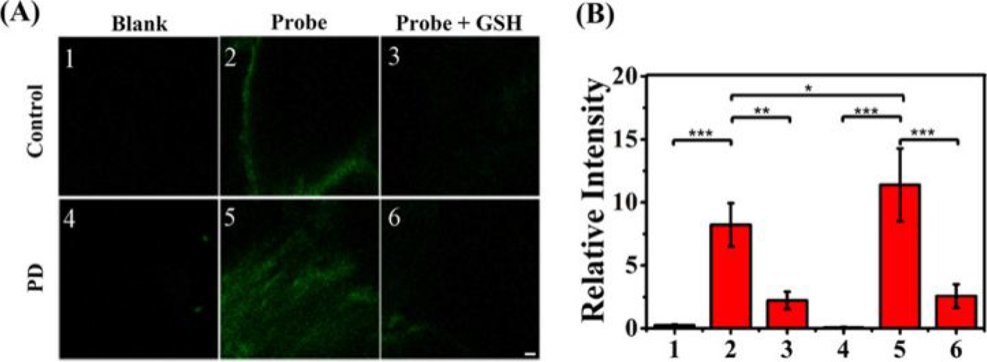
(A) Confocal fluorescence imaging of probe **28** in the substantia nigra region of the control and PD mouse brains. Brain slices were incubated with NUU-1 (50 μM), 0.5% ethanol, and 0.1% Triton in PBS (pH = 7.4) for 2 h and then further incubated with GSH (1 mM) for additional 2 h. (B) Average fluorescence intensities for panels (1–6). Reproduced with permission from ref (81). Copyright 2021 American Chemical Society.

Once again, ROS and inflammatory states are a key element, and HOCl levels in neurons are closely associated with the pathogenesis of PD. In 2021, Chen and colleagues developed probe **28** for detecting HOCl in this context,^81^ utilizing a phenothiazine ring system as the HOCl recognition group in combination with a non-HOCl selective ROS-reactive *o*-aminophenyl pyrilium group. Probe **28** is nonfluorescent, becoming a sky-blue fluorescent compound on oxidation of both the pyrilium and phenothiazine motifs to a lactone and sulfoxide, respectively. Probe **28** exhibited rapid response (within 15 s), significant fluorescence enhancement (538-fold) and excellent sensitivity (LOD = 25.8 nM) for HOCl (Figure 12). Probe **28** was successfully employed to visualize HOCl in the brain of a mouse model; successfully differentiating between PD brain tissue and normal controls. Moreover, probe **28** can also be employed to explore the pathogenesis of HOCl-related diseases.

**Figure 13:**
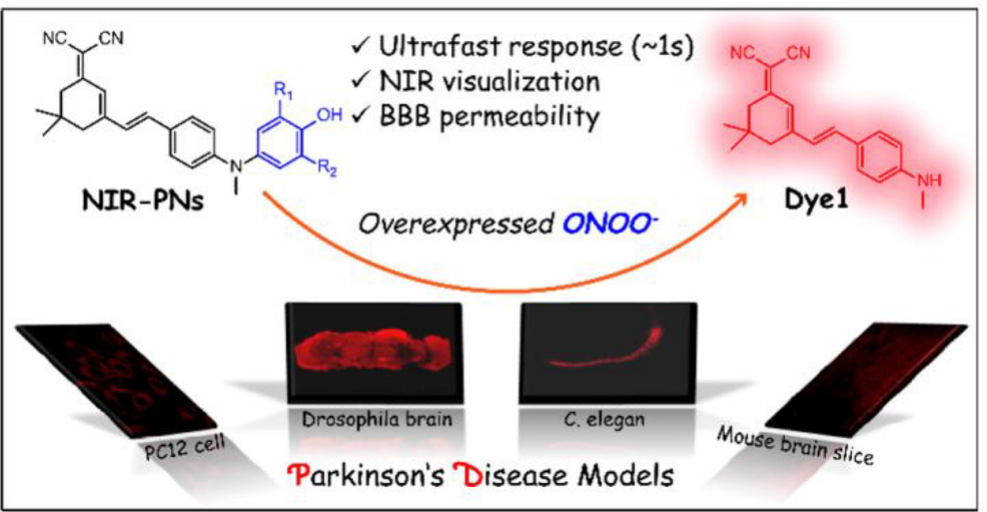
Schematic application of **29** for detecting ONOO^–^ in PD models. Reproduced with permission from ref (82). Copyright 2020 American Chemical Society.

ONOO^–^ is also linked to PD, through its role in oxidative stress, and so its detection is also being considered for PD diagnosis and study. For example, in 2020 Liu et al. designed a series of NIR probes (NIR-PNs) to detect ONOO^–^ in PD models (Figure 13, probes **29**).^82^ Their probes are based around a fairly typical *D*–π–*A* structural design, containing a dicyanoisophorone fluorophore and a *p*-aminophenol receptor with variable substitution as the ONOO^–^ reactive site. Probe **29** boasts a rapid response time (<5 s) and high selectivity for ONOO^–^ detection, and as such has been successfully used to image ONOO^–^ in various PD models, including PC12 cells, drosophila, nematodes, and mouse brains, improving our understanding of the biological role of ONOO^–^ in Parkinson’s disease.

**Figure 14:**
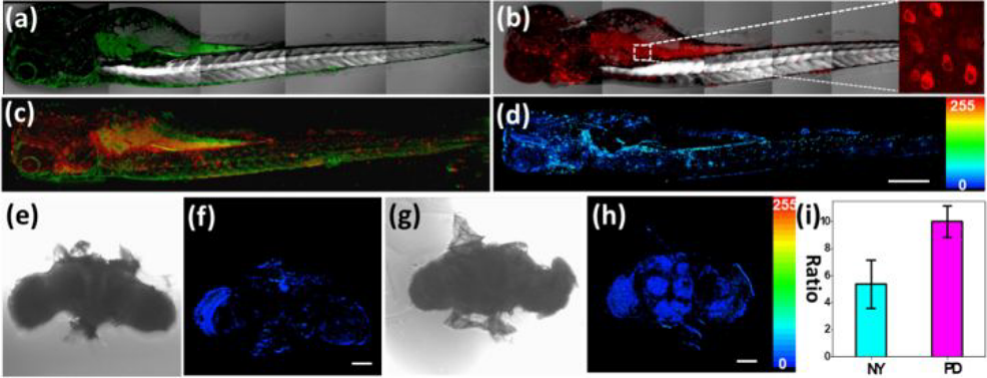
Use of probe **30**: (a) merged imaging DIC channel and green channel with PMT range: 470–520 nm. (b) Merged imaging DIC channel and red channel with PMT range: 620–670 nm, insert was zoom in imaging in zebrafish body. (c) Merged imaging of green and red channel. (d) Ratiometric image generated from (b/a). (e) DIC of wild-type (WT) *Drosophila* brain. (f) The ratio of red/green channel for WT and (g) DIC of PD *Drosophila* brain. (h) Red/green channel ratio for PD. Reproduced with permission from ref (83). Copyright 2018 Elsevier BV.

H_2_O_2_ is yet another ROS that can be studied in PD, using for instance fluorescent probe **30**, developed by Li et al. in 2019.^83^ This probe again uses a boronate ester group as the H_2_O_2_ recognition group. The benzyl BPin motif masks the electron-donating phenol functionality, exhibiting green fluorescence, which inhibits ESIPT (excited state intramolecular proton transfer)-induced NIR emission. When the probe reacts with H_2_O_2_, the boronate ester is oxidized and removed, and the phenol group is restored, which results in the ESIPT-induced NIR fluorescence of the probe. Probe **30** is highly sensitive (LOD = 0.27 μM) and has high selectivity for achieving ratiometric H_2_O_2_ imaging in PD models, including in living cells, zebrafish, and fruit flies, showing great potential for contributing to PD research (Figure 14).

In 2023, to investigate the correlation between formaldehyde and PD *in vivo*, Lin et al.^84^ developed lysosomal-targeted NIR fluorescent probe **31**. The authors used rhodamine derivatives as fluorophores, morpholine groups for lysosomal targeting, and ethylenediamine as formaldehyde recognition groups. Probe **31** has excellent properties such as NIR emission and high selectivity (LOD = 0.48 μM) and could therefore be employed to demonstrate higher levels of FA in PD model cells, zebrafish, and mice. This study suggests that FA could be a potential marker of PD, providing a crucial pathway for the investigation of the pathology of PD and related diseases.

In 2022, Yin et al. reported a dual-response fluorescent probe **32** to detect viscosity and H_2_S in mitochondria.^85^ The NIR-emissive (λ_ex_ = 740 nm) probe consists of a benzoindole salt conjugated to *N*,*N*-dimethyl-4(thiophen-2-yl)-aniline, with this latter group acting as a molecular rotor that responds to changes in viscosity. The C=C linker is sensitive to H_2_S, reaction with which induces an increase in blue emission. Colocalization experiments demonstrated the probe’s excellent mitochondrial targeting ability with a Pearson colocalization coefficient of 0.90. Moreover, the probe can image viscosity changes in a Parkinson’s disease model (PC12 cells treated with glutamate), providing a valuable tool for studying the pathogenesis of PD.

Another H_2_S-targeting probe, probe **33**, has been developed by Kim et al. in 2013.^86^ With probe **33**, a 2-napththyl benzothiazole acts as the fluorophore, a *p*-azide phenyl carbamate acts as the reducible hydrogen sulfide reaction site, and triphenylphosphonium acts as the mitochondrial targeting group. Reduction of the azide leads to cleavage of the carbamate to release an amine, shifting the emission maximum. Using probe **33**, the authors found reduced levels of H_2_S in PD models, correlated to reduced levels of cystathionine β-synthase (CBS), a key enzyme that catalyzes H_2_S production. In studies involving the PD gene DJ-1, hydrogen sulfide production and CBS expression were both reduced in DJ-1 knockout astrocytes and brain sections compared to wild-type controls, indicating that reduced levels of hydrogen sulfide in astrocytes may contribute to the development of PD.

Finally, fluorescent probes have also been developed for imaging methionine sulfoxide reductase (Msrs), an enzyme that is known to catalyze methionine sulfoxide (MetSO) reduction to methionine by taking reducing equivalents from the thioredoxin system, thus helping to protect cells from oxidative damage, whose role in PD we have already seen. Fang et al. developed a small molecule fluorescent probe **34** to detect Msrs in 2017, which is readily reduced by Msrs in the same manner as MetSO.^87^ Probe **34** was used to demonstrate impaired Msrs activity in PD model cells, which suggests that Msrs could be associated with neurodegenerative diseases.

### 2.6 Fluorescent Probes for Stroke

**Figure 15:**
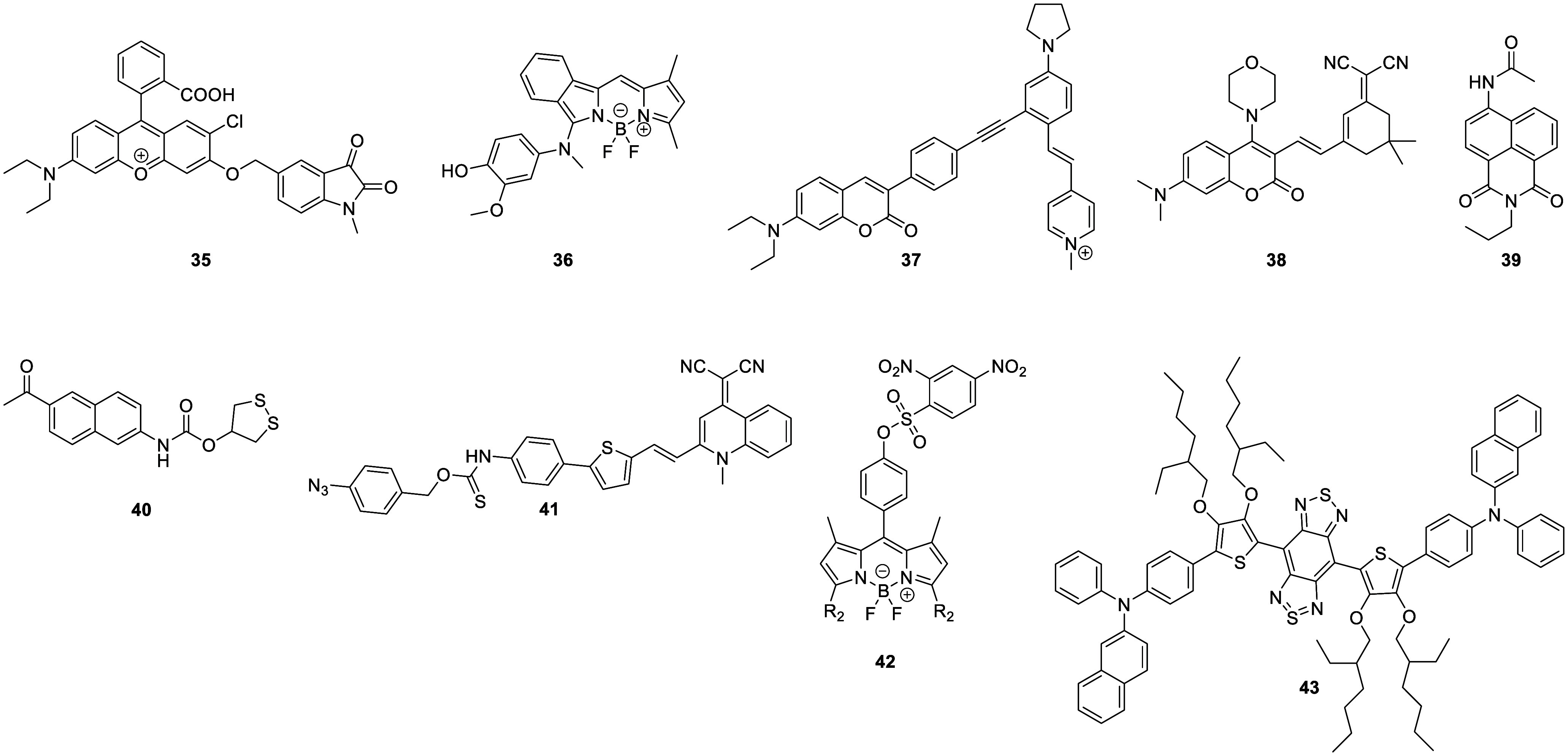
Selected fluorescent probes for stroke.

A stroke, also known as a cerebrovascular accident (CVA), is a medical emergency wherein blood flow to a part of the brain is interrupted or severely reduced, resulting in ischemic damage to brain tissue.^88^ Symptoms of a stroke include sudden numbness or weakness in the face, arms or legs, difficulty speaking or understanding speech, vision problems, dizziness, and severe headaches, with serious long-term impacts caused by irreversible brain damage. With stroke being so severe, many methods of early diagnosis and treatments are extensively being explored (Figure 15).

Once again, ROS and RNS play a key role in the pathology of this condition, with their levels in the blood and blood vessel tissues closely linked to stroke.^89^ To detect ONOO^–^, the two-photon fluorescent probe **35** was proposed by Liu et al. in 2020.^90^ A rhodol derivative was used as the fluorophore, and 1-methylindoline-2,3-dione as the ONOO^–^-specific reactive site. Probe **35** could successfully track endogenous ONOO^–^ in living cells and zebrafish and could be used for real-time observation of ONOO^–^ in cerebral microvessels of ischemic and hemorrhagic stroke rats using a TP microscope. Use of probe **35** overcomes the long-standing challenge of distinguishing ONOO^–^ from other ROS/RNS using optical probes and should enable the effective evaluation of ONOO^–^ related physiological and pathological events, including stroke.

Probe **36** has also been developed for imaging ONOO^–^ in stroke models, as reported by Li in 2019.^91^ In this system, *p*-hydroxyaniline was selected as the reactive trigger for ONOO^–^ sensing and α-chloro benzo-BODIPY was chosen as the fluorophore. Probe **36** can readily cross the BBB, track ONOO^–^ in microvessels, and image ischemia-induced brain injury. This enables it to image overproduction of ONOO^–^ both during thrombus formation and in the brain during early ischemia, proving it a promising tool for investigating the molecular role of ONOO^–^ in the progression of neurovascular injury in stroke.

In 2022, James et al. designed a unique TP ratiometric fluorescent probe **37**, intended for real-time monitoring of autophagy and oxidative stress during oxygen–glucose deprivation/reoxygenation (OGD/R).^92^ This probe is built around a coumarin TP energy donor, which is attached to the receptor moiety via an alkyne bond. This alkyne unit can be readily oxidized to the corresponding aldehyde by ONOO^–^, causing significant changes in the molecule’s conjugated structure and intramolecular charge transfer, thereby impacting its absorption and fluorescence emission. Probe **37** enabled the ratiometric analysis and visualization of autophagy and oxidative stress during OGD/R in real time. The findings suggested that ONOO^–^ is generated during cellular OGD/R, leading to cellular oxidative stress, which is followed by autophagy signals approximately 15 min later. The outcome of this research has tremendous potential to advance the development of new, cohesive systems for the diagnosis, treatment, and drug design of stroke.

**Figure 16:**
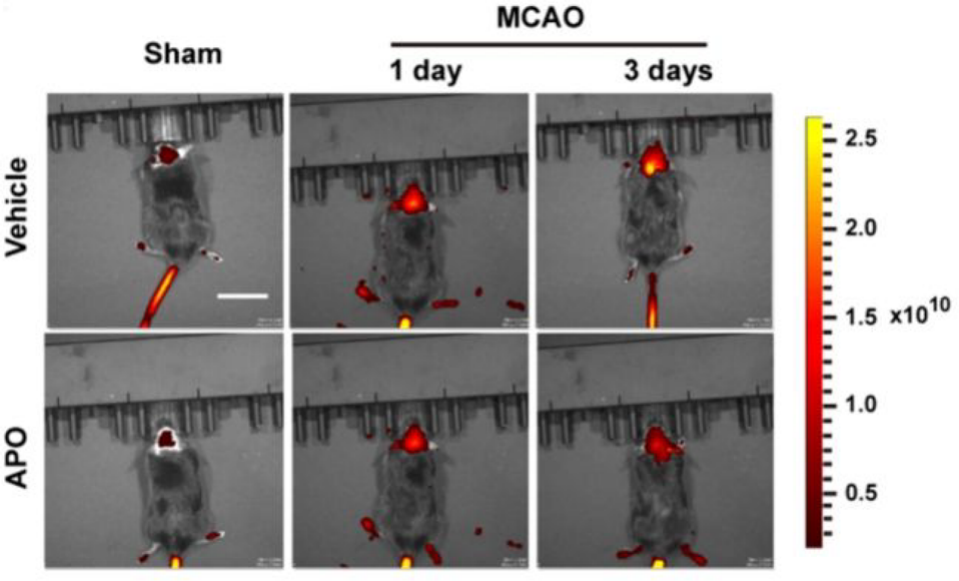
*In vivo* imaging using probe **38** of autophagy in the brain during middle cerebral artery occlusion (MCAO) at different times when subjected to different treatments: Sham group (mice not undergoing MCAO), MCAO group (mice undergoing MCAO), vehicle group (injection of saline to mice tail veins), and APO group (intraperitoneally injection with apocynin in mice). Reproduced with permission from ref (93). Copyright 2022 American Chemical Society.

In 2022, Li et al. investigated probes for the imaging of autophagy, producing the lysosome-targeted fluorescence probe **38**.^93^ With a coumarin derivative as the TP fluorophores, and vinyl-coupled isophorone derivatives as the viscosity sensing units, the morpholine derivatives can locate in lysosomes to accurately detect lysosome viscosity and ultimately enable *in situ* detection and assessment of autophagy levels. The results indicated that autophagy levels increased significantly during stroke, and inhibition of oxidative stress significantly reduced the degree of autophagy (Figure 16). This study confirmed that stroke-induced oxidative stress can lead to the development of autophagy.

In 2016, Wang et al. developed the “off–on” sensitive and selective fluorescent probe **39** for detecting Fe^2+^, which is believed to play an important role in ischemic stroke-related oxidative stress.^94^ This probe uses a naphthalimide fluorophore and a reducible *O*-acyl hydroxylamine as the Fe^2+^ recognition unit. Probe **39** was used to monitor Zn^2+^-induced release of Fe^2+^ in brain cells and detected elevated levels of Fe^2+^ in ischemic brain tissue.

Because stroke is associated with oxidative stress in the brain, and thioredoxin reductase (TrxR) is critical in the regulation of cellular redox homeostasis, probes have been developed for its monitoring in stroke models. One such probe is the TP fluorescence probe **40**,^95^ which was designed using a combination of a 1,2-dithiolane moiety and the 2-acetyl-6-aminonaphthalene fluorophore with a carbamate linker. This system was used to monitor the distribution of TrxR in zebrafish by TP fluorescence imaging and was also able to show a decline in TrxR function within the brains of mice after cerebral ischemia reperfusion injury, suggesting that TrxR is a potential therapeutic target for stroke.

A H_2_S-triggered and H_2_S-releasing near-infrared fluorescent probe **41** was developed for the high-fidelity *in situ* imaging of ferroptosis by James et al. in 2022.^96^ Azobenzene was used for hydrogen sulfide recognition, attached to the quinoline acetonitrile fluorophore by a thiocarbamate (H_2_S precursor). The ability of the linker to rotate means molecular rotation, and hence viscosity, regulates the fluorescence output. Hence, probe **41** could be used to image ferroptosis specifically in high-viscosity environments, with both high sensitivity (LOD = 1.3 nM) and selectivity. While cell experiments indicated that the progression of erastin-induced ferroptosis in cells with and without probe **41** were not significantly different, use of a hydrogen sulfide trigger and a hydrogen sulfide release mechanism during imaging allowed the probe to avoid triggering ferroptosis itself, leading to more accurate results.

In 2022, Gu et al. synthesized the fluorescent probe **42** for detecting glutathione (GSH).^97^ This probe was built around a BODIPY fluorescent scaffold, with a 2,4-nitrobenzenesulfonic acid GSH recognition group at the 3-position. To evaluate the probe, the authors constructed OGD/R and MCAO models to simulate stroke and demonstrated high spatiotemporal specificity for both *in vivo* and *in vitro* fluorescence imaging of GSH during cerebral ischemia–reperfusion (I/R), specifically highlighting disturbances in redox homeostasis during reperfusion. This method provides a new avenue for studying cerebral I/R and should serve as a highly sensitive imaging platform for clinical applications such as postoperative organ diagnosis. This approach may also be extended to other pathological physiological processes involving cellular deoxygenation and reoxygenation.

In 2022, Hong et al. developed the NIR-II (1000–1700 nm, second near-infrared window) fluorescent probe **43** based on the benzo-bis(1,2,5-thiadiazole) (BBTD) structure.^98^ With this probe, BBTD was used as the electron acceptor, and the 3,4-bis(alkyloxy) thiophene ring and *N*,*N*-diphenylnaphthalen-2-amine (BPN) were employed as the electron donors. The 3,4-bis(2-ethylhexyloxy) chain on the thiophene units act as good donors and increase the dihedral angle between BBTD and the thiophenes (up to 52°), thus improving its AIE property (*I*/*I*_0_ > 13). Probe **43** exhibits strong AIE characteristics and a fluorescence quantum yield of 14.45% in the NIR-II region. Hong’s work demonstrated that probe **43** could be used as an effective imaging agent in the image-guided pharmacotherapy of ischemic stroke. Additionally, they were able to use probe **43** to demonstrate that Dengzhan Xixin injection can play a protective role in the ischemic brain by promoting angiogenesis.

### 2.7 Fluorescent Probes for Glioma

Glioma is a type of primary brain tumor that arises from the glial cells in the brain or spine,^99^ common symptoms of which include headaches, seizures, changes in vision or hearing, and difficulty with memory and concentration. The current clinical treatment of a combination of surgery, radiation, and chemotherapy leaves significant room for improvement, and as a result, many researchers are developing surgical navigation methods, early diagnosis techniques, and better treatment modalities using fluorescence imaging.

**Figure 17:**
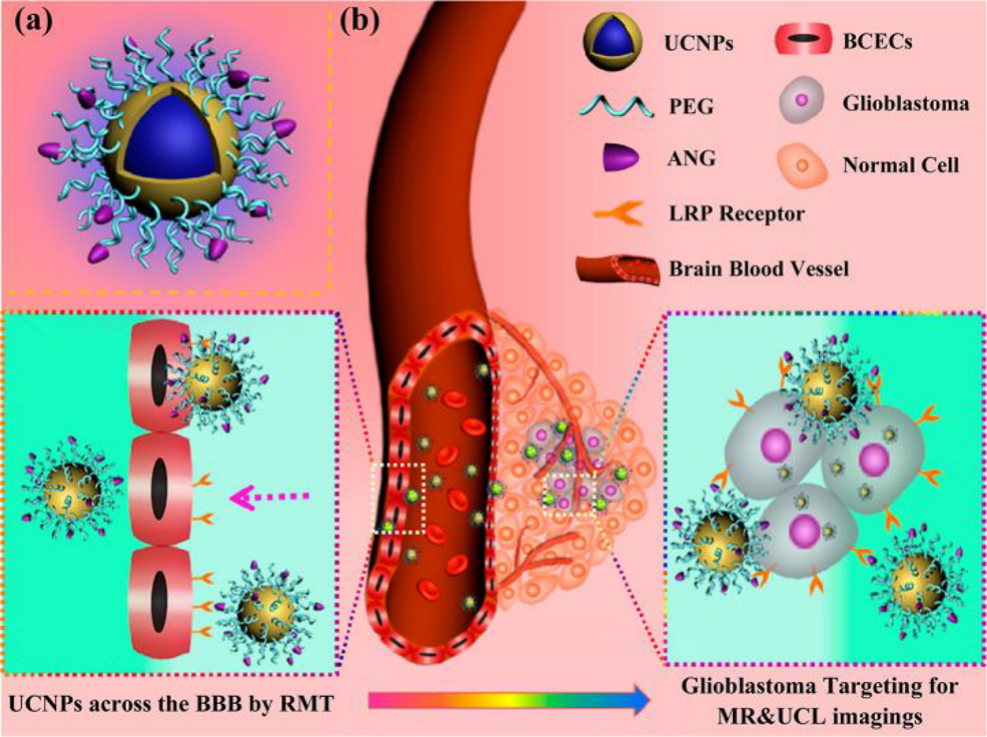
(a) Design of dual-targeting probe **44**. (b) Schematic diagram of probe **44** as the dual targeting system to cross the BBB and target the glioblastoma via LRP mediated endocytosis, enabling MR and UCL imaging of intracranial glioblastoma. Reproduced with permission from ref (100). Copyright 2014 American Chemical Society.

Multifunctional materials are of interest here, as they have the advantages of performing multiple functions unlike the types of small molecule probes that have been discussed so far within this review. For instance, in 2014, Shi et al. developed a brain nanoprobe (ANG/PEG-UCNPs, Figure 17, probe **44**), which can cross the BBB and target glioblastoma (GBM, a high-grade fast-growing glioma).^100^ Probe **44** is composed of Gd(III) anchored to polyethylene glycol (PEG)-based up-conversion nanoparticles (UCNPs) and dual-targeting ligand Angiopep-2 (ANG, TFFYGGSRGKRNNFKTEEY), which can specifically bind to low density lipoprotein receptor-related proteins, which is overexpressed in BBB and GBM cells. Results from both cell and animal experiments showed that probe **44** can effectively target GBM by crossing the BBB through receptor-mediated endocytosis. These bimodal nanoprobes have great potential for preoperative diagnosis and localization of brain tumors using noninvasive fluorescence imaging, with superior imaging performance compared to clinically used magnetic resonance (MR). As a proof-of-concept, this shows great potential for diagnosis and fluorescence localization of glioblastoma, which could lead to efficient tumor surgery.

**Figure 18:**
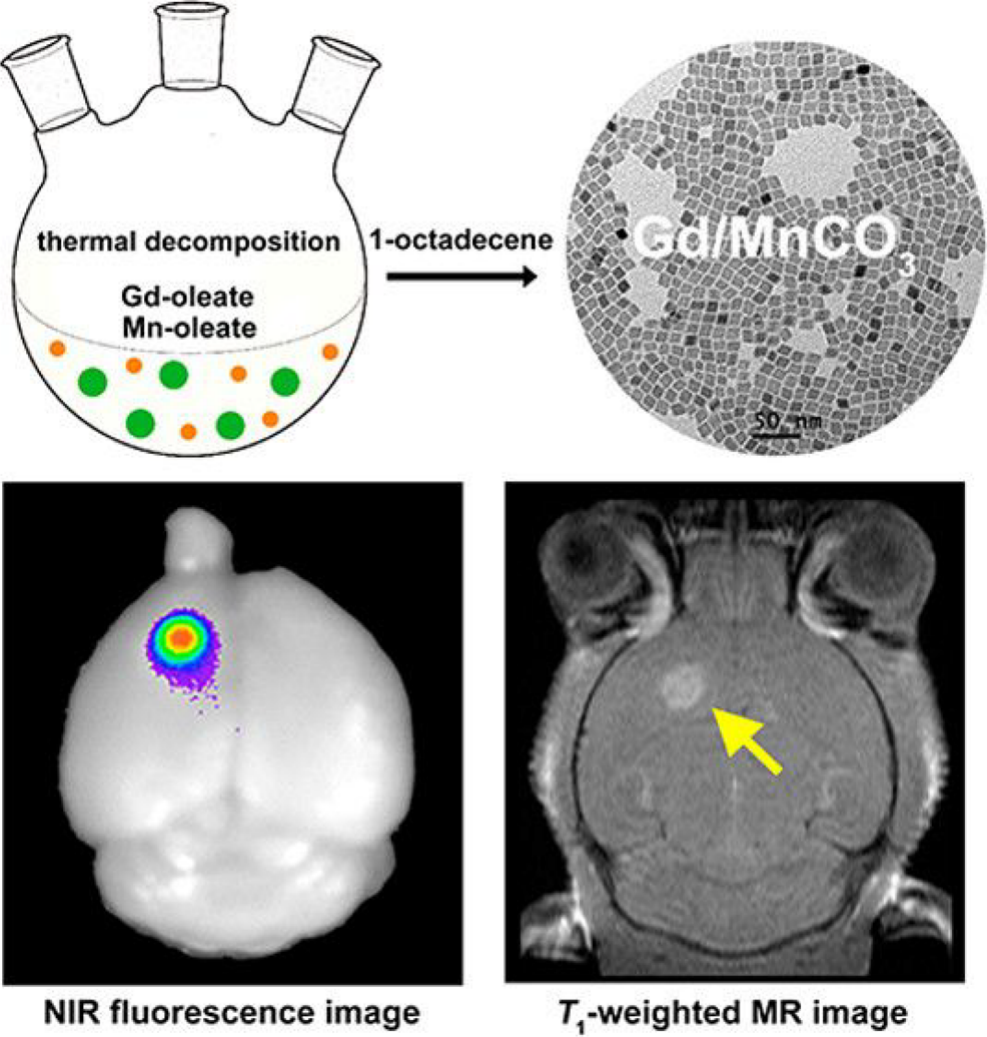
Schematic Illustration of the synthesis of multifunctional probe **45** and NIR fluorescence image and MR image. Reproduced with permission from ref (101). Copyright 2015 American Chemical Society.

In 2015, Ye et al. reported 1 nm sized Gd-doped MnCO_3_ nanoparticles for targeted MR and fluorescence imaging of microscopic brain gliomas by thermal decomposition in the presence of manganese-oleate (probe **45**).^101^ The authors doped Gd(III) into MnCO_3_ based nanoparticles and engineered high water dispersion and excellent water stability through carboxylate-terminated silane ligand exchange and PEG conjugation. Probe **45** could be used to create multifunctional nanoprobes by combining the NIR dye cyanine 5.5 with the targeting ligand folic acid (FA). This nanoprobe combines the high spatial resolution of MRI with the high sensitivity of fluorescence imaging (Figure 18), making them effective in detecting early glioma.

In 2021, Pilar et al. developed dual MRI and fluorescence imaging conjugated polymer nanoparticle (CPN) as a nanoprobe for GBM detection (probe **46**).^102^ These conjugated polymer nanoparticles were synthesized by nanoprecipitation to incorporate a metal oxide magnetic core (Fe_3_O_4_/ NiFe_2_O_4_ nanoparticles) into the matrix capped with oleic acid. The obtained CPNs had good biocompatibility and cell penetration. After intravenous administration, associated CPNs **46** were detected in tumors and excretory organs of ectopic GBM models and could also be used to image GBM in live models, providing a novel approach for the development of multimodal imaging probes.

**Figure 19:**
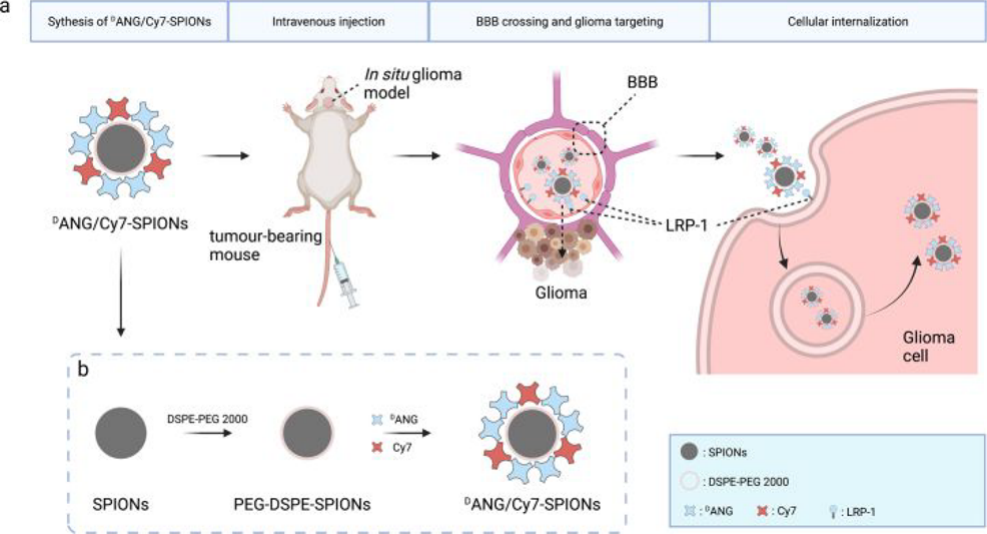
(a) Schematic illustration of the construction and function of probe **47**, including their mechanism of crossing the BBB and targeting glioma cells and (b) their synthesis process. Reproduced with permission from ref (103). Copyright 2021 Springer Nature.

In 2021, Gong et al. reported dual-modality imaging nanoprobes using a combined MRI/NIR fluorescent technology to locate malignant gliomas *in vivo*.^103^ These nanoprobes utilize entosis-triggering ligand Angiopep-2 (ANG), to recognize low-density lipoprotein receptor protein 1, which is overexpressed on brain capillary endothelial cells and glioma cells. This enables ANG to cross the BBB and directly target glioma cells. By combining superparamagnetic iron oxide nanoparticles (SPIONs) with the NIR fluorescent dye indocyanine (cyanine 7, Cy7) and ANG, Gong constructed a dual-modal imaging probe that can image GBM (Figure 19, probe **47**). Probe **47** can mediate the precise aggregation and detection of glioma sites by nanoprobes using both MRI and NIR fluorescence imaging. This has great potential for preoperative diagnosis and intraoperative localization, making probe **47** a promising glioma targeting contrast agent.

**Figure 20:**
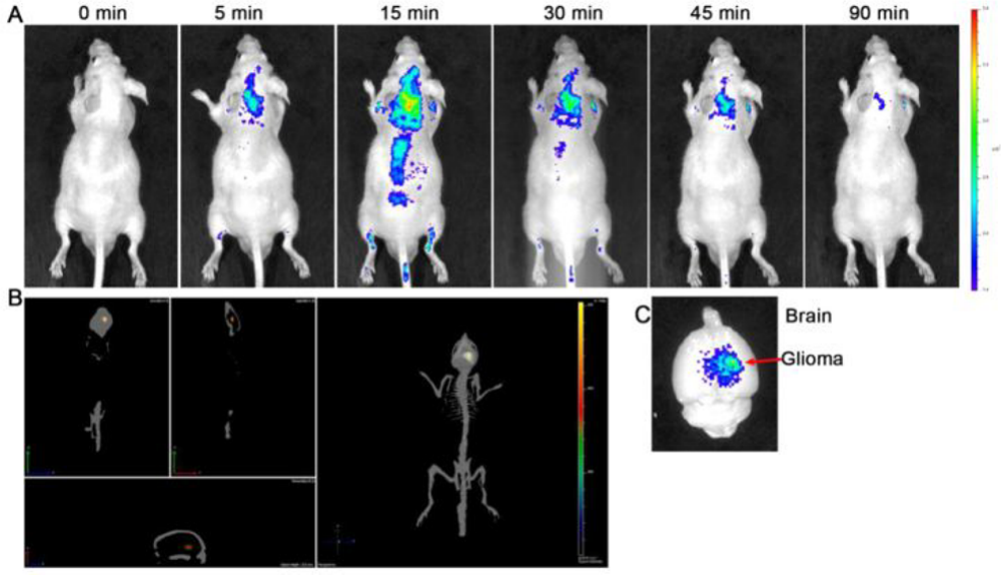
*In vivo* and *ex vivo* imaging of glioma-bearing mice after tail intravenous injection of probe **48**. (A) Whole body distribution of probe **48** as a function of time after injection. (B) Three-dimensional reconstruction of probe **48** distribution in the brain 20 min after injection. (C) *Ex vivo* imaging of the brain 90 min after the injection of probe **48**. Reproduced with permission from ref (106). Copyright 2015 American Chemical Society.

Fluorescent carbon dots (CD) have the advantages of low toxicity, high stability, versatility, and biodegradability, which make them suitable for use as fluorescent sensing and as imaging materials.^104,105^ In 2015, Sun et al. developed glioma-targeting carbon dots (CD-Asp) using d-glucose and l-aspartic acid as starting materials (probe **48**).^106^ Probe **48** has high biocompatibility and can target glioma cells without the need for additional targeting molecules. *In vivo* imaging studies confirmed that probe **48** localizes at glioma sites at a much higher rate than for normal brain tissue, indicating that probe **48** can be used as a targeting fluorescence imaging agent for brain gliomas (Figure 20). This work demonstrates that probe **48** can serve as a platform for building smart nanomedicines that integrates diagnostic, targeting, and therapeutic capabilities.

Using a similar CD-based platform in 2015, Gao et al. reported a bioimaging probe (RGD-PEG-CDs) for U87 glioma (probe **49**).^107^ The RGD ligand targets receptor αvβ3, which is highly expressed on most tumor and neovascular cells, and is attached to CDs after PEGylation. *In vivo*, probe **49** can actively target U87 gliomas, and the fluorescence distribution in tumor sections indicated that probe **49** could also target neovascularization, as expected based on αvβ3 expression.

Another system is that reported by Yuan et al. in 2022, who developed a biomimetic nanoprobe Pdots-C6 for targeted detection of glioma (probe **50**).^108^ The authors selected triphenylamine (TPA)-functionalized PTZ as the electron donor and benzothiazole (BBT) as the electron acceptor to induce ICT within the PTZTPA-BBT polymer backbone, producing the desired probe Pdots with long-wavelength optical activity. They then coated the Pdots with C6 glioma cell membranes, thereby enhancing the biocompatibility and homologous targeting ability of probe **50**, significantly improving their NIR-II glioma-imaging capability compared to naked Pdots. This work provides interesting innovations and examples for the development of a biomimetic nanoplatform for accurate glioma diagnosis.

In 2017 Cheng and colleagues designed a nanoprobe (QD-Apt) for targeted tumor detection by combining the advantages of PEG-quantum dots (QDs) with aptamers (probe **51**).^109^ A32 is a single-stranded DNA (ssDNA) that binds to epidermal growth factor receptor variant III (EGFRvIII), widely distributed on the surface of glioma cells. With this in mind, Cheng et al. developed a nanoprobe biotin-aptamer-conjugated streptavidin-PEG-CdSe/ZnS QDs (QD-Apt) by coupling A32 to the surface of QDs to enable specific binding to tumors. Probe **51** exhibits a strong fluorescent signal both *in vivo* and *ex vivo*, specifically binding to EGFRvIII. Additionally, probe **51** can visualize the tumor borders of U87-EGFRvIII glioma *in situ* in brain tumor mice, helping surgeons maximize glioma resection. Further development of probe **51**, or systems derived from it, could help provide a promising tool for molecular diagnosis, image-guided surgery, and postoperative examination of gliomas.

**Figure 21:**
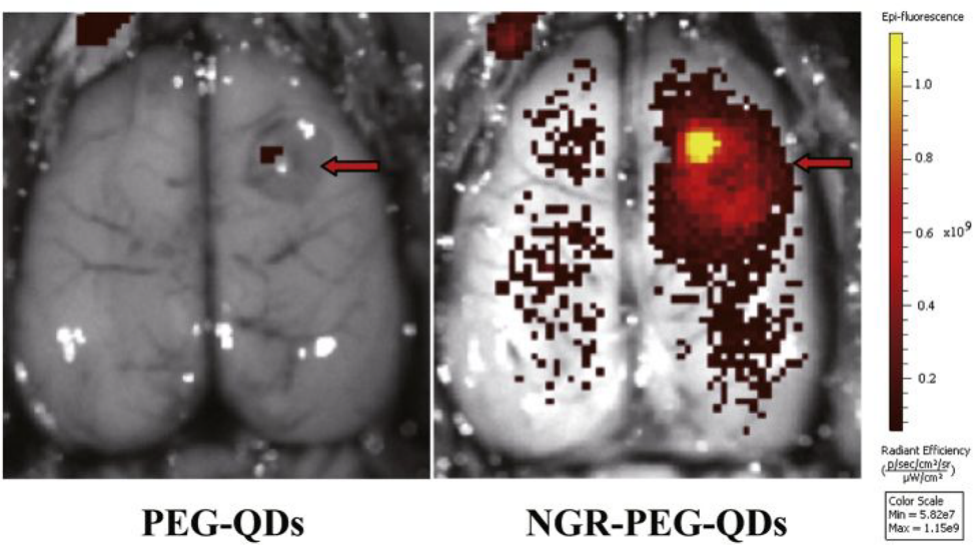
Fluorescent imaging of tumor in rat brains 8 h after tail vein injection of PEG-QDs or NGR-PEG-QDs (probe **52**). Reproduced with permission from ref (110). Copyright 2016 Elsevier.

In 2017, Cheng’s group synthesized another nanoprobe (NGR-PEG-QDs) for targeted detection of gliomas and tumor vasculature (probe **52**).^110^ These nanoprobes were designed to target alanine aminopeptidase CD13, found only in tumor vessels. This was achieved by conjugating biotinylated asparagine-glycine-arginine (NGR) peptide that recognizes CD13 to avidin-PEG-coated QDs. Probe **52** can cross the BBB and image glioma and tumor vessels, and operates at low nontoxic concentrations as shown in Figure 21, possibly facilitating a move toward clinical nanomedicine.

Another example is that by Xu et al., who synthesized the nanofluorescent probe AsT (probe **53**) in 2014.^111^ The peptide TGN was used for targeted BBB delivery in combination with AS1411, a guanine-rich aptamer that mediates nanoparticle directing to gliomas by interacting with overexpressed nucleolin. TGN and AS1411 were coupled via a PEG linker. A cyanine 3 fluorescent tag was appended to the end of AS1411 to track the AsT nanoprobe. *In vitro* cellular uptake and glioma spheroid uptake indicated that probe **53** could be taken up by both glioma and endothelial cells, penetrate the endothelial cell monolayer, and was taken up by glioma spheroids. *In vivo* experiments confirmed that probe **53** could effectively target gliomas with high intensity.

**Figure 22:**
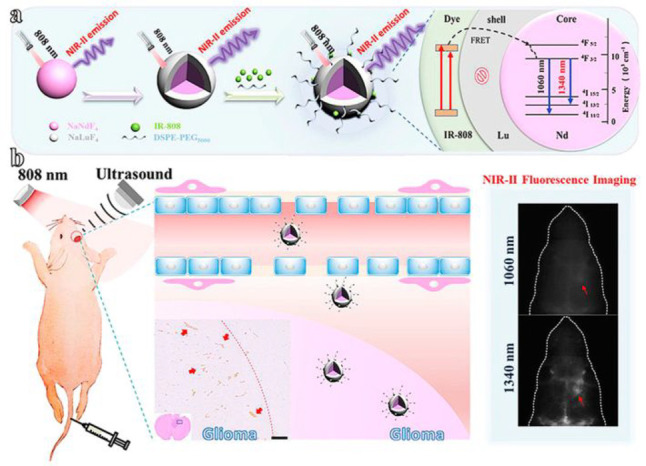
(a) Schematic illustration of the synthesis of water-soluble dye-sensitized core–shell NaNdF_4_@NaLuF_4_/IR-808@DSPE-PEG5000 NPs (probe **54**) and their energy transfer mechanism. (b) Application of these core–shell NPs in NIR-II fluorescence imaging of orthotopic glioblastoma under ultrasound-mediated opening of the BBB, and rare-earth staining of brain tissue after delivery into the brain. Reproduced with permission from ref (112). Copyright 2019 Elsevier.

In 2019, Li and colleagues synthesized nanoparticles based on NaNdF_4_ with strong NIR-II fluorescence for detecting orthotopic glioblastomas (Figure 22, probe **54**).^112^ They coated the NaNdF_4_ nanoparticles with an inert layer of NaLuF_4_ and sensitized them with a near-infrared dye (IR-808), resulting in a 10-fold increase in their conventional emission. The researchers used focused ultrasound to efficiently deliver these nanoparticles to tumor tissue, which temporarily opened the BBB in mice. Fluorescence imaging and the use of rare-earth staining of brain tissue confirmed that the nanoparticles targeted tumors specifically, thus demonstrating the applicability of dye-sensitized rare earth nanoparticles for potential diagnosis of glioblastoma. This should provide a roadmap for enhancing neglected NIR-II imaging with weak long-wavelength fluorescence.

**Figure 23:**
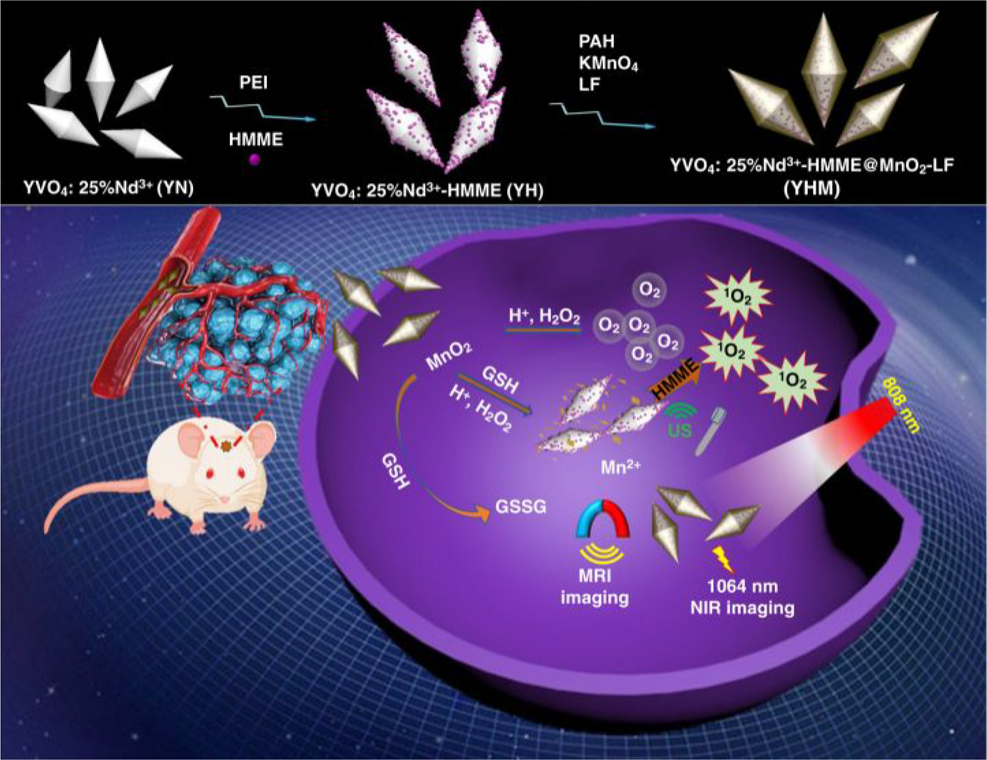
Schematic diagram of the assembly and mode of action of YHM nanotherapeutics. Reproduced with permission from ref (113). Copyright 2022 Springer Nature.

Zhang et al. developed a nanotherapeutic agent marketed as YHM in 2022, which can be used for the diagnosis and treatment of glioma *in situ* (Figure 23, probe **55**).^113^ In YHM, low-energy phonons yttrium vanadate (YVO_4_) and Nd^3+^ particles were used as the core, ultrasound sensitizer hematoporphyrin methyl ether was the carrier, and MnO_2_ nanosheets were the *in situ* glioma NIR-II/MRI imaging and high-efficiency sonodynamic therapy (SDT) element. Probe **55** was shown to readily cross the BBB and be competent for both NIR-II fluorescence and MRI imaging of glioma. In addition, probe **55** enables SDT of gliomas *in situ*, where the MnO_2_ shell not only generates O_2_ but also releases Mn^2+^ ions, combining for an enhanced therapeutic effect of SDT, which paves the way for expanding the application of rare earth ion-doped YVO_4_ luminescent nanoparticles.

## 3 Fluorescent Probes for Cancer

Malignant tumors are a serious threat to human life, with tens of millions of new cases and deaths worldwide each year. These are most commonly caused by breast, lung, or colorectal cancers and can be caused by a host of environmental and genetic factors. According to the World Health Organization (WHO), by 2035, there will be over 20 million new cancer cases and more than 14 million cancer deaths annually.^114,115^ Fortunately, recent societal and technological improvements have drastically improved cancer survival rates, with a 1-in-3 chance of survival if the tumor is detected early. The discovery of markers such as enzymes and small molecule biomarkers of cancer provides an opportunity for the rapid diagnosis of cancer.^10,116−118^

This section will look at such biomarkers and the recent developments of a multitude of tools for their fluorescence imaging (Table 2), focusing primarily on some of the more common forms of cancer (breast, liver, lung, ovarian, cervical), and looking at key and common biomarkers that can be readily monitored using fluorescence imaging.

**Table 2:** Selected Fluorescent Probes for Cancer

probe	λ_ex_ /λ_em_ (nm)	LOD	bioactive molecule	biological model	ref
Breast Cancer					
**56**	365/532		^1^O_2_	MDA-MB-468 cells	(120)
**57**	480/505	0.56 nM	HClO	MCF-7 cells	(121)
**58**	781/800		hydroxyapatite (HA)	rat breast cancer microcalcification	(122)
**59**	646/664		cysteine protease activity	4T1 syngeneic orthotopic mouse breast tumors	(123)
**60**	690/710		GGT and caspase-1	4T1-tumor-bearing Balb/c mice	(124)
**61**	530/600		phosphatidylserine (PtdSer)	MMTV-PyMT breast cancer mice	(125)
**62**	675/710		granzyme B	4T1 tumor-bearing Balb/c mouse	(126)
**63**	465/665		GSH	BCap-37 tumor xenograft mice	(127)
**64**	650/725	1.5 × 10^–5^ U/mL	urokinase-type plasminogen activator (uPA)	MDA-MB-231- and MCF-7- tumor-bearing mice	(128)
**65**			quiescent cancer stem cells (CSCs)	AS-B145-1R cells	(129)
Liver Cancer					
**66**	445/650	0.13 ng/mL	CD13/aminopeptidase N (APN)	Balb/c mice bearing HepG-2 xenograft tumor	(134)
**67**	406/532		phosphatase	HepG2 cells	(135)
**68**	428/540	50 nM	mitochondrial thioredoxin (Trx)	HepG2 cells, HeLa cells	(136)
**69**	438/538		thioredoxin reductase (TrxR)	HepG2 cells	(137)
**70**	450/564	0.02 nmol/mL (CYP1A2) 0.05 nmol/mL (CYP1A1)	cytochrome P450 1A (CYP1A)	rat liver slice, HepG2 cells, A549 cells	(138)
**71**	405/519, 558, 593		N/A	HepG2 cells, LO2 cells, 7721 cells	(139)
Lung Cancer					
**72**	620/680	10.6 nM (HClO), 7.9 nM (ONOO^–^), 0.14 μM (HO·)	hROS: HClO, HO, and ONOO^–^	A549 cells, HeLa cells tumor-bearing mouse xenograft mice	(142)
**73**	769/788		nitroreductase (NTR)	A549 cells, A549 tumor mouse	(143)
**74** and **75**	675/710		pH	A549 tumor mouse	(144)
Ovarian Cancer					
**76**	510/582, 450/556		GGT	OVCAR5 and SKOV-3 cells, HUVEC cells	(147)
**77**	498/518		β-galactosidase	ovarian cancer cells and tumor-bearing mice (SHIN3, SKOV3, OVK18, OVCAR3, OVCAR4, OVCAR5 and OVCAR8)	(148)
**78**	300/546, 616		lysophosphatidic acid (LPA)		(149)
Cervical Cancer					
**79** and **80**	Probe1:		Hcy, Cys, GSH, SO_2_	HeLa tumor-bearing mice	(151)
	380/480, 625				
	450/545, 625;				
	Probe2:				
	380/465, 635;				
	380/450, 635				
	450/540, 635;				
	450/535, 635				
**81**	403/557		lysosomal ATP	HeLa cells	(152)
**82**	457/547	0.11 μg/mL	COX-2	HeLa, MCF-7, and HEK293 cells	(153)
**83**			caspase-3/7	HeLa tumor-bearing mice	(154)
**84**	674/694		caspase-3/7	HeLa cells	(155)
Other					
**85**	510/590		ATP	OSCC cells	(156)
**86**	405/540		senescence-associated βgal (SAβgal)	SK-MEL-103 tumor-bearing mice	(157)
**87**	450/500, 685	1.7 × 10^–4^ U mL^–1^	β-galactosidase (β-gal)	LoVo tumor-bearing mice	(158)
**88**	463/555,615		COX-2	tumors in mice (MKN45, BEL7402, MDA-MB-231)	(161)
**89**	783/840		Monoamine oxidase A (MAOA)	C4–2B tumor xenografts in mice	(162)
**90**	620/665		matrix metalloproteinases (MMPs)	HT-1080 tumor-bearing nude mouse	(163)
**91**	490/545		acylprotein thioesterases (APTs)	HEK293T, A431, MDA-MB-231 and MCF-7 cells	(164)
**92**	410/550	1.36 U/L	alkaline phosphatase (ALP)	U-2OS and Saos-2 cells, HeLa and HepG2 cancer cells	(165)
**93**	675/694		matrix metalloproteinase (MMP)	HT1080 tumors mice and BT-20 tumors mice	(166)
**94**	455/500, 650/680	0.74 nM	pH and matrix metalloprotease-9 (MMP-9)	LS180 tumor-bearing mice	(167)

### 3.1 Fluorescent Probes for Breast Cancer

**Figure 24:**
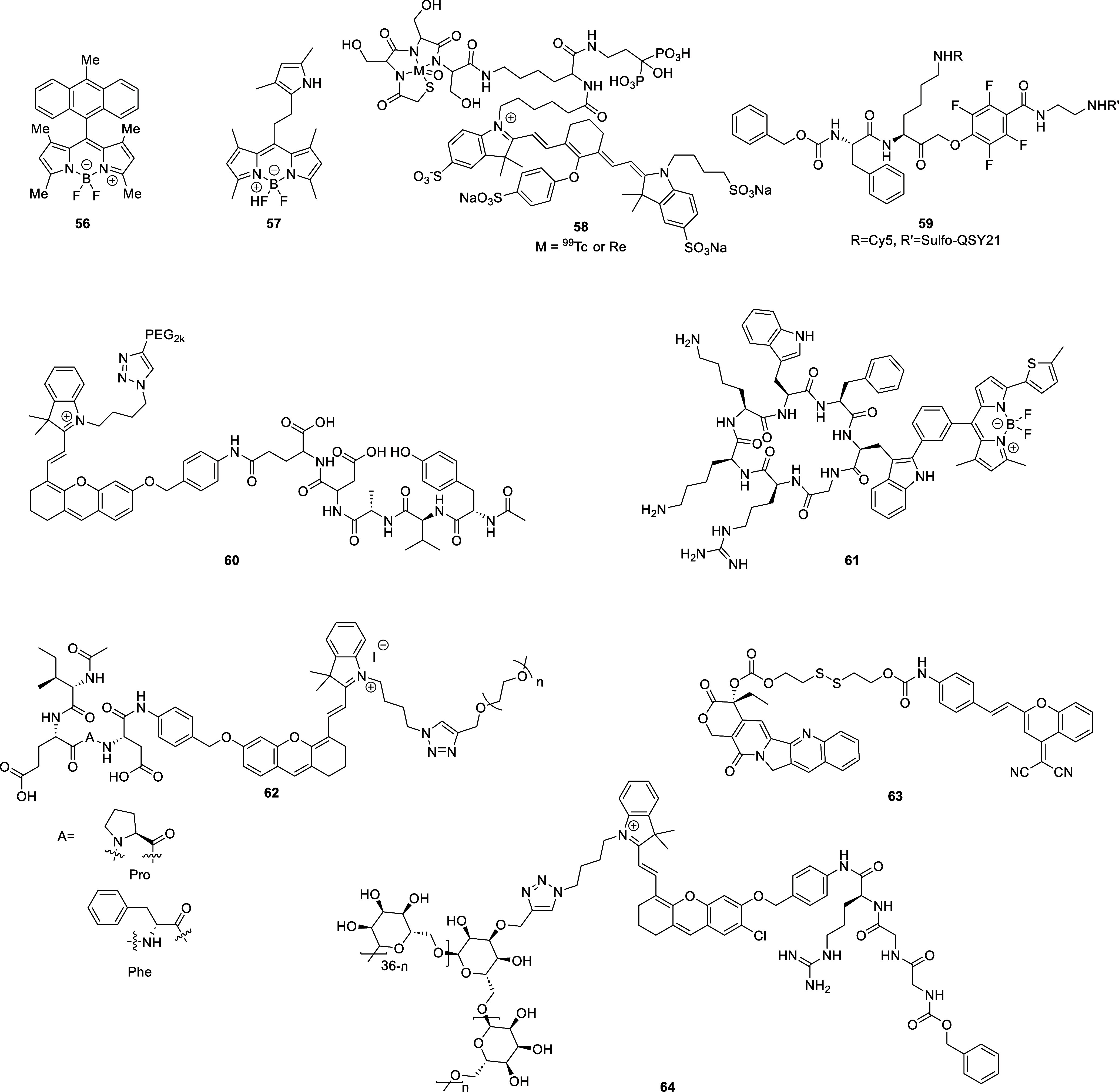
Selected fluorescent probes for breast cancer.

Breast cancer is the most common malignancy worldwide, with on average one in ten women developing breast cancer at some stage in their life.^119^ Although in recent years the death rate has decreased significantly thanks to early diagnosis and effective treatment, further improvement of both is still needed (Figure 24).

As with the neurological diseases discussed in the previous section, ROS (and RNS), and oxidative stress more generally are also a key feature of cancer. Thus, in 2017, Boyle et al. developed fluorescent probe **56** for visualizing singlet oxygen (^1^O_2_) within cells.^120^ Probe **56** was constructed around a BODIPY–anthracene dibody structure, which generates a locally excited triplet state by PeT upon excitation. Molecular oxygen generates ^1^O_2_ by quenching this triplet, which can then react with the anthracene to form anthracene peroxides, and eventually anthracene epoxides, and polycyclic acetals, which emit a bright fluorescence.

HClO is also present in large amounts in cancers. With this knowledge in hand, in 2014, Peng et al. reported **57** as a highly sensitive probe for monitoring the production of HClO in cancer cells.^121^ This probe is based on the use of a BODIPY fluorophore, with a pyrrole group acting as the HClO recognition unit. A strong PeT quenching effect between the two units leads to fluorescence quenching when no analyte is present, with oxidation of the pyrrole on exposure to the strongly oxidizing HClO, interrupting the PeT, thus “turning on” the fluorescence. Probe **57** boasts ultrahigh sensitivity with a detection limit of 0.56 nM and a fast response time (<1 s), making it particularly suitable for monitoring changes in HClO levels in tumor cells. This probe was also used to image time-dependent elevation of HClO in MCF-7 cells induced by elesclomol, providing a valuable tool for the real-time monitoring of HClO concentrations in tumors.

In 2008 Frangioni’s group designed and synthesized a SPECT/NIR dual-mode fluorescent probe **58** that can be used to image hydroxyapatite (HA).^122^ This probe uses cyanine as the fluorophore and bisphosphonates to recognize HA. The design of probe **58** was specific to HA, eliciting a response to HA that was 8 times faster than to other calcium salts. Through fluorescence imaging and SPECT analysis, the authors successfully observed the ectopic expression of bone morphogenetic protein-2 (BMP-2) in breast cancer rat models.

Cysteine cathepsin is a family of proteases involved in normal cell physiology and the development of many human diseases, including breast cancer. In 2013, the Bogyo group reported the design and synthesis of a new class of probes (such as probe **59**) based on quenched fluorescence activity.^123^ These systems contain a highly electrophilic phenoxymethyl ketone electrophilic “warhead”, which reacts with active site nucleophiles of the analyte. Bogyo et al. developed an improved linker to connect the photoactive Cy5 and QSY21, causing highly effective Förster resonance energy transfer (FRET) fluorescence quenching in the absence of the target molecule. When cysteine cathepsin is present, the polypeptide sequence is cleaved, releasing the quencher, and causing a turn-on response. Probe **59** showed improved solubility, *in vivo* properties, and broader reactivity toward a wider spectrum of cysteine cathespins than previous probes, resulting in significantly improved labeling and tumor imaging properties. In live fluorescence imaging experiments, the probe clearly identified the difference in cysteine cathepsin activity between breast cancer mice and normal mice, providing an exciting tool and improved approach for developing this type of quenched fluorescent probe, which has now been widely adopted throughout the sensing community.

Real-time imaging of programmed cell death (PCD) is essential for monitoring the development of cancer, its treatment, resistance mechanisms, and for customizing treatment options, because PCD evasion is a hallmark of cancer. Therefore, in 2023, Pu et al. developed a double-lock tandem activated near-infrared fluorescent probe **60**, for visualizing pyroptosis of mouse tumor cells.^124^ Probe **60** uses Cy5 as its fluorophore and modified PEG to enhance its water solubility. Peptide sequences for γ-glutamyltranspeptidase (GGT) and Casp1 were connected to the fluorophore, yielding probe **60**. On entering the tumor cells, the probe is sequentially cut by Casp1 and GGT enzymes to restore ICT and restore fluorescence at 710 nm. Using probe **60**, the authors observed the level of pyroptosis in breast cancer and evaluated cancer immunotherapy in real time. In addition, the probe can distinguish between intratumor and normal pyroptosis, facilitating the use of optical imaging for evaluating the pyrogenic activity of potential anticancer agents.

Investigating another aspect of PCD, in 2022, Vendrell et al. developed the fluorescent probe **61** for the rapid detection of chemotherapy-induced apoptosis.^125^ The probe uses environment-sensitive BODIPY as the fluorescent unit, and (previously developed) apoptotic peptides as the targeting unit, where the cyclic peptide binds well to apoptotic cell membranes but not to healthy cells. On binding, the surrounding polar environment changes drastically, leading to bright red 600 nm-centered emission from the BODIPY fluorophore. Probe **61** enables the fast identification of apoptotic and healthy cells *in vitro* and *in vivo* with good selectivity. Probes **60** and **61** both provide excellent manifolds for the imaging of cell death, providing excellent tools for differentiating between healthy and apoptotic/pyroptotic cells, and the means to study different PCD mechanisms in cancer and better understand the nature of therapeutic effects.

Real-time imaging of immune activation is also critical for cancer immunotherapy and drug discovery. Unfortunately, most existing probes for this application are designed using fluorophores, whose emission is “always on”, and therefore, the fluorescent response is often poorly correlated to the immune response. Working to remedy this, Pu et al. developed a renal clearance NIR macromolecular fluorescent probe **62** in 2020, to specifically detect granzyme B for use in real-time evaluation of cancer immunotherapy.^126^ Probe **62** contains a cyanine fluorophore and a granzyme B-specific polypeptide, where the fluorescence changes, induced by granzyme B, correlates well with the cell populations of cytotoxic T-lymphocytes (CD8^+^) and T-helper cells (CD4^+^) in tumor tissue. Not only can this probe enter mouse tumor cells by passive targeting (after systemic administration), but it also has excellent renal clearance efficiency; at 60% over 24 h. This probe provides a promising method for monitoring the effects tumor immunotherapies and could help promote the development of immunotherapeutic treatments.

Understanding the biological distribution and *in vivo* activation of prodrugs is essential for drug development, and so in 2014 Zhu et al. designed probe **63** for the study and treatment of breast cancer.^127^ By tethering a dicyanomethyl-4H-pyran-derived NIR fluorophore and known anticancer agent camptothecin (CPT) using a disulfide linker, and attaching the system to nanoparticles, the authors developed an effective theranostic system. First, the high GSH concentration in tumor cells cleaves the linker, which has the effect of both releasing the anticancer agent CPT, and turning on the fluorescence; allowing for the monitoring of drug distribution in real time. The results of *in vivo* experiments confirmed that probe **63** exhibits comparable therapeutic effect to the CPT drug itself, while also enabling the tracking of drug release by NIR fluorescence.

In 2020, Pu and Miao et al. designed the fluorescent probe **64** to distinguish between invasive and noninvasive breast cancer.^128^ Probe **64** specifically responds to overexpressed urokinase-type plasminogen activator (uPA) in invasive breast cancer. After amide bond cleavage to remove the targeting peptide, ICT is activated, triggering NIR fluorescence and PA signal. Probe **64** has a dextran backbone that both improves solubility, but more importantly enables facile renal clearance of the compound, minimizing its potential toxicity. Dextran backbones and their uses in sensing and theranostics will be discussed in detail in the later parts of this review.

**Figure 25:**
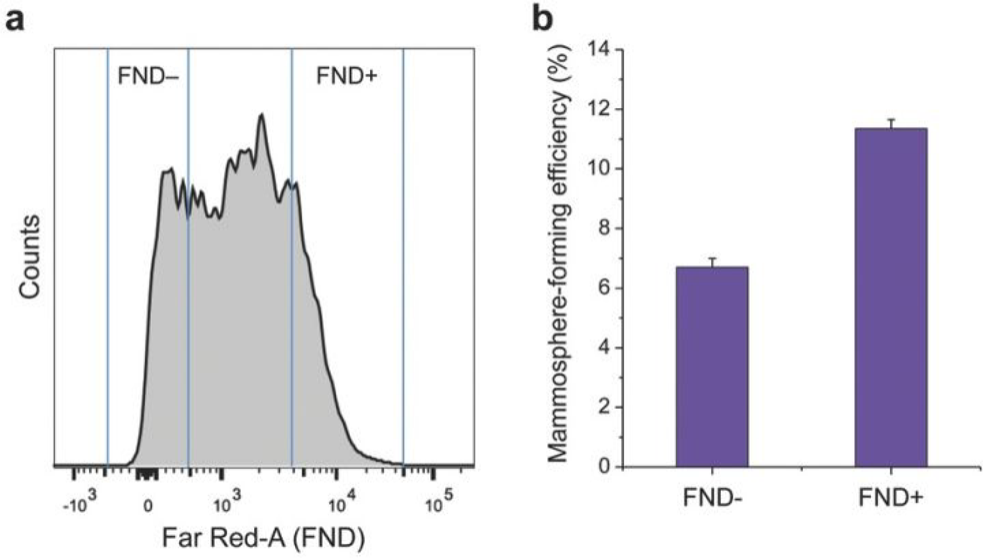
Flow cytometric analysis (a) and mammosphere forming efficiencies (b) of fluorescent nanodiamonds positive (FND^+^) and fluorescent nanodiamonds negative (FND^–^) cells. Reproduced with permission from ref (129). Copyright 2015 John Wiley & Sons.

Although the identification and isolation of cancer stem cells (CSC) would be a great step forward in cancer treatment, this cannot currently be effectively achieved due to a lack of suitable imaging techniques. In 2015, Chang and Yu et al. worked toward solving this issue and developed a fluorescent nanodiamond (FND) material (probe **65**) for tracking and finding slowly proliferating/resting CSCs (Figure 25).^129^ Probe **65** consists of CuInSe_2_/ZnS core/shell quantum dots with highly luminescent properties and tumor-targeting peptides (Cys-Gly-Lys-Arg-Lys, CGKRK). *In vitro* experiments showed that probe **65** had excellent photostability and good biocompatibility, and can be used to quantify the stem cell frequency of a breast cancer cell line, a significant step toward isolation of these crucial stem cells.

### 3.2 Fluorescent Probes for Liver Cancer

**Figure 26:**
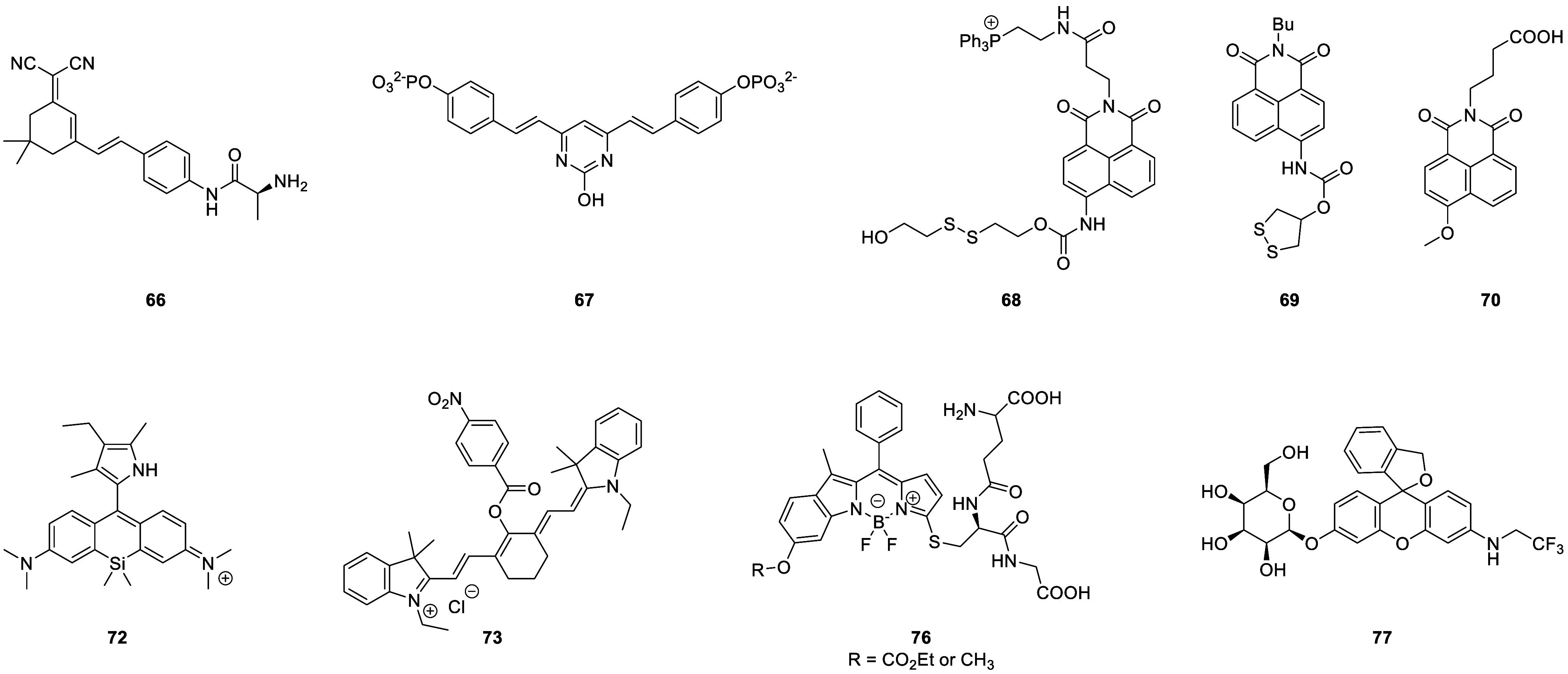
Selected fluorescent probes for liver, lung, and ovarian cancer.

Liver cancer is the fourth leading cause of death, and the sixth most common cancer globally, representing a significant health challenge across the globe. Hepatocellular carcinoma (HCC) is by far the most prevalent, accounting for over 80% liver cancer diagnoses.^130^ Liver cancer is typically associated with damage and scarring, usually caused by chronic inflammation, alcohol abuse, hepatitis, or fatty liver disease.^131^ Because early symptoms of liver cancer are nonobvious, many patients are diagnosed in mid or late stages, which significantly delays treatment and thus worsens prognosis.^132^ Early detection of liver cancer is therefore crucial to achieve early intervention, which can for instance be achieved by fluorescent detection of cancer-related biomarkers (Figure 26).^133^

A major issue in cancer treatment is the risk of recurrence, often caused by incomplete surgical resection. The development of tools that can accurately and rapidly distinguish normal tissue from tumor tissue is therefore an attractive target, as they could allow for effective and complete removal of cancerous tissue, reducing the risk of recurrence. As for glioma (*vide supra*), CD13/aminopeptidase N (APN) is an important specific marker, as it mediates the development and metastasis of liver cancer. In 2020, Peng et al. therefore developed APN-responsive fluorescent probe **66** to monitor endogenous APN activity to guide surgical resection.^134^ Probe **66** consists of two parts: a dicyanoisophorone fluorophore as the fluorescence reporting unit, and an l-alanine element as the recognition site. On interaction with APN, l-alanine is cleaved, releasing the amine group of dicyanoisophorone to restore ICT, leading to fluorescence at 650 nm. *In situ* spraying was used to distinguish tumor from normal tissue using this fluorescence change, with fluorescence intensity ratios (tumor/normal, T/N) of 13.86 (subcutaneous tumor) and 4.42 and 6.25 (hepatic and splenic metastasis, respectively) being obtained. Most significant is that using probe **66** tumor metastases smaller than 1 mm could be precisely identified and resected, an impressive step in demonstrating the use of enzyme-activated fluorescent probes for cancer diagnosis and image-guided surgery.

In 2012 Yao et al. developed a TP fluorescent probe **67** for the imaging of endogenous phosphatase activity.^135^ A TP 2-hydroxy-4,6-bis(4-hydroxy-phenyl)pyrimidine dye was modified to suppress fluorescence by attaching electron-withdrawing phosphate groups to both its phenolic hydroxyl units. Exposure of probe **67** to phosphatase triggers hydrolysis of these groups, restoring fluorescence to elicit a signal capable of monitoring endogenous phosphatase. With this probe, the authors successfully observed changes in endogenous phosphatase activity in hepatocellular carcinoma cells. In addition, probe **67** could also be used to observe the endogenous phosphatase activity in *Drosophila* brain at a depth of 100 μm.

In 2012, Kim and co-workers designed mitochondrial targeting probe **68** for imaging analysis of mitochondrial thioredoxin (Trx) activity,^136^ composed of naphthalimide as the fluorophore, triphenylphosphine as the mitochondrial targeting unit, and a disulfide bond as the fluorescence switch. In the presence of Trx, the disulfide bond is reductively cleaved to generate a thiol capable of intramolecular attack of the adjacent carbamate to release the fluorescent amino naphthalimide. Using probe **68**, Trx could be detected at concentrations as low as 53 nM, allowing the authors to observe Trx activity in the mitochondria of HepG2 cancer cells. This provides a means of better understanding the biological function of Trx.

Conversely, probe **69** was designed by Fang et al. in 2014 for the detection of mammalian thioredoxin reductase (TrxR).^137^ Probe **69** comprises two parts: a naphthalimide as the fluorescent group, and a 1,2-dithiane as the reacting “sensing” unit. Similar to TrX based probe **68** above, this probe works by reductive cleavage of the disulfide bond to release a free thiol which intramolecularly deprotects the amine to regenerate the fluorescent amino-naphthalimide. Probe **69** was shown to exhibit good sensitivity and selectivity, where it can distinguish between cell extracts with different TrxR activities. Furthermore, fluorescence cell imaging experiments using this probe indicated that the fluorescence signal varied with the activity of TrxR in tumor cells, providing a useful tool for screening TrxR inhibitors and for further exploring TrxR-mediated physiological processes.

In 2015, Yang et al. reported a ratiometric TP fluorescence probe **70** for detecting human cytochrome P450 1A (CYP1A).^138^ Starting from a naphthalimide core as the fluorophore, C-4 substituent screening was carried out to modulate the system’s ICT fluorescence. Use of a methoxy group at this position gave good activity for CYP1A, and introduction of a *N*-carboxypropyl substituent led to good selectivity for CYP1A over other CYP isoforms. Through oxidative cleavage of the methoxy C–O bond, probe **70** was converted by CYP1A to release the fluorescent 4-hydroxy naphthalimide. *In vitro* experiments showed that an increase in CYP1A concentration led to a decrease in fluorescence at 452 nm and a corresponding increase at 564 nm, allowing ratiometric determination of CYP1A concentrations in cancer-relevant cell assays such as HepG2 cells and hepatocytes.

**Figure 27:**
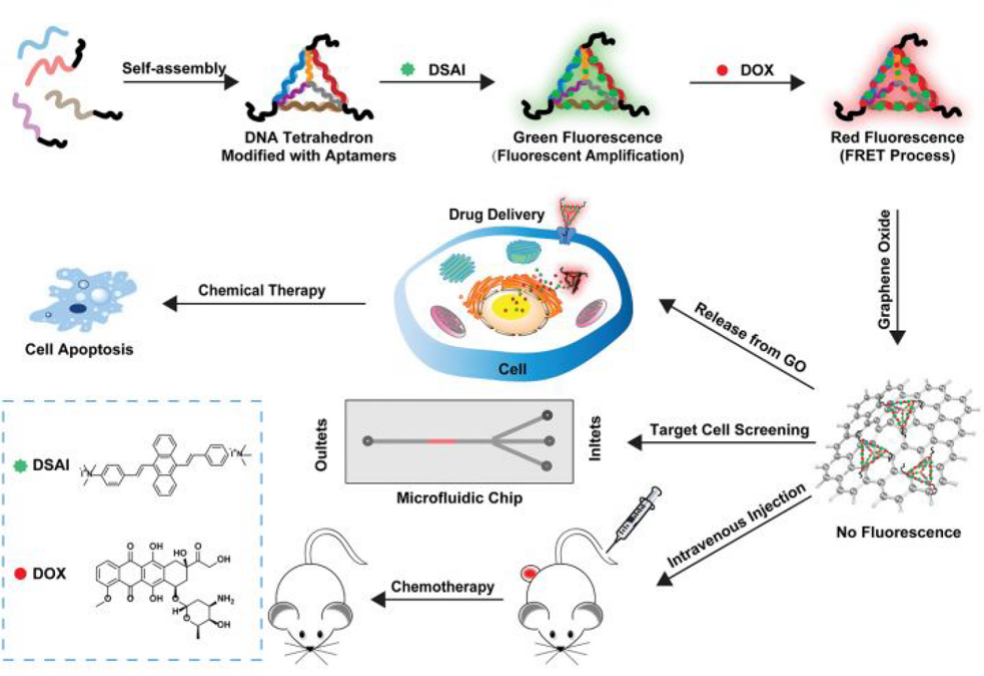
Graphene oxide fluorescent DNA material (probe **71**) for the diagnosis and treatment of liver cancer. Reproduced with permission from ref (139). Copyright 2021 Wiley-VCH.

In 2021 Tang et al. developed a graphene oxide-based fluorescent nanomaterial (probe **71**) for the diagnosis and treatment of liver cancer *in vivo*.^139^ The material was composed of self-assembled aptamer-modified ssDNA, doxorubicin (DOX, a chemotherapy agent), and an AIE-based fluorophore DSAI (Figure 27). The four ssDNAs self-assemble into a DNA tetrahedron (DNA-tetra) structures, to which the DOX and the DSAI units are appended. The FRET process between DOX and DSAI enhances the fluorescence of DOX, leading a red emission from the assembled material. The hairpin aptamer modifications (on the ssDNA scaffold) enable it to strongly hydrogen bond to the graphene oxide support, which concomitantly quenches the fluorescence. When the probe enters liver tumor cells, the aptamer is released from the graphene oxide surface, along with the DNA nanomaterial, releasing DOX and DSAI, eliciting both a fluorescent signal and a therapeutic response. Good biocompatibility was observed, for both *in vitro* and *in vivo* experiments, demonstrating that these complex materials could not only be used in the diagnosis of liver cancer through imaging, but also for therapeutic effect. This new multifunctional material demonstrates the opportunities that these types of assemblies can be afforded for the development of multicomponent materials for delivery, diagnosis, and therapy.

### 3.3 Fluorescent Probes for Lung Cancer

According to the WHO, over 2.21 million new lung cancer patients and 1.8 million lung cancer deaths occurred in 2020, cementing its place as the deadliest form of cancer.^140^ At present, the main causes of lung cancer are smoking, chronic pneumonia, and environmental exposure.^141^ The symptoms of lung cancer are complex, and its clinical presentation mostly depends on the location and type of tumor. As with liver cancer, early symptoms of lung cancer are mild or nonexistent, making early detection a significant challenge (Figure 26).

Looking again at ROS, probe **72** was developed by Guo and co-workers in 2017 for the specific imaging of ROS in lung tumor cell lysozymes.^142^ The fluorescence of the Si-rhodamine core in **72** is quenched through PeT with the attached pyrrole. This pyrrole can be readily oxidized by highly reactive ROS such as HOCl, •OH and ONOO^–^ (see breast cancer probe **60**), neutralizing the quenching causing the probe to emit a red 680 nm fluorescence signal. Probe **72** was found to have good stability against both autoxidation and photooxidation. In a human model of nonsmall cell lung cancer stimulated by β-rapadone, the probe successfully imaged ROS changes in cancer cell lysosomes in real time, demonstrating its ability to distinguish between normal and cancer cells.

Li and Feng et al. developed probe **73** in 2015, a NIR based fluorescent probe for the detection of nitroreductase (NTR) activity in hypoxic tumors.^143^ The authors explored five cyanine dye-derived probes with different modifications, finally selecting the design of probe **73**. Probe **73** features a *p*-nitrobenzoate, which is readily reduced to *p*-aminobenzoate by NTR to enhance fluorescence. The results of the *in vitro* studies showed that probe **73** has a good response, with a 110-fold fluorescence enhancement upon reduction by NTR. Cell and *in vivo* experiments confirmed that this fluorescent probe can specifically image NTR in tumors and can be used to characterize hypoxia at tumor sites, highlighting that many simple-to-synthesize, low-cost sensors/chemodosimiters can often yield excellent results and provide fantastic insights into complex biomolecular processes.

**Figure 28:**
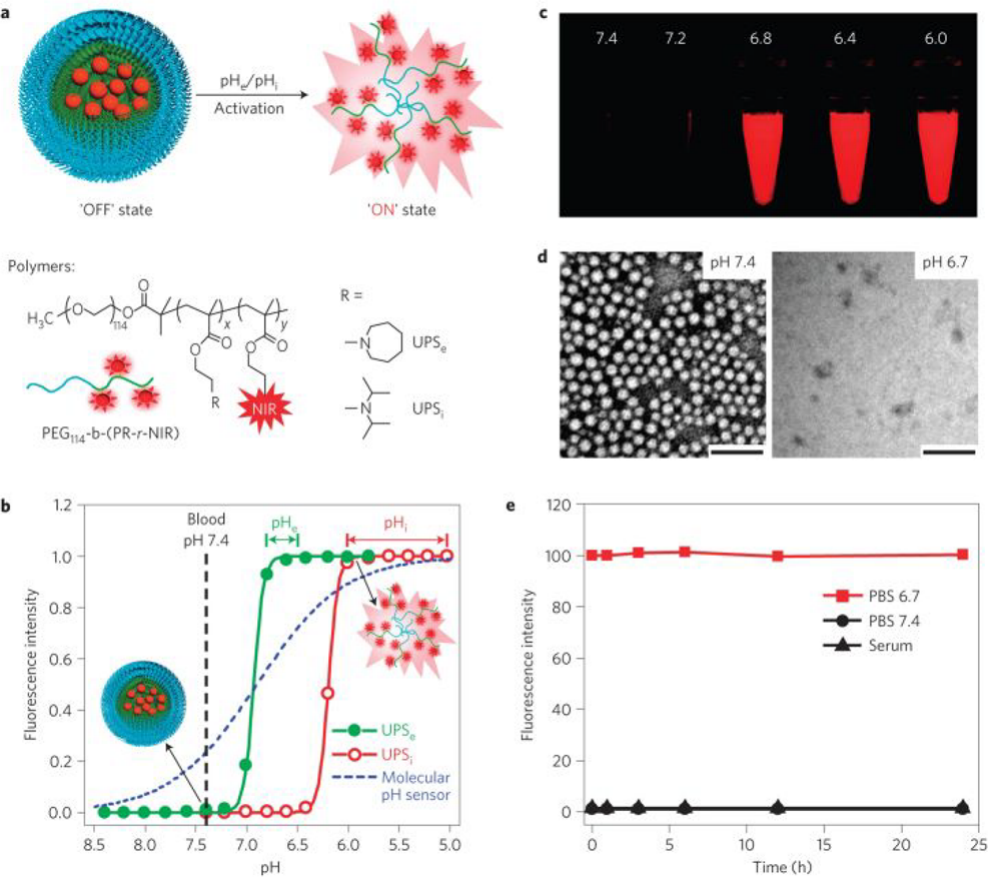
(a) Structure of UPSe (probe **74**) and UPSi (probe **75**). (b) Normalized fluorescence intensity as a function of pH for UPSe and UPSi nanoprobes. (c) Fluorescent images of UPSe–Cy5.5 nanoprobe solution in different pH buffers. (d) Transmission electron micrographs of UPSe nanoprobes. (e) Stability experiment of UPSe nanoprobes. Reproduced with permission from ref (144). Copyright 2014 Springer Nature.

Finally, it must be noted that pH is often a key variable in tumors, and so in 2014 Gao et al. built a pH-sensitive probe for the detection of tumor tissue in mice (Figure 28).^144^ Their nanoprobe (probes **74** and **75**) consists of three components: a unique hydrophobic micelle as the pH-sensitive core, an RGD sequence as the targeting group, and a cyanine fluorophore. The number of pH recognition units and fluorescence units supported by each nanoparticle was carefully optimized to ensure a maximized response. Employing this probe, the authors were able to accurately distinguish tumor tissue from normal healthy tissue.

### 3.4 Fluorescent Probes for Ovarian Cancer

Although the incidence of ovarian cancer is relatively low compared to other cancers such as those already discussed, its mortality rate is extremely high. This is primarily due to its unclear pathogenesis, and the high risk of metastasis prior to diagnosis, presenting a bleak prognosis (Figure 26).^145,146^

In 2015, Fan et al. developed two novel fluorescent probes **76** (where R is CO_2_Et or Me) for specific imaging of GGT in tumor cells.^147^ These probes consist of two parts: the GGT-specific GSH unit and the BODIPY fluorophore. After cell penetration, GGT cleaves the glutamate from the GSH chains in these structures, leading to intramolecular S-to-N rearrangement via an S_N_Ar mechanism to produce an amino-substituted BODIPY-Cys with significantly increased fluorescence (ca. 12-fold). Using probe **76**, the authors successfully distinguished ovarian cancer cells from normal cells.

In 2015, Urano et al. demonstrated a high-sensitivity rhodamine-based β-galactosidase fluorescent probe **77**, engineered by optimizing the lactone-zwitterion equilibrium of the rhodamine core.^148^ The authors structurally modified the probe and as such, adjusted its p*K*_cycl_ value to approximately 5.4, so that the probe would exist exclusively (>99%) as the nonfluorescent spirocyclic in pH 7.4 buffer (this greatly reduced the background fluorescence that often plagues rhodamine-based systems). This probe was shown to selectively detect β-galactosidase activity *in vitro*, and more significantly, the authors developed seven mouse models of ovarian metastatic cancer and were able to consistently observe metastases. It is hoped that this probe and others like it could potentially find clinical applications in ovarian cancer detection and that the flexibility of this scaffold might allow it to be applied to the detection of multiple tumor-associated active enzymes.

**Figure 29:**
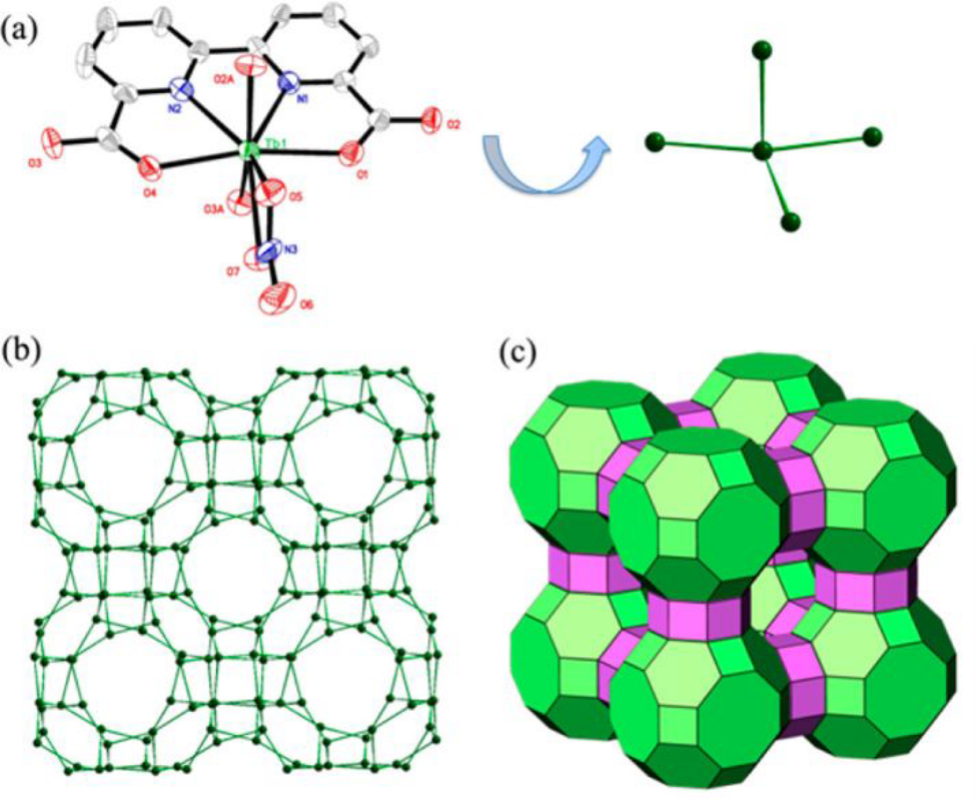
(a) Crystal structure of Tb-ZMOF (probe **78**). (b) Perspective view of the α- and β-cages in Tb-ZMOF (dots = Tb^3+^; lines = carboxylate). (c) Tiling representation of the rho topology of Tb-ZMOF. Reproduced with permission from ref (149). Copyright 2015 American Chemical Society.

In 2015, Zaworotko et al. developed two lanthanide zeolite-like metal–organic frameworks (Ln-ZMOFs) with Rho topologies (Figure 29, probe **78**).^149^ The material was easily synthesized by self-assembly of 4-linked lanthanide molecular building blocks and bipyridine–dicarboxylic acid ligands. By adjusting the ratio of Tb^3+^ to Eu^3+^ (which give rise to delayed lanthanide centered emission), the authors obtained materials capable of detecting lysophosphatidic acid, a biomarker for ovarian cancer present in plasma. This design provides a new idea for the development of luminescent mixed crystal Ln-MOF, with the potential for the development of a new suite of tools with possible applications in tumor detection.

### 3.5 Fluorescent Probes for Cervical Cancer

**Figure 30:**
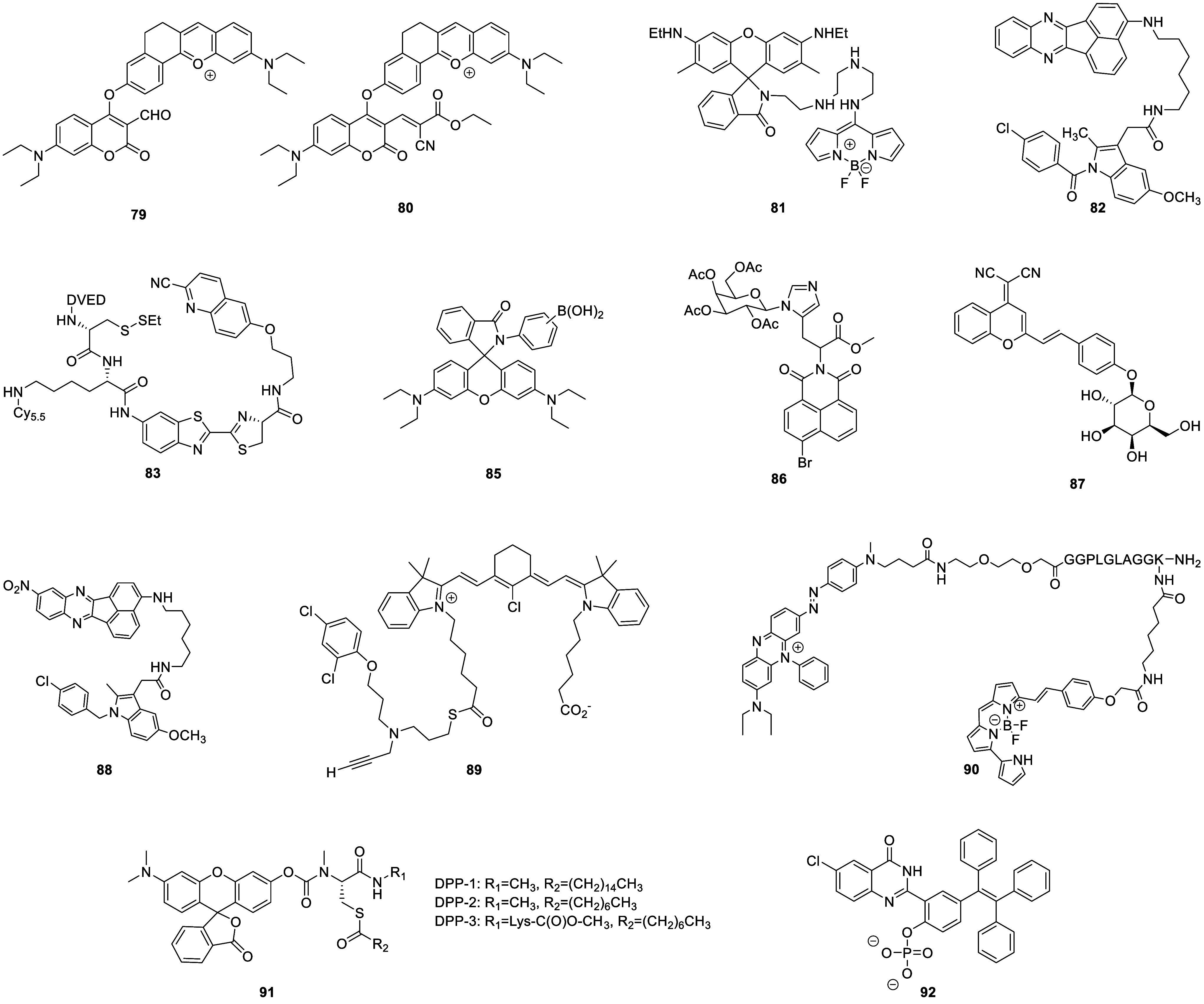
Selected fluorescent probes for cervical and other selected forms of cancer.

**Figure 31:**
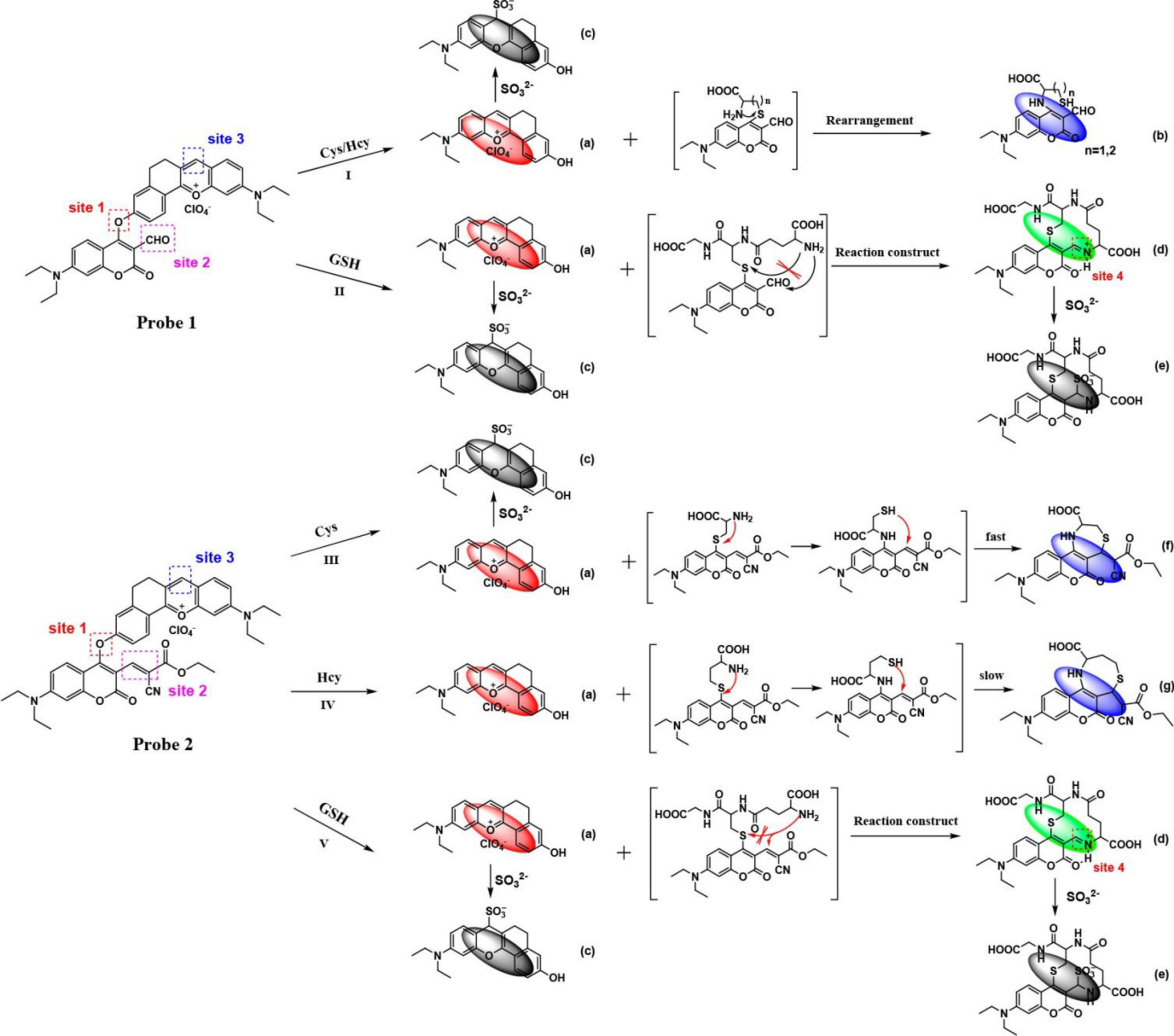
Response mechanisms of probes **79** and **80** to thiols and SO_2_. These give several different products as indicated using small letters to identify them. Reproduced with permission from ref (151). Copyright 2006 American Chemical Society.

Cervical cancer is the fourth most common form of cancer, with over 600,000 new cases in 2020. Its incidence is closely related to high-risk human papillomavirus (HPV) infection.^150^ Current cervical cancer treatment rates are high, and even patients with advanced cervical cancer diagnoses can have their tumor growth effectively controlled through appropriate treatment and removal (Figure 30). Yin et al. demonstrated two novel fluorescent probes, **79** and **80**, respectively, in 2020, that enable differential monitoring of mercaptans and SO_2_ biomarkers of cervical cancer by introducing different reaction sites to the same general scaffold (Figure 31).^151^ These structures were based around three different reactions sites. Reaction site one is common to both probes, and consists of a biaryl ether linkage, capable of undergoing cleavage by addition Cys, Hcy, or GSH thiols to produce a red fluorescent pentacyclic pyrylium fluorophore. This species can then react with any SO_3_^2–^ (SO_2_ donor) present at reaction site three, breaking the conjugated system by sulfonylation, suppressing the red fluorescence. Reaction site two, unique to each probe, reacts intramolecularly, with the added thiol compound from the ether linker cleavage, producing a new cyclic thiane-coumarin that exhibits blue fluorescence. In the case of the second probe, addition of GSH leads to the production of a green fluorescent species containing a thiane and an imine, which can itself be decomposed on further reaction with SO_3_^2–^. This complex cascade of reactions is shown in Figure 31. Based on this, the authors successfully detected mercaptan and its metabolites using these fluorescence changes. The results of *in vivo* and *in vitro* imaging experiments demonstrated the usability of these probes, and its ability to differentially detect thiol and sulfur dioxide analytes. These probes illustrate a nice proof of concept for the development of complex multisite: multireactivity probes with potential applications for monitoring complex and competing metabolic processes.

In 2018, Ahn et al. designed TP fluorescent probe **81** for imaging analysis of lysosomal ATP concentration changes.^152^ Probe **81** is composed of two fluorophores; amino-BODIPY and rhodamine 6G, connected by a tetramine chain that plays a key role in recognizing ATP in this design. In the absence of ATP, the probe fluoresces at 454 nm under excitation at 403 nm. Addition of ATP leads to FRET between the amino-BODIPY and rhodamine 6G, causing the probe to exhibit yellow fluorescence at 557 nm. This probe enabled the authors to visually monitor kiss-and-run and full-collapse fusion processes in HeLa cells (cervical cancer cell) and quantitatively analyzed the ATP concentration in lysozymes during these processes *in vivo*.

In 2013, Peng et al. developed probe **82** for the fluorescent imaging of cyclooxygenase-2 (COX-2) in the Golgi apparatus of cancer cells.^153^ Probe **82** uses the fluorophore acenaphtho[1,2-*b*]quinoxaline (ANQ) and indomethacin (a potent COX inhibitor) as the COX-2 targeting group. In aqueous solution, the probe adopts a folded conformation, and PeT is active between the ANQ and IMC units, resulting in the quenching of the ANQ fluorescence. When the probe binds to COX-2 on the Golgi apparatus it is unfolded, PeT quenching is now inhibited, and the fluorescence is turned on. Using probe **82**, the authors imaged COX-2 activity in different cells and were able to rapidly distinguish between normal and cancer cells (HeLa cells). Furthermore, probe **82** could be used to observe the dynamic changes of the Golgi apparatus during the process of tumor cell apoptosis.

Rao et al. built a small molecule fluorescent probe **83** in 2014 that can self-assemble *in vivo* to allow for the imaging of caspase activity.^154^ Probe **83** consists of two main parts: an amino-luciferin scaffold, connecting the d-cysteine and 2-cyano-6-hydroxyquinoline groups, and a L-DEVD (Asp-Glu-Val-Asp) capping peptide sequence, as well as a disulfide bond for caspase-3/7-mediated cleavage and intracellular thiol-mediated reduction. This probe could be selectively activated by caspase-3/7 to trigger bioorthogonal macrocyclization and nanoaggregation, thus achieving the effective monitoring of tumor therapeutic response by visualizing caspase (i.e., apoptosis). The design was validated both in HeLa cells and in HeLa tumor-bearing mice.

In 2006, Kwon et al. also reported DEVD-based probe **84** for imaging apoptosis, this time employing nanoparticles.^155^ These nanoparticles were appended with a Cy5.5 fluorophore and the caspase specific DEVD. Fluorescence of the initial unactivated system is quenched simply by virtue of tight binding of the dye to the nanoparticle. After reaction with the enzyme, the peptide segment is cleaved and the spacing is increased, which results in less quenching, and the NIR signal is switched on. *In vitro* assays showed that the fluorescence intensity of the nanoparticles was enhanced by a factor of 10 for caspase-3 and caspase-7. Using this probe, the authors successfully observed the contraction and formation of membrane vesicles in HeLa cells, allowing for visual analysis and monitoring of cancer cell apoptosis.

### 3.6 Fluorescent Probes for Other Cancers

Having looked at structurally and mechanistically varied fluorescent probes for five of the most common cancers, this short section will present some interesting examples of probes that have been developed for a variety of other cancers and tumors.

In 2016 Chang et al. demonstrated a multisite binding fluorescent probe **85**, which responds to rapid changes in intracellular ATP levels.^156^ Rhodamine B was used as the fluorescent unit, and a phenylboronic acid group was introduced to maintain the closed-loop nonfluorescent state. On addition of ATP, multiple covalent and noncovalent interactions promote switching to the “open” rhodamine form. These include covalent formation of a boronate ester between the boronic acid and ATP ribose, π–π interaction between the anthracene of **85** and the adenine ATP, and electrostatic and hydrogen bonding between the probe diethylamine groups and the ATP phosphate units, respectively. In its open configuration, the probe becomes fluorescent, thus emitting bright red light on ATP binding. *In vitro* experiments show that the probe can respond to ATP quickly and specifically, with an approximately 18-fold fluorescence enhancement on exposure to ATP. Cell assays demonstrated low biotoxicity, good cell penetration, and mitochondrial localization. Using probe **85**, the authors were able to observe changes in ATP levels in oral squamous cell carcinoma (OSCC) and HeLa cells. It is interesting to note that due to the noncovalent nature of most of the sensing interactions, and the facile reversibility of boronate ester formation, the sensing event can be readily reversed, with for instance the addition of a pyrase (ATP hydrolyzing enzyme) reversing the process and switched off fluorescence by removing the ATP from the system, thus regenerating probe **85** in its closed spirocyclic form.

In 2017, Martinez-Manez and Serrano designed a TP fluorescence probe **86** to visualize tumor cell aging *in vivo*.^157^ The authors appended a fluorescent naphthalimide to the *N*-terminus of l-histidine methyl ester, and then connected acetylated galactose to the imidazole ring of the amino acid to afford the fluorescent probe. In cells the glycoside is cleaved by β-galactosidase, releasing the fluorophore to produce a 286-fold fluorescence enhancement. The authors used palbociclib to induce senescence (aging) of SK-MEL-103 (human melanoma) cells, which could be observed using probe **86**, providing evidence of its ability to target and image senescent cells. When injected into a mouse model of subcutaneous melanoma that had been treated with palbociclib to induce senescence, the probe was found to fluoresce at the tumor sites, allowing the identification and visualization of said tumors.

In 2016, Zhu and Guo et al. also designed ratiometric NIR fluorescence probe **87** to visualize β-galactosidase activity in colorectal cancer.^158^ This probe uses a DCM fluorophore with a galactose-masked *p*-hydroxy group acting as the fluorescent masking enzyme-cleavable trigger. On exposure to β-galactosidase, the galactose unit is removed as with probe **87**, resulting in bright fluorescence at 685 nm and a decrease in signal at 500 nm, enabling ratiometric tracking of the galactosidase analyte. Furthermore, this probe boasts good photostability, and was also shown to be useful for real-time tracking of galactosidase both in cells (293T tumor, OVCAR-3 ovarian cancer) and *in vivo* in a mouse colorectal tumor model. Related to this work is that of Scanlan and Gunnlaugsson, who developed several examples of naphthalimide conjugated glycan structures, as glycosylated-Nap probes and prodrugs.^159,160^ These have the ability to undergo glycosidase-mediated activation in various cancer cell lines where the nature of the glycan and the enzyme that is overexpressed in the cancer cells dictates the release of the drug *in situ*, allowing for the real time monitoring of the uptake and activity.

COX-2-targeting fluorescent probe **88** was developed by Peng et al. in 2013 and is capable of distinguishing between cancer and inflammation.^161^ This probe was constructed by attaching the COX-2 substrate indomethacin to the fluorophore NANQ using a linear alkyl diamine spacer. In the absence of COX-2, the probe adopts a lower energy folded conformation, which results in fluorescence quenching. However, when the indomethacin group binds to COX-2, conformational switches occur, forcing probe **88** to adopt an unfolded conformation, concomittanly interrupting the quenching of NANQ by IMC and restoring the fluorescence. The authors found that this probe showed enhanced fluorescence signals in both inflammation and tumor models. In both systems, fluorescence emission centered at 615 nm increased on exposure to COX-2. However, as the concentration of COX-2 (0.12–3.32 μg/mL) is increased further, the signal at 615 nm decreases, while the fluorescence at 555 nm is increased. Because cancer and inflammation express different levels of COX-2 enzyme, this can be used to differentiate the two environments, as their fluorescence emission is noticeably different. By spraying with probe **88**, cancerous, normal, and inflamed tissue could be easily differentiated by eye using a simple hand-held UV lamp. The emergence of this type of probe (see probe **66** above) is expected to provide major potential clinical applications for tumor detection and identification and surgical guidance for tumor resection.

In 2015, Shih et al. reported NIR probe **89** for targeting monoamine oxidase A, useful for the detection and treatment of prostate cancer.^162^ The probe consists of two parts: monoamine oxidase A inhibitor clorgyline as the targeting group, and a fluorescent heptamethine carbocyanine dye as the fluorophore. This probe was shown to not only accurately locate the desired tumors but also effectively inhibit their further growth and spread, affording a viable theranostic manifold for future development.

In 2012, Nagano et al. designed probe **90**, a FRET-based system for visualizing matrix metalloproteases (MMP).^163^ The dark quencher BHQ-3 was used as a FRET receptor, paired with a NIR BODIPY fluorophore donor, connected by an MMP peptide substrate to form probe **90**. Under abnormal conditions, FRET from the BODIPY to BHQ-3 leads to fluorescence quenching. In the presence of MMP, however, on severing of the polypeptide, the FRET pair is separated, and fluorescence is “switched on”. *In vitro* experiments indicated that probe **90** was highly permeable and aggregated effectively in target cells. Fluorescence confocal imaging experiments of mouse xenograft tumor models showed that probe **90** could indeed detect MMP activity. Compared with previously reported imaging tools, the probe designed in this study has the advantages of fast response and high signal-to-noise ratio, and as such should provide a useful tool for researchers to detect MMP in cancer.

In 2017, Dickinson et al. developed a small molecule fluorescent probe **91** for detecting cysteine *S*-depalmitoylation in cells.^164^ They attached palmitoacylated cysteine residues to rhodamine dyes using a carbamate linker, forcing rhodamine into its lactone low-fluorescence form. In the presence of acylprotein thioesterases (APT, the depalmitoylation enzyme), the thiolester of the probe is deacetylated to reveal the thiol, which intramolecularly cleaves the carbamate linkers, concomitantly releasing the rhodamine dye, which is now free to ring-open and fluoresce. Using probe **91**, the authors successfully imaged endogenous APT activity in A431 cells and HEK293T cells. Additionally, the signals demonstrated by the probe revealed a new mechanism of dynamic lipid signaling, providing insight into a poorly understood and understudied aspect of protein regulation.

In 2017, Tan et al. reported a new type of solid-state ESIPT-based probe **92** for visualizing ALP in living cells.^165^ In its unactivated pre-analyte form, the ESIPT process is prevented by the addition of a phosphate group to mask the hydrogen-bond donor phenol, suppressing any ESIPT capability and switching off the fluorescence. This also has the effect of solubilizing the probe in aqueous media. On phosphate cleavage by ALP, probe **92** is released and rapidly precipitates, resulting in bright solid-state fluorescence. Probe **92** was shown to successfully detect ALP activity with a high signal-to-noise ratio and was able to detect endogenous ALP activity in osteosarcoma cell lines (U-2OS and Saos-2) with high resolution. This probe has obvious value for exploring the physiological and pathological function of ALP. Furthermore, this novel design is expected to provide an interesting new platform for the development of new sensors for detecting tumor biomarkers.

**Figure 32:**
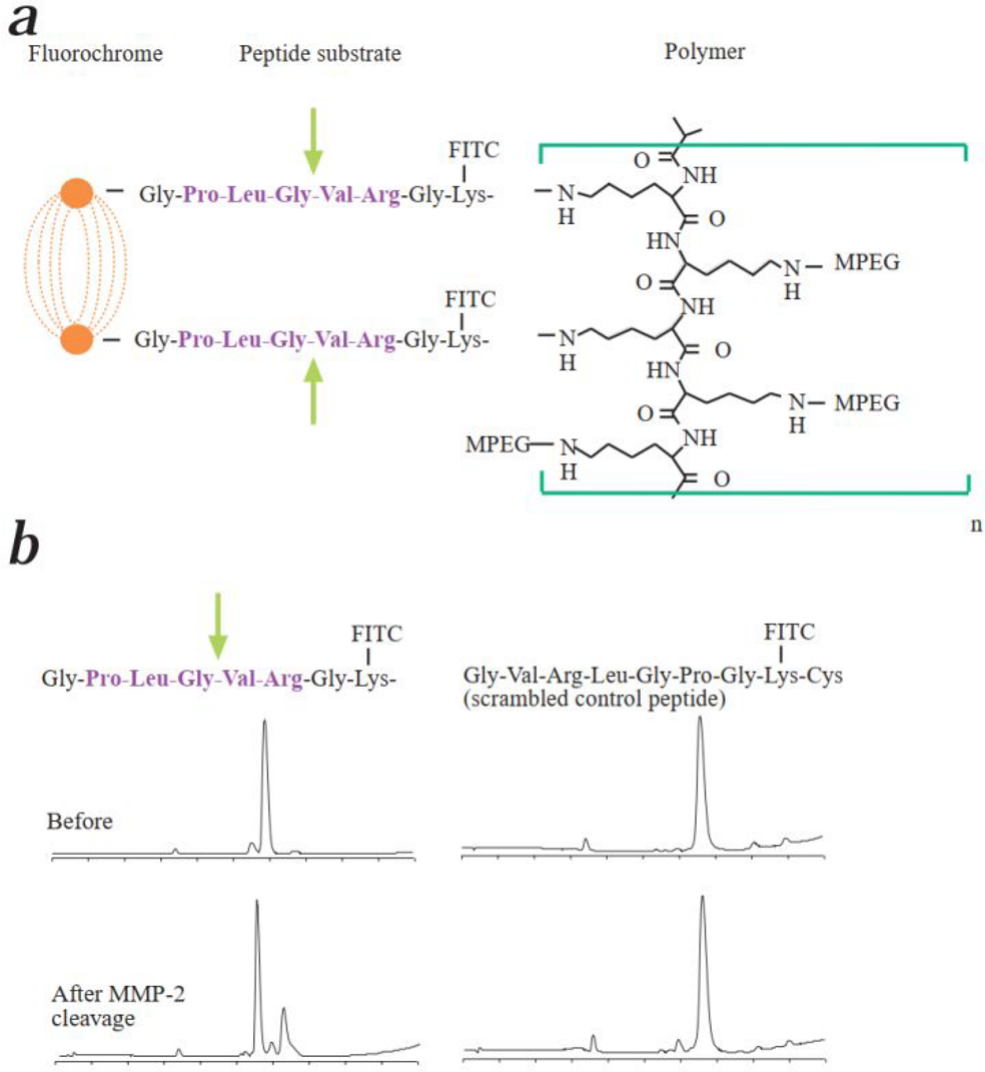
(a) Structure of the MMP-2-sensitive probe **93**. (b) HPLC traces of peptide substrates before and after MMP-2 cleavage. Reproduced with permission from ref (166). Copyright 2001 Springer Nature.

In 2001, Welssleder et al. reported a biocompatible near-infrared fluorescent probe for detecting MMP activity in mouse tumors (Figure 32, probe **93**).^166^ The authors attached a near-infrared dye to MMP-2 peptide substrate and fixed this to a nonimmunogenic polymer backbone. The polymer’s fluorescence is initially quenched due to the close packing of this material. However, as the peptide is cleaved by MMP2, the fluorescence emission is restored, producing a NIR signal at the site of the tumor. The authors performed imaging experiments in a tumor-bearing mouse model of human fibrosarcoma cells (HT1080), demonstrating that this polymeric sensing system can effectively detect changes in MMP-2 activity and can be used to evaluate MMP activity.

**Figure 33:**
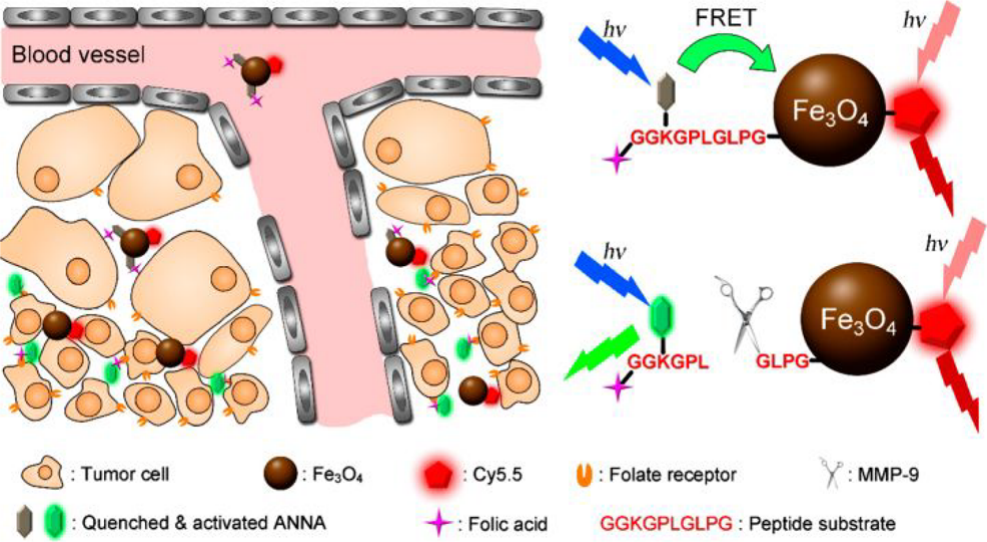
Mechanism of action of ANNA nanoprobes (probe **94**) in tumor cells. Reproduced with permission from ref (167). Copyright 2018 American Chemical Society.

In 2018, Gao et al. designed a dual-ratiometric fluorescent probe for visualizing changes in both tumor-associated protease activity and pH (Figure 33, probe **94**).^167^ The pH-sensitive fluorescent dye ANNA was attached to the surface of Fe_3_O_4_ nanoparticles via a polypeptide sequence, switching off the fluorescence of ANNA by FRET. In addition, Cy5.5 was attached to the probe, remaining “on” throughout to act as the fluorescent reference. On cleavage by protease MMP-9, FRET is interrupted, and ANNA resumes fluorescence, signaling protease activity. The activity of MMP-9 could be determined by comparing the constant fluorescence of Cy5.5 with that of MMP-dependent ANNA. *In vivo* and *in vitro* results showed that this probe successfully provided quantifiable information on the activity and pH of MMP-9 protein at the tumor site.

## 4 Fluorescent Probes for Organ Damage

The human body is complex, with a multitude of organs, each of which plays a vital role in the continued healthy function of the body as a whole. Maintenance of each organ is therefore crucial, and internal organ damage can often lead to severe health issues. The organs most vulnerable to damage are the brain, kidneys, and liver, which this section will focus on. Early symptoms of organ damage are often easily ignored, causing worsening damage and serious disease. Timely detection and better understanding of such conditions is therefore key, and fluorescence imaging (Table 3) is an increasingly important tool for detecting the occurrence and development of organ damage, boasting the many advantages already highlighted throughout earlier sections of this review.

**Table 3:** Selected Fluorescent Probes for Organ Damage

probe	λ_ex_/λ_em_ (nm)	LOD	bioactive molecule	biological model	ref
Liver Injury					
**95**	450/555	0.13 μM	ONOO^–^	ALI mice	(168)
**96**	450/562, 450/568, 520/587		ATP, ONOO^–^	HL-7702 cells	(169)
**97**	540/705	33 nM, 100 nM, 40 nM	Cys, Hcy, GSH	DILI mice	(170)
**98**	820/864		lysosomal viscosity in hepatocytes and mice during HIRI	HIRI mice	(171)
**99**	530/560	25.9 μM, 0.628 μM	ATP, H_2_S	HepG2 cells	(172)
**100**	988/1058		HClO	ALI mice	(173)
**101**	380/470, 640/660	6.38 nM, 6.09 nM	O_2_^•–^, ONOO^–^	HIRI mice	(174)
**102**	808/1053, 980/1525	0.7 nM	H_2_S	metformin-induced liver injury mice	(175)
**103**	808/980	0.46 μM	ROS	HIRI mice	(176)
**104**	675/720	10 × 10^–9^ M	O_2_^•–^	HIRI mice	(177)
**105**	540/660,	154 × 10^–9^ M.	ONOO^–^	CCl_4_-dependent acute hepatitis	(178)
**106**	660/810	241 × 10^–9^ M		Balb/c mice	
Kidney Injury					
**107**	490/635	0.057 U/L	GGT	zebrafish	(180)
**108**	420/515	0.16 μM	O_2_^•–^	AKI mice	(183)
**109**	808/-	16.2 μM	H_2_O_2_	unilateral ureteral obstruction mouse model	(184)
**110**	-/700	13 × 10^–9^ M,	O_2_^•–^,	cisplatin-induced	(185)
**111**		17 × 10^–9^ M	ONOO^–^	AKI mice	
**112**	675/720	12 nM	O_2_^•–^, NAG	contrast-induced AKI mice	(186)
**113**	-/800		pH	acidosis-induced kidney injury mouse model	(188)
**114**	790/808	0.079 nM	caspase-3	cisplatin-induced AKI mice	(189)
**115**	675/720		γ-glutamyl transferase	cisplatin-induced AKI mice	(190)
**116**			Kim-1	rhabdomyolysis-induced AKI mice	(191)
**117**	808/1000–1100		N/A	kidney dysfunction in murine model	(192)
**118**	660/910		N/A	renal fibrosis mice	(193)
**119**	698/725		N/A	renal ischemia-reperfusion mice	(194)
	730/790				
	825/912				
	890/1025				
Traumatic Brain Injury					
**120**	800/I595/I453	55.4 nM	HOCl	BV-2 cells	(198)
**121**	1050/1094		ONOO^–^	brain vascular injury mice	(199)
**122**	808/1071		ROS	TBI mice	(200)
**123**	480/530		ROS	TBI mice	(201)
**124**	480/645		blood	SAH mice	(202)
**125**	745/800		N/A	TBI mice	(203)

### 4.1 Fluorescent Probes for Liver Injury

**Figure 34:**
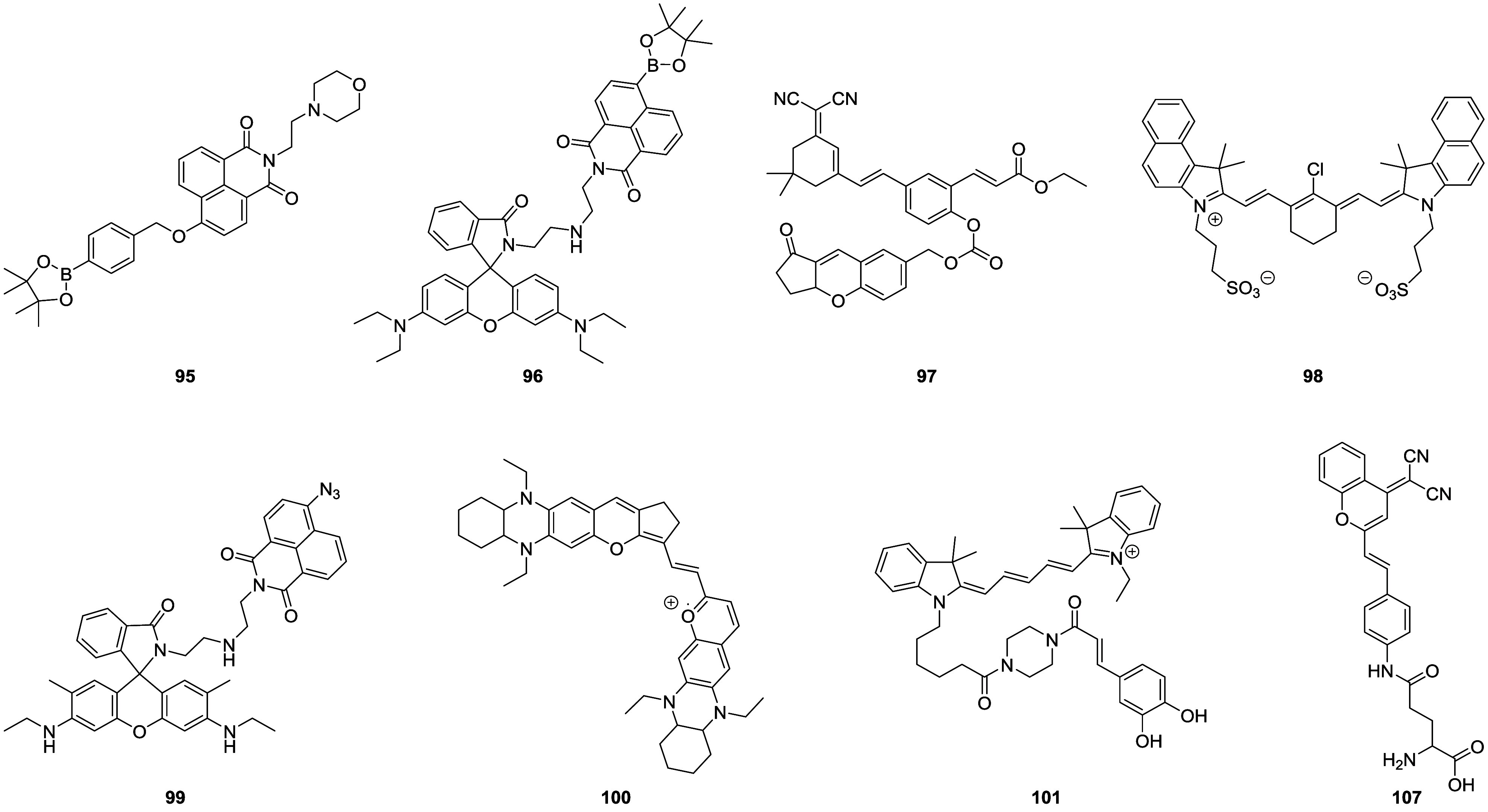
Selected fluorescent probes for liver injury.

Liver injury is primarily caused by drug induced hepatitis, chronic viral hepatitis, or trauma, with early symptoms going unnoticed or ignored due to lack of public awareness. Liver injury can refer to acute and drug induced liver damage, liver ischemia reperfusion injury, etc. The key biomarkers in this case, on which this section will focus, include many of those seen previously, such as reactive oxygen species, metal ions, liver enzymes, and ATP (Figure 34).

As a hallmark of oxidative stress, ONOO^–^ is a common target for imaging and studying liver injury, with a broad range of fluorescent probes reported in the literature for its detection. One such example is probe **95**, a naphthalamide-based system developed by Wang and co-workers in 2023, designed for measuring lysosomal ONOO^–^ levels in acute liver injury model mice.^168^ In the absence of ONOO^–^, probe **95** showed only a weak absorption at 450 nm, which increased on oxidative cleavage of the boronate ester by ONOO^–^, with an associated increase in fluorescence response at 555 nm. Probe **95** exhibited high selectivity for ONOO^–^, fast response time (approximately 70 s), a good LOD of 0.13 μM, and low cytotoxicity. Confocal fluorescence imaging using probe **95** in phorbol-12-myristate-13-acetate (PMA)- or lipopolysaccharide (LPS)-induced LX-2 cells showed good results, with probe **95** successfully monitoring increases in endogenous ONOO^–^ levels. The probe also showed good results for visualizing elevated ONOO^–^ concentrations in mice with acute liver injury.

ONOO^–^ is well-known to have deleterious effect on the synthesis of ATP, in part by inactivating ATP synthase. Because ATP plays a crucial role in the majority of key cellular processes, ONOO^–^ (and therefore more generally oxidative stress) can cause serious systemic issues. To gain a deeper understanding of the link between ONOO^–^ and ATP, probe **96** was developed in 2022, capable of concurrently monitoring changes in both ATP and ONOO^–^ concentration caused by acetaminophen (APAP)-induced hepatotoxicity.^169^ This dual rhodamine–naphthalimide probe initially exhibits only minimal fluorescence, but on exposure to ONOO^–^ its 4-BPin functionality is oxidized to the corresponding phenol to generate an ICT process, leading to a fluorescence turn-on response (λ_ex_ = 450/488 nm, λ_em_ = 562/568 nm). In the presence of ATP, the fluorescence intensity increases at 587 nm due to reversible noncovalent bonding (both π-stacking and H-bonding) to the rhodamine fluorophore, causing it to ring-open to its fluorescent form. Both fluorescent modes can be active at the same time, allowing the concurrent monitoring of increases in ONOO^–^ and depletion of ATP. Boasting good selectivity, pH stability, and low biotoxicity, probe **96** could be used to image APAP-induced injury with HL-7702 cells. This now-commercially available probe should provide an excellent tool for monitoring ATP and ONOO^–^ concentrations both in drug-induced liver injury (DILI) systems and in other diseases and/or organs.

Focusing on oxidative stress, in 2023 Zhang et al. developed a new NIR fluorescent probe **97** based on a thiol-chromone “click” reaction for visualizing thiol flux in DILI (see section 2.2 for ROS/thiol equilibrium).^170^ Probe **97** consists of a chromene-thiol recognition group and a dicyanoisophorone fluorophore to which an α,β-unsaturated ester was appended. On reaction with a thiol, a cascade of reactions is triggered, wherein probe **97** first loses its chromene moiety, producing an intermediate phenolic species that subsequently ring-closes onto the unsaturated ester to produce a coumarin bicycle. This has the benefit of not only producing a new absorption, centered at 540 nm, but also reduces the existing 400 nm absorption of the dicyanoisophorone part of probe **97**, while concurrently generating a new emission centered at 705 nm. An excellent linear relationship was observed between the fluorescence intensity of probe **97** and Cys concentration, with a good detection limit of 33 nM. Probe **97** is also able to detect Hcy and GSH, with slightly poorer detection limits of 100 nM and 40 nM, respectively. Under physiological conditions, probe **97** has excellent selectivity, rapid response to thiols, and can be used to visualize thiol fluctuations in cells and zebrafish, as well as monitor changes in thiol levels in DILI mice.

In 2022, Tang et al. developed a viscosity-activated NIR-II fluorescent probe **98** for the detection of lysosomal viscosity in hepatocytes and mice during hepatic ischemia–reperfusion injury (HIRI).^171^ Probe **98** combines structural features from both indocyanine green (ICG) and IR-783 to produce a red-shifted NIR-II fluorescence emission. Only a weak absorption at 694 nm occurs in water with low viscosity, and as the viscosity of the medium increases, this absorption band nearly disappears, with a concurrent increase in the absorption centered at 820 nm. An associated 13-fold increase in fluorescence emission intensity was observed at 864 nm on moving from 3.0 to 46.00 cP viscosity. The fluorescence intensity (log *F*_864_) exhibited a good linear relationship with the viscosity of the medium, with a linear coefficient of 0.997 and a quantum yield of 0.34 in glycerol (2.6 times higher than ICG). Probe **98** exhibited high specificity for viscosity, and polarity changes from different solvents had no effect on the fluorescence intensity of this probe. Using probe **98**, Tang et al. established a ROS-malondialdehyde-cathepsin B signaling pathway in HIRI.

In 2022, Ye et al. designed probe **99**, a multifunctional fluorescent probe for the simultaneous detection of lysosomal ATP and H_2_S in APAP DILI and HIRI.^172^ Rhodamine 6G and 1,8-naphthalimide hybrids were combined with diethylenetriamine and azide groups used as the ATP and H_2_S reactive moieties, respectively. In the presence of ATP, probe **99** exhibited a significant enhancement of fluorescence emission at 560 nm, caused by binding and ring opening, as discussed previously. On the other hand, in the presence of H_2_S, probe **99** exhibited a rapid increase in fluorescence emission centered at 530 nm, caused by reduction of the azide functionality. Probe **99** responded well to ATP at acidic pH (4.0–5.5), with a broader operating range for H_2_S detection (pH 4.0–8.0), and excellent selectivity for both analytes (LOD = 25.9 μM for ATP, 0.628 μM for H_2_S). Thanks to probe **99**, fluctuations in ATP and H_2_S concentrations were accurately measured in lysosomes during DILI and HIRI.

In 2022, Yuan and co-workers developed a novel NIR-II fluorescent scaffold by adding electron-rich end groups such as xanthene and benzopyran to either end of cyanine-type trimethine frameworks, developing probe **100**.^173^ The absorption and the fluorescence emission wavelengths of this probe were found to be within the NIR-II region (938/1001 nm, 956/1005 nm, 962/1015 nm, and 988/1058 nm). Probe **100** was shown to be capable of reversibly responding to both ROS and disulfide species. First, the probe is oxidized by HClO to generate *N*-oxides at the tertiary amino sites with no further rearrangement or degradation, leading to a decrease in absorption at 980 nm and emission at 1040 nm, and a new blue-shifted absorption band around 824 nm. Addition of a reducing reactive sulfur species returns the oxidized probe to its former state, regenerating the absorption at 980 nm and emission at 1040 nm and returning to a “NIR-II ON” state. Probe **100** was found to have excellent photostability and was capable of the reversible detection of HClO in oxidative microenvironments of acute inflammation and liver-injury/repair models.

Probe **101**, reported by Tang’s group in 2019, is capable of synergistic dual-selective recognition of O_2_^•–^ and ONOO^–^.^174^ Its structural design is based on caffeic acid, a potent recognition site for superoxide. On reaction with O_2_^•–^, the catechol group is oxidized to an *o*-benzoquinone, leading to a blue fluorescence emission. Cy5, a NIR fluorophore on the other side of the probe, reacts with ONOO^–^ to cleave the polymethine chain to quench the fluorescence of Cy5. By these reactions, ONOO^–^ switches off the NIR-based fluorescence; while superoxide switches on a blue fluorescence signal. This consequently enables the separate but simultaneous multichannel measurement of both ROS species. Excellent sensitivity, selectivity, as well as specificity for both O_2_^•–^ and ONOO^–^ were observed, with detection limits of 6.38 nM for O_2_^•–^ and 6.09 nM for ONOO^–^, with no interference between analytes. Interestingly, this probe enabled the reversible fluorescence imaging of O_2_^•–^, as reaction with an endogenous or added reducing agent regenerated the nonfluorescent catechol motif. Mitochondrial levels of O_2_^•–^ and ONOO^–^ were successfully monitored in HIRI models using probe **101**, leading to the discovery of a O_2_^•–^-ONOO^–^-arginase 1-mediated IR injury signaling pathway, as well as the adverse effects of arginase 1 nitration on IR injury, providing a potential new therapeutic target for treating HIRI.

**Figure 35:**
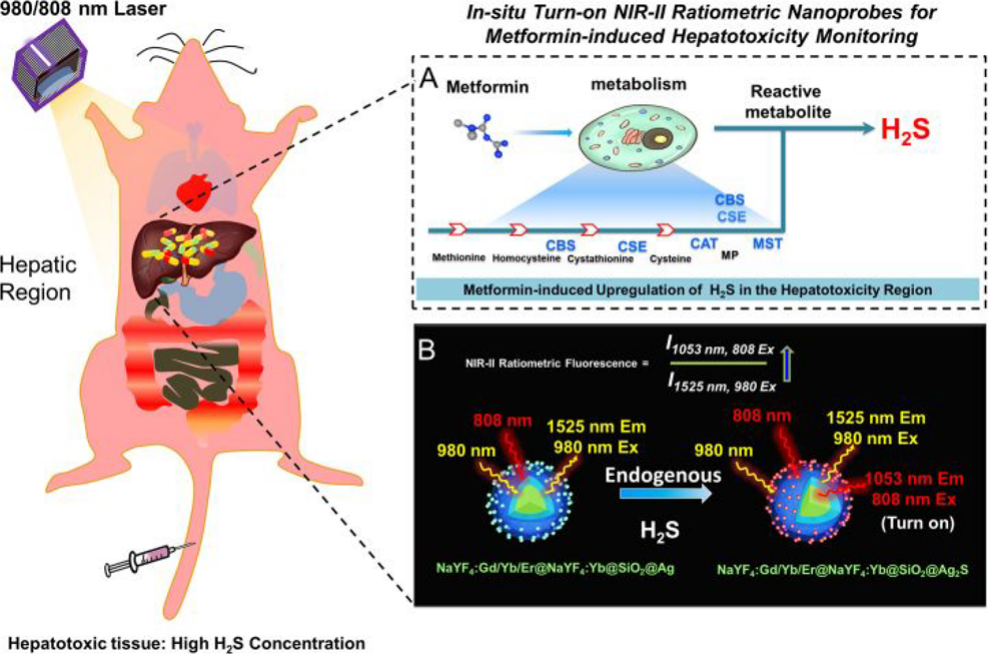
Schematic illustration of the visualization of H_2_S metformin-induced hepatotoxicity using probe **102**. Reproduced with permission from ref (175). Copyright 2021 American Chemical Society.

In 2021, Zeng developed a H_2_S-activated ratiometric nanoprobe NaYF_4_:Gd/Yb/Er@NaYF_4_:Yb@SiO_2_ (probe **102**) with efficient orthogonal NIR-II emission for *in situ* highly specific visualization of metformin-induced hepatotoxicity (Figure 35).^175^ It is constructed from a NaYF_4_:Gd/Yb/Er@NaYF_4_:Yb@SiO_2_ core, coated with Ag nanodots, forming a myrica rubra-like structure with a size of 75 nm. Probe **102** was mainly taken up by the liver and subsequently converted to NaYF_4_:Gd/Yb/Er@NaYF_4_:Yb@SiO_2_@Ag_2_S via *in situ* sulfuration triggered by overexpressed endogenous H_2_S in injured liver tissue, leading to a turn-on orthogonal emission centered at 1053 nm (808 nm laser irradiation) and 1525 nm (980 nm irradiation). Probe **102** exhibits excellent selectivity and specificity for H_2_S, with a 0.7 nM LOD. *In situ* highly specific ratiometric NIR imaging of metformin hepatotoxicity was successfully achieved using the activated orthogonal properties of probe **102**.

**Figure 36:**
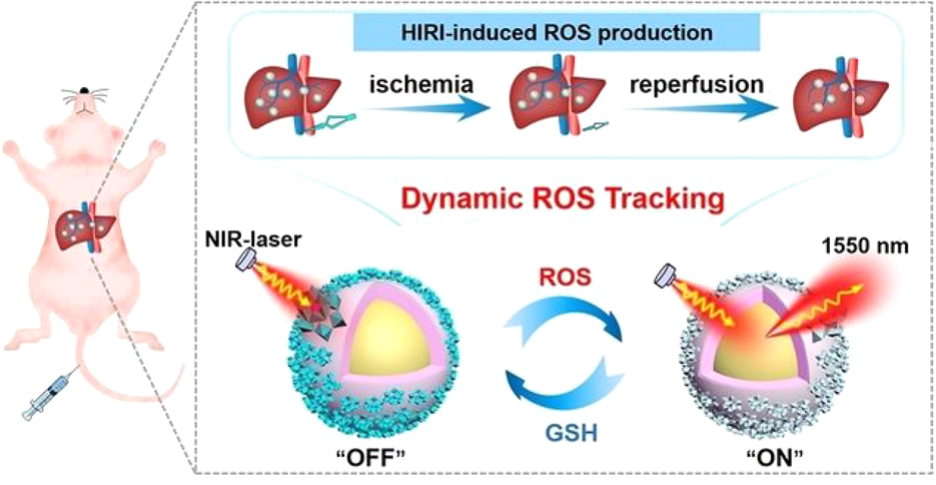
NIR-II Imaging of ROS in HIRI by probes **103**. Reproduced with permission from ref (176). Copyright 2022 John Wiley & Sons.

In 2022, Liu et al. developed reversible redox probes **103** composed of rare earth ions-doped nanoparticles (RENPs) and molybdenum-based polyoxometalate nanoclusters (Mo-POMs) for real-time imaging of ROS fluctuations in HIRI (Figure 36).^176^ Operating via the absorption competition-induced emission (ACIE) effect, these RENPs act as the luminescent moiety, while the Mo-POMs act as the competitor and ROS-recognition unit. The doped Nd and Yb allowed RENPs to produce a ratiometric luminescence signal when excited by 808 nm (Nd) and 980 nm (Yb) lasers, which benefits from minimal biological overlap. On oxidation of the Mo-POM layer (Mo^V^ to Mo^VI^) competition is reduced, and RENP centered emission increases. This can be reversed by exposure to a reducing agent such as GSH. Using this innovative platform, probes **103** were able to reversibly detect •OH and GSH, with a linear correlation between *F*_808_/*F*_980_ and concentration of •OH in the range from 1.0 to 9.0 μM with a detection limit of 0.46 μM, to successfully monitor ROS fluctuations during HIRI in real time.

**Figure 37:**
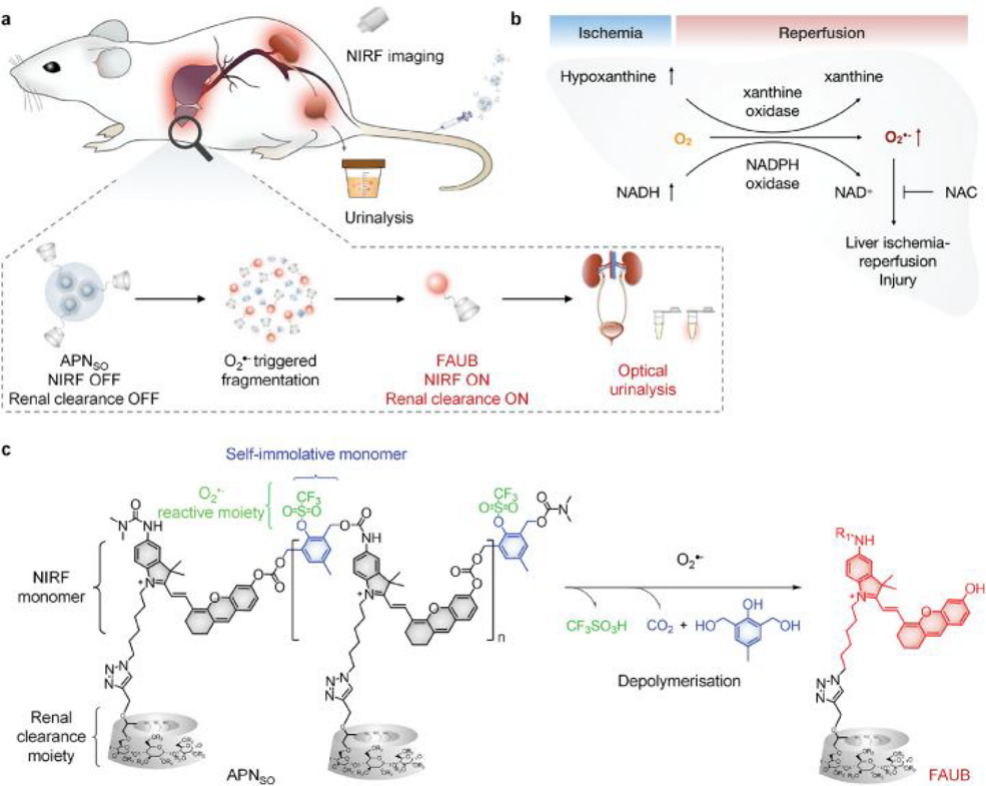
Design, synthesis, and mechanisms of probe **104** for NIRF imaging and urinalysis of HIRI. Reproduced with permission from ref (177). Copyright 2022 John Wiley & Sons.

In 2022, Wang and Pu et al. designed probe **104**, a polymer-based nanoprobe APN_SO_, with an O_2_^•–^-triggered NIR fluorescence response and kidney clearance switch for noninvasive *in vivo* imaging of HIRI and renal metabolism analysis (Figure 37).^177^ Probe **104** consists of four components: an O_2_^•–^-reactive unit, an autolysis unit, a caged fluorophore unit, and a renal clearance unit. The fluorophore unit is connected to the autolysis unit to form a polymeric backbone. This is then functionalized with O_2_^•–^-cleavable triflate groups and renal clearance hydroxypropyl β-cyclodextrin units. The amphiphilicity of the resulting polymers leads to spontaneous assembly into nanoparticles in aqueous media. In the absence of O_2_^•–^, probe **104** was nonfluorescent. However, in the presence of O_2_^•–^, the ROS cleaved the triflate, inducing a self-immolation to depolymerize the backbone of probe **104**, concomitantly releasing the kidney-clearable NIR fluorescent fragments termed fluorescent artificial urinary biomarkers (FAUBs). Thus, after accumulation in the liver following systemic administration, superoxide-induced cleavage releases FAUBs that can be used for real-time NIRF imaging and renal metabolism analysis.

**Figure 38:**
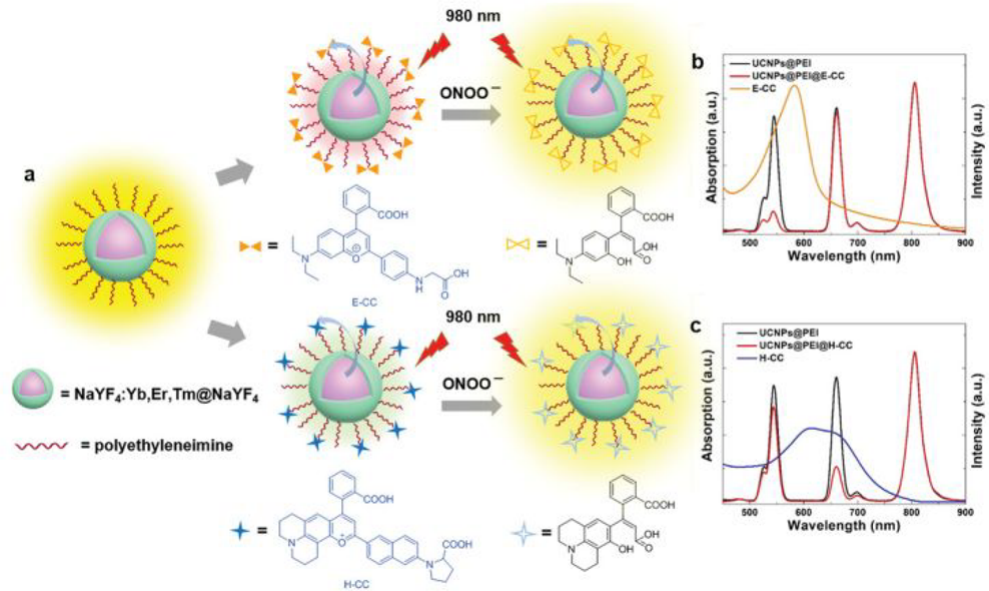
Ratiometric ONOO^–^ detection based on probe **105** and **106**. (a) Mechanism of action. (b,c) UV/vis spectra of probes containing chromophores E-CC (**105**) (b) and H-CC (**106**) (c) and upconversion emission spectra of UCNPs before and after modification under excitation at 980 nm. Reproduced with permission from ref (178). Copyright 2019 John Wiley & Sons.

In 2019, Yuan and Peng et al. designed two highly selective ratiometric fluorescent probes UCNPs@PEI@E-CC and UCNPs@PEI@H-CC (probe **105**, **106**) for tracking ONOO^–^ as a hepatitis indicator (Figure 38).^178^ Probe **105** and **106** consist of UCNPs and two novel pyrilium-based chromophores E-CC or H-CC. The upconversion luminescence of the nanoparticles (540 and 660 nm, 810 nm emission unchanged) is initially quenched by the chromophores, which are later oxidized and “destroyed” on exposure to ONOO^–^. This enables the successful ratiometric detection (*I*_540_/*I*_660_ or *I*_660_/*I*_810_) of ONOO^–^. Pleasingly, probes **105** and **106** could selectively detect ONOO^–^ over other dye-bleaching reactive molecules, such as HOCl and SO_3_^2–^, reducing the risk of interference from these competitors. Impressive nanomolar LODs were achieved, estimated at 154 nM for probe **105** and 241 nM probe **106**. These highly selective and sensitive probes were successfully used to image a novel CCl_4_-induced liver injury mouse model.

As for cancer, GGT is also a biomarker for drug-induced liver injury and plays an important role in the diagnosis of severe cases of DILI in clinical trials and clinical practice.^179^ In 2016, Wu and Zeng et al. developed the first TP fluorescent probe for visualizing GGT, probe **107**.^180^ A DCM-derived fluorophore was employed as the TP fluorogenic reporter, with glutamic acid acting as the ICT fluorescence quencher and recognition unit. On exposure to GGT, the glutamate unit is cleaved to release highly electron-donating aniline, affording a strong ICT-centered emission band at 635 nm. The detection of GGT by probe **107** reaches maximum fluorescence intensity after 30 min, and the probe has a good linear response to GGT in the range of 0–35 U/L, with a detection limit of 0.057 U/L. Probe **107** also had a good photostability and selectivity and has been successfully applied in the imaging of drug-induced liver injury in zebrafish.

### 4.2 Fluorescent Probes for Kidney Injury

Acute kidney injury (AKI), which results in a sudden loss of or severe decrease in kidney function, has become a global health issue due to its high incidence and mortality. AKI can be caused by sepsis, low blood pressure, organ failure, kidney stones, physical trauma, or overuse of medication. Prevention of severe life-changing or fatal AKI relies on accurate diagnosis, but unfortunately current clinical diagnostic methods rely mainly on the detection of serum creatinine and blood urea nitrogen, tests which are not sensitive enough for the early diagnosis of AKI. Highly sensitive, low-cost molecular optical imaging has exhibited promise for early AKI diagnosis, looking at biomarkers such as O_2_^•–^, ONOO^–^, HClO, and apoptosis-associated caspase-3.^181,182^

**Figure 39:**
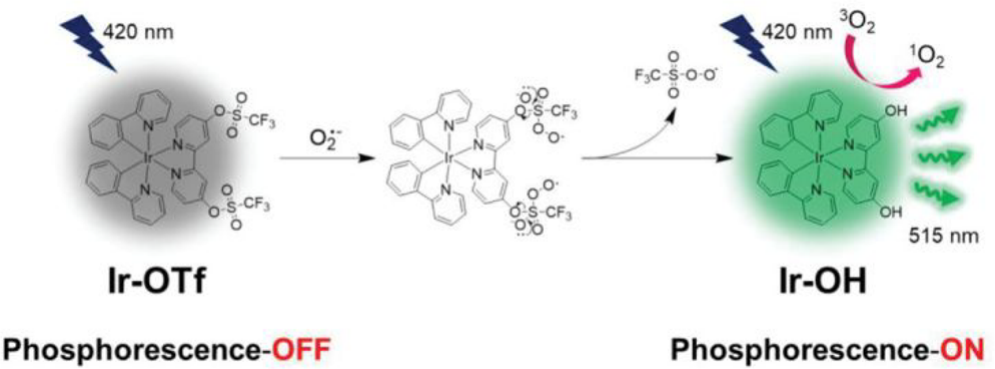
Molecular structure and O_2_^•–^-activated phosphorescence of IrOTf (probe **108**). Reproduced with permission from ref (183). Copyright 2022 John Wiley & Sons.

A simple and innovative multifunctional components-incorporated afterglow nanosensor (MANS), probe **108**, was developed by Kim and co-workers in 2022 for the superoxide-responsive activatable afterglow imaging of cisplatin-induced kidney injury (Figure 39).^183^ Fluorescent probe Ir-OTf is incorporated into a polymeric micelle nanoparticle alongside rubrene, which has a dual role as an afterglow substrate and luminophore. Probe **108** is ultimately a simple “off–on” activatable system, in which superoxide activates afterglow luminescence for long periods of time (>11 min). This is achieved by cleavage of the triflate from Ir-OTf, which activates the emission of the system. Probe **108** was successfully applied to molecular imaging of cisplatin-induced kidney injury in a mouse model, with afterglow capability enabling imaging of pathologically overproduced superoxide without any interference from autofluorescence.

**Figure 40:**
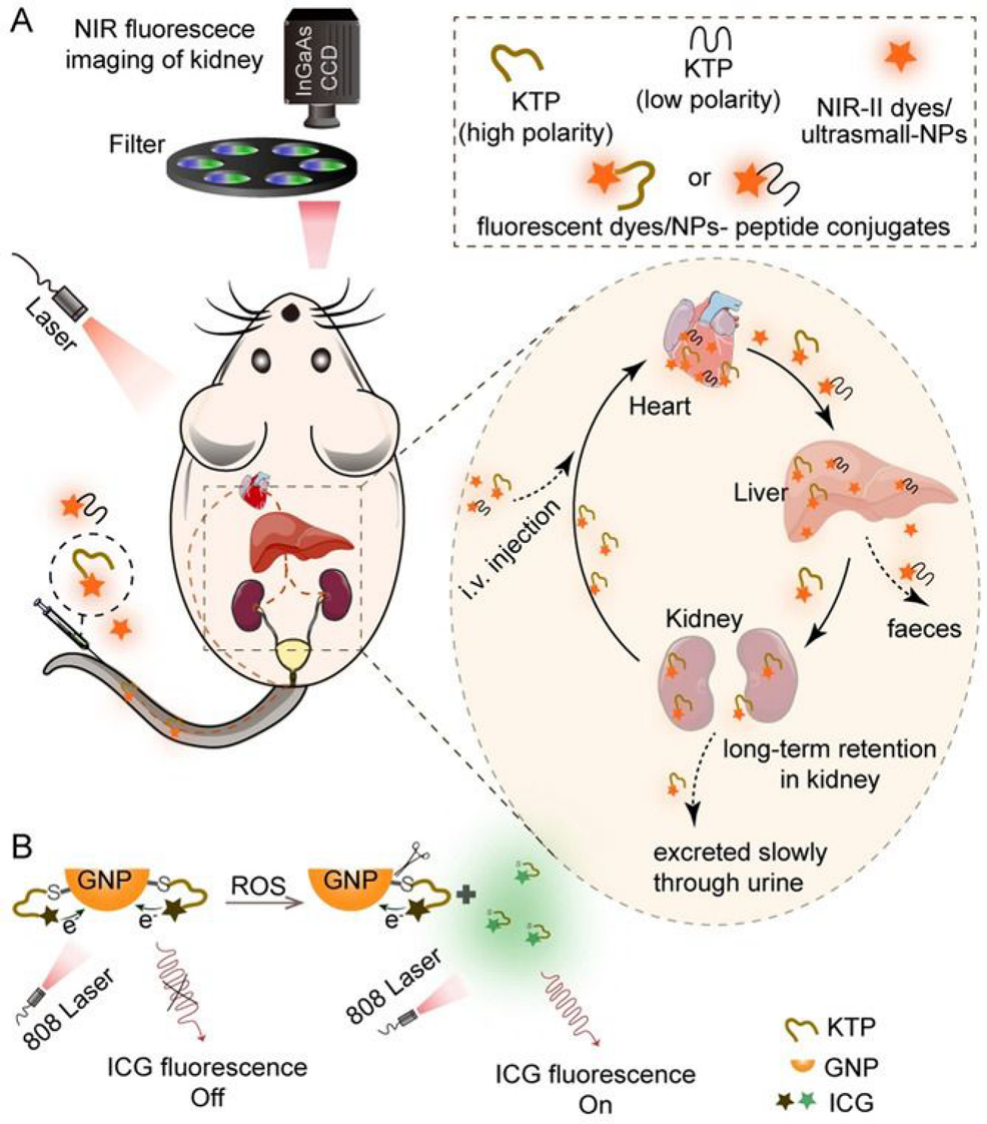
(A) Schematic depictions of the metabolic pathways of different dye-KTP conjugates *in vivo*, and noninvasive kidney monitoring in the NIR-II window. (B) Design of ROS-responsive probe **109** for the detection of renal dysfunction. Reproduced with permission from ref (184). Copyright 2021 John Wiley & Sons.

In 2021, Zhang et al. developed a strategy for the peptide-mediated delivery and long-term accumulation (>48 h) of NIR-II fluorophores in the kidney.^184^ They found that appending a hydrophilic polypeptide to small organic fluorescent molecules such as indocyanine green can significantly change the biological metabolic pathway of these compounds to reduce their capture, clearance, and destruction, thus enabling long-term targeted imaging of the kidney. Building on this, Zhang and co-workers designed ROS-activated renal targeting nanoprobe GNP-KTPs-ICG (probe **109**). Probe **109** composed of ICG, gold nanoparticles (GNPs), and kidney-targeting peptides (KTP) (Figure 40). Probe **109** could build up in the kidney, reacting with ROS to detach the fluorescent ICG-KTP motif from the GNP to trigger a fluorescent signal for NIR-II imaging of kidney damage *in vitro* or *in vivo*.

**Figure 41:**
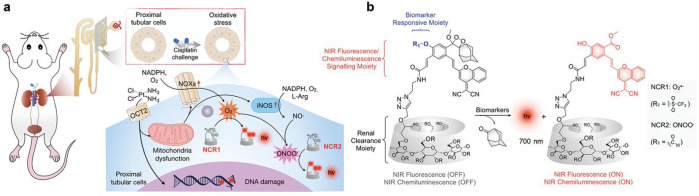
(a) The overall mechanism of action for the two probes, **110** and **111**. (b) The chemical structures of **110** and **111** for the detection of O_2_^•–^ and ONOO^–^ in AKI. R = H or CHO. Reproduced with permission from ref (185). Copyright 2020 John Wiley & Sons.

Two NIR chemiluminescent reporters (NCRs) probes **110** and **111**, with high renal clearance for real-time imaging of ROS and RNS in the kidneys were synthesized by the Pu group in 2020 (Figure 41).^185^ These NCRs comprise of a β-cyclodextrin clearance unit, and a chemiluminescent modified DCM containing Schaap’s dioxetane. Two NCRs were reported (probe **110**, NCR1 for O_2_^•–^, probe **111**, NCR2 for ONOO^–^), each with a different biomarker-specific reactive unit on the luminophore (triflate and aldehyde, respectively). With nanomolar sensitivity and high renal clearance, these NCRs could detect minor cellular concentration changes in ROS and RNS concentration and were used to monitor biomarker levels in nephrotic kidneys. These studies indicated that probe **110** is activated earlier than probe **111**, implying that O_2_^•–^ and ONOO^–^ are upregulated sequentially, with the former increasing first in AKI. Furthermore, detection of secreted fluorescent NCRs can allow urinalysis of AKIs to be carried out over 24 h earlier than comparable histological analysis.

**Figure 42:**
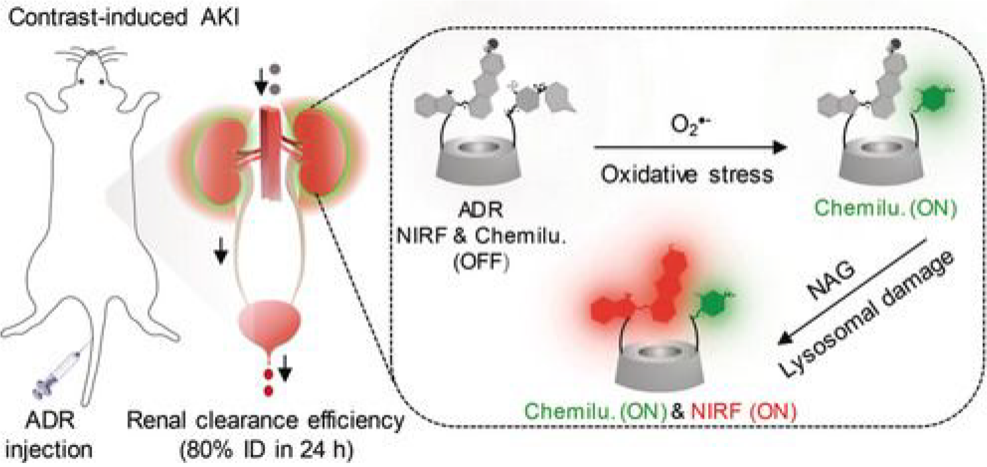
An optical probe **112** with turn-on chemiluminescence and NIR fluorescence for detecting O_2_^•–^ and NAG. Reproduced with permission from ref (186). Copyright 2019 John Wiley & Sons.

Contrast media-induced acute kidney injury (CIAKI) is a medical complication characterized by deterioration of renal function following the use of contrast media. In 2019, Pu et al. designed a dual probe **112** for real-time imaging of CIAKI *in vivo* through imaging of oxidative stress (superoxide anion) and lysosomal damage (*N*-acetyl-β-d-glucosaminidase, NAG) (Figure 42).^186^ Probe **112** has two signaling channels, chemiluminescence and NIR fluorescence. The former is activated by O_2_^•–^, while the latter is activated by NAG. Probe **112** has high renal clearance (up to 80%), and so it enables detection of elevated O_2_^•–^ and NAG in the kidneys of living mice to diagnose CIAKI well before glomerular filtration rate decreases or tissue damage can be observed using conventional tests. This potentially renders probe **112** more effective than current clinical detection methods, and so it is hoped that it could provide a new tool for real-time noninvasive monitoring of renal function in an acute clinical setting.

**Figure 43:**
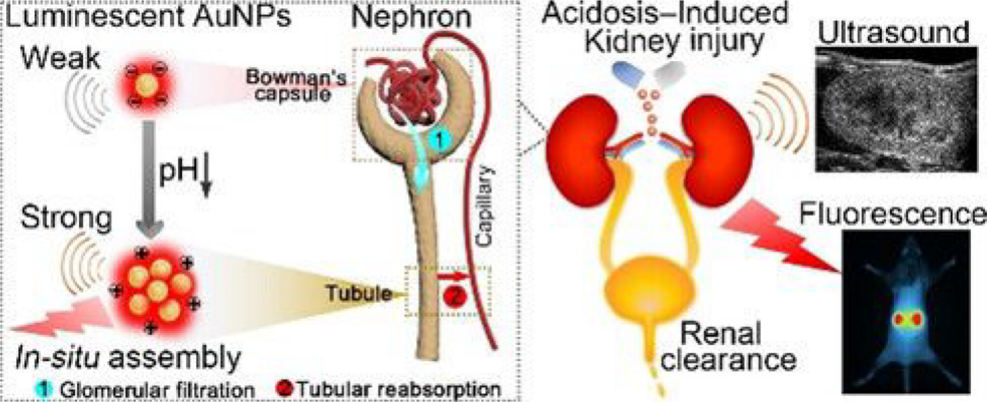
Probe **113** with pH-induced charge reversal and aggregation properties for synergetic fluorescence and ultrasound diagnosis of early kidney injury, enabled by reabsorption and *in situ* aggregation in tubular cells of injured kidneys. Reproduced with permission from ref (188). Copyright 2021 John Wiley & Sons.

Nanoparticles are known to generally be rapidly cleared by glomerular filtration, and so their interaction with and residence time in the kidney, necessary for accurate sensing, is often very low.^187^ This, combined with a generally poor ultrasonic signal, poses significant challenges for the direct imaging and diagnosis of kidney diseases using nanoparticle-based platforms. Liu and Wu have reported some progress on this issues, developing in 2021 a new class of ultrasmall, renal-cleared luminescent gold nanoparticles coated with pH-responsive zwitterionic imidazole groups (PMIZ-AuNPs, probe **113**) with pH-induced charge reversal and aggregation properties (Figure 43).^188^ Probe **113** has a size of 3.5 nm at pH 7.4 and 1048 nm at pH 5.5, indicating significant aggregation through hydrogen bonding in acidic media, greatly enhancing the ultrasonic signal. Thus, after clearing through glomerular filtration, probe **113** is forced to aggregate in the acidic environment of renal tubules, increasing reabsorption, retention, as well as fluorescence and ultrasound signals, enabling more effective imaging of kidney damage than previously possible using nanoprobes. This study suggests that modulating the *in vivo* clearance pathway of the luminescent probe **113** could be a fluoro-ultrasound collaborative imaging strategy that could be used in future to diagnose early kidney injury and obtain precise anatomical information.

**Figure 44:**
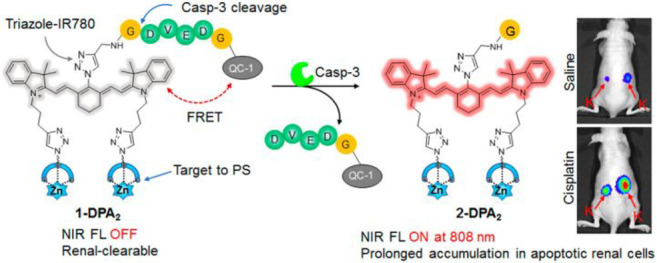
Caspase-3-mediated hydrolysis of probe **114** into fluorescent product 2-DPA_2_. Reproduced with permission from ref (189). Copyright 2021 American Chemical Society.

In 2021, the Ye group designed and synthesized phosphatidylserine (PS)-targeting and caspase-3-activated NIR fluorescent probe 1-DPA_2_ (probe **114**) using a “one-pot sequential click response” approach, demonstrating their ability to perform noninvasive and real-time imaging of kidney cells in the early stages of drug-induced AKI (Figure 44).^189^ Probe **114** consists of a triazole-substituted IR780 fluorophore, a caspase-3-cleavable peptide (G-DEVD-G), two PS-targeting ligands (DPA-Zn), and a NIR fluorescence quencher (QC-1). In the absence of caspase-3, NIR fluorescence was quenched by the close proximity of QC-1 and IR780. On addition of caspase-3, emission at 808 nm increased due to cleavage of the DEVD linker, doing so with good linearity relative to caspase-3 concentrations in the 0.01–0.2 μg/mL range. Using probe **114**, fluorescence imaging showed accumulation primarily in the kidneys of mice, enabling the monitoring of caspase-3 activity during early AKI processes of mice stimulated by cisplatin. The recovery of AKI mice after treatment with *N*-acetyl-l-cysteine can also be monitored.

**Figure 45:**
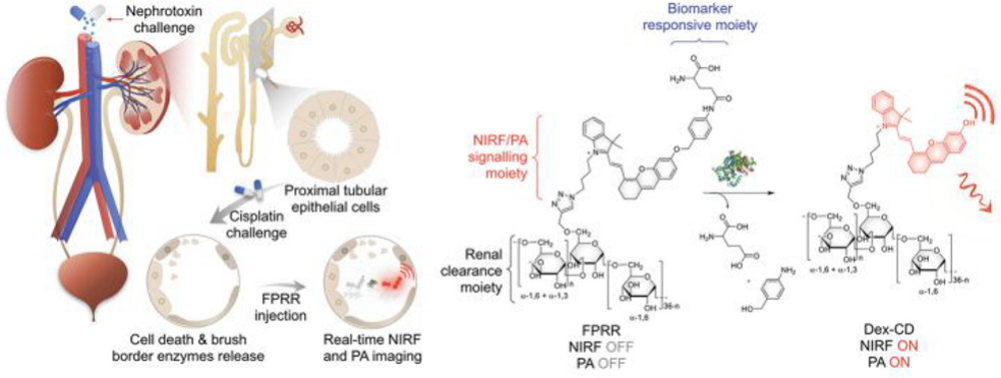
Schematic illustration and molecular mechanism of probe **115** for real-time NIRF and PA imaging of AKI. Reproduced with permission from ref (190). Copyright 2020 John Wiley & Sons.

In 2020, the Pu group synthesized another fluoro-photoacoustic polymeric renal reporter probe FPRR (probe **115**) for real-time imaging of drug-induced AKI (Figure 45).^190^ Probe **115** consists of three moieties: a polymeric dextran renal clearance enabler (*vide supra*), a NIRF/PA signaling hemicyanine moiety, and a biomarker-responsive moiety (γ-glutamate). As in similar examples above, the hydroxyl group of hemicyanine was masked with the GGT-cleavable γ-glutamate moiety, suppressing fluorescence. On exposure to GGT, cleavage and elimination are triggered, leading to enhancement in both NIR fluorescence and photoacoustic signal. This study not only demonstrated the first activated PA probe for sensitive imaging of renal function in real time at the molecular level, but also further highlights the application of polymer-based probes with high renal clearance.

**Figure 46:**
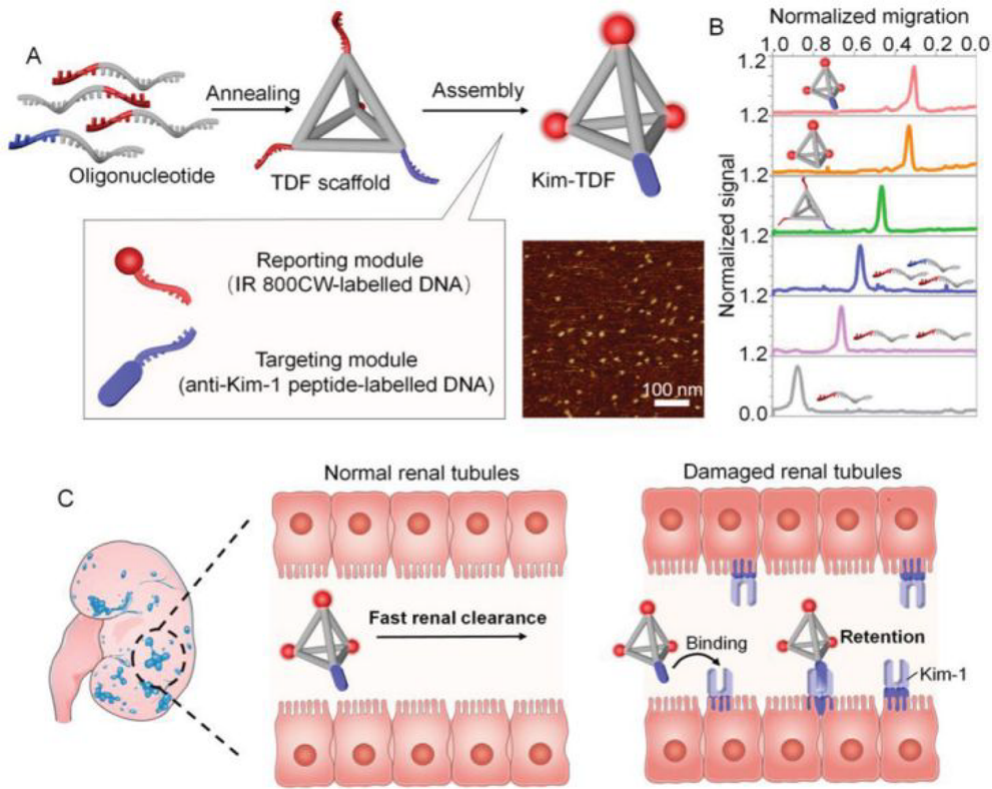
(A) Synthesis of probe **116**. (B) Characterization of probe **116** by native PAGE gel electrophoresis. (C) Schematic illustration of fluorescence imaging of Kim-1 using probe **116**. Reproduced with permission from ref (191). Copyright 2022 John Wiley & Sons.

In 2022, Xia and co-workers reported a tetrahedral DNA framework (TDF)-based nanodevice Kim-TDF (probe **116**) for *in vivo* NIR imaging of early AKI (Figure 46).^191^ Probe **116** again consists of three functional modules: a size-tunable TDF nanostructure as a kidney-targeting support, a binding module for biomarker kidney injury molecule-1 (Kim-1), and a commercial NIR signaling module (IR CW800). Probe **116** selectively accumulates in damaged kidney tissues with high Kim-1 levels, to subsequently produce a strong near-infrared fluorescence output. Appropriately sized nanodevices are quickly cleared in healthy kidneys, thus minimizing background signals. Early diagnosis of developing AKI using probe **116** was demonstrated a full 6 h prior to successful Kim-1 urine analysis, and 12 h before successful blood urea nitrogen testing.

**Figure 47:**
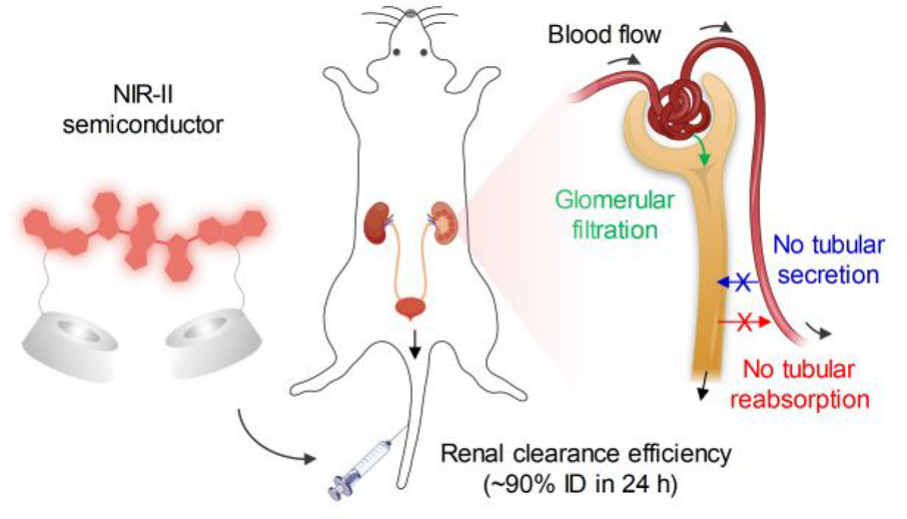
Probe **117** for real-time NIR-II fluorescence imaging of kidney dysfunction. Reproduced with permission from ref (192). Copyright 2019 John Wiley & Sons.

In 2019, the Pu group also synthesized a NIR-II fluorescent molecular semiconductor CDIR2 (probe **117**) for real-time imaging of kidney dysfunction in living mice (Figure 47).^192^ Probe **117** comprises two parts: a complex NIR-II fluorophore, and a renal-clearance-enabling moiety ((2-hydroxypropyl)-β-cyclodextrin). The NIR-II fluorophore possesses a typical “shielding unit–donor–acceptor–donor–shielding unit” structure with benzo[1,2-*c*:4,5-*c*′]-*bis*-([1,2,5] thiadiazole), 3,4-ethoxylenedioxy-thiophene, and dialkyl fluorene as the acceptor, donor, and shielding units, respectively. After systemic administration in living mice, probe **117** was cleared through glomerular filtration without reabsorption and secreted into renal tubules. Probe **117** exhibits a range of advantages over other probes, with a high signal-to-background ratio, a very high renal clearance efficiency (about 90% after 24 h), and minimal *in vivo* metabolism, making this platform a promising candidate for noninvasive monitoring of kidney dysfunction, and for the development of other renal probes.

**Figure 48:**
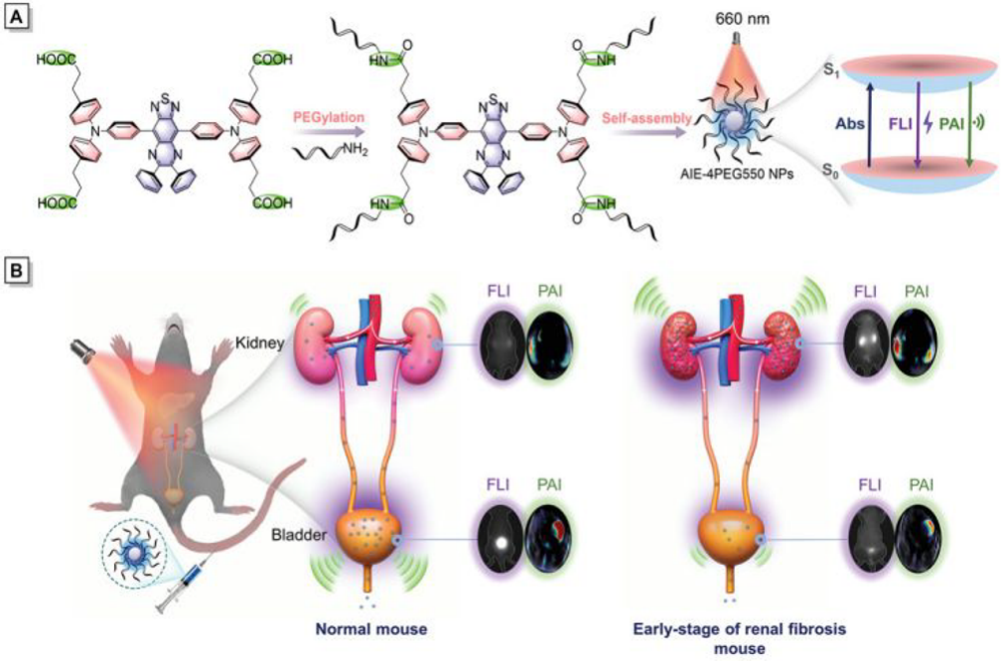
(A) Design and photophysical processes of self-assembled ultrasmall probe **118**. (B) Use of probe **118** for bimodal imaging the progress of renal fibrosis in mice. Reproduced with permission from ref (193). Copyright 2022 John Wiley & Sons.

In 2022, Tang and collaborators developed NIR AIE probe AIE-4PEG550 NPs (probe **118**), a water-soluble system for the diagnosis of mouse kidney fibrosis using dual-mode fluorescence and photoacoustic imaging (Figure 48).^193^ Probe **118** is an electron donor–acceptor–donor (D-A-D)-type AIEgen with improved water solubility from PEGylation with 0.55 kDa PEG-NH_2_. This water-soluble NIR fluorophore has good photostability and biocompatibility and could clearly identify early fibrosis. This probe’s small (≈26 nm), renally filtrable molecular weight (3.3 kDa), high renal clearance efficiency (93% within 24 h), outstanding imaging performance, and good biocompatibility make probe **118** a clear candidate for the future development of clinical diagnostic assays.

**Figure 49:**
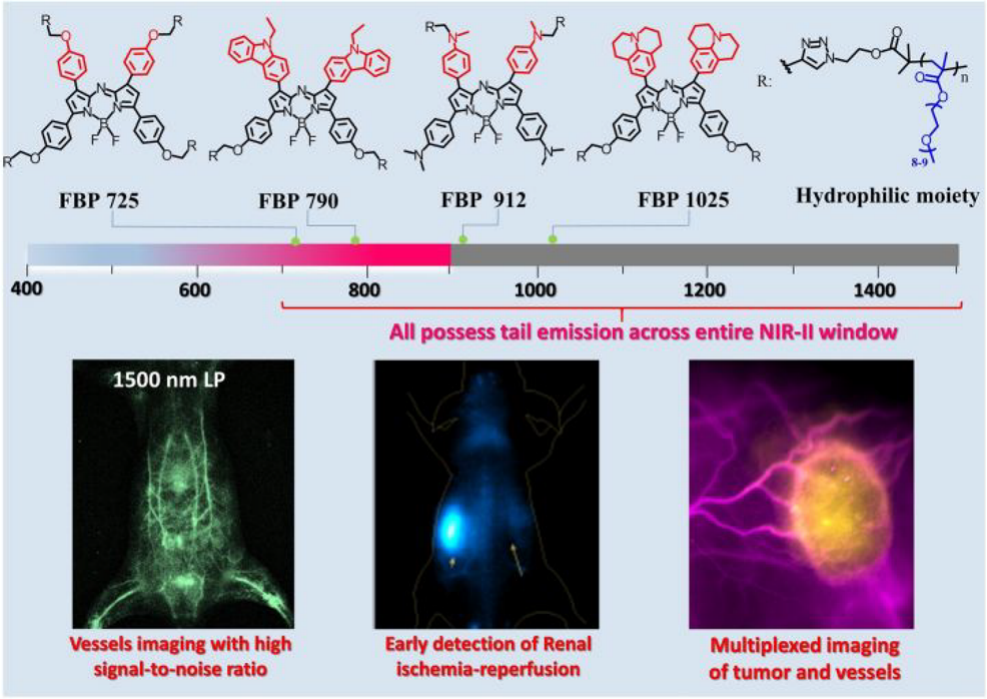
NIR-II brush macromolecular fluorophores. Reproduced with permission from ref (194). Copyright 2021 John Wiley & Sons.

The final example to discuss in the context of kidney damage is based around the series of brush-like fluorescent probes (Figure 49) developed by the Zhang group in 2022. Based on *aza*-BODIPY, these probes, such as FBP912 or **119**, are readily cleared by the kidney and have adjustable emission wavelengths ranging from 725 to 1025 nm depending on which substituents are used (Figure 49).^194^ Probes **119** are prepared by atom transfer radical polymerization (ATRP) between *aza*-BODIPY-based monomers and oligo(ethylene glycol) dimethacrylate to produce water-soluble probes that exhibit extended circulation time (*t*^1/2^ > 6 h). Optimized probe **119** boasts a NIR-II fluorescence intensity over 10 times that of any previously reported clearable NIR-II probe, as well a 65% 12 h clearance rate, enabling rapid, highly sensitive detection of HIRI.

### 4.3 Fluorescent Probes for Traumatic Brain Injury

Traumatic brain injury (TBI), often caused by extended lack of blood flow or violent trauma, is a significant cause of mortality and disability, often leading to varying degrees of brain damage, resulting in sensory, motor, cognitive, behavioral, psychological, and other functional impairment.^195^ As secondary injuries from traumatic brain injury, such as inflammation, BBB disruption, oxidative stress, hypoxia, and ischemia, are often invisible at first, approaches to achieve real-time early diagnosis and treatment of TBI are needed.

CT and MRI techniques that are typically relied upon for TBI diagnosis primarily detect anatomical and functional changes, and hence are of little use for detecting the early molecular fluctuations that underwrite TBI. There is therefore an urgent need to develop highly sensitive methods for early *in situ* real-time diagnosis and therapeutic evaluation of TBI, an issue currently being addressed in part by the fluorescent probe research community.^196,197^

**Figure 50:**
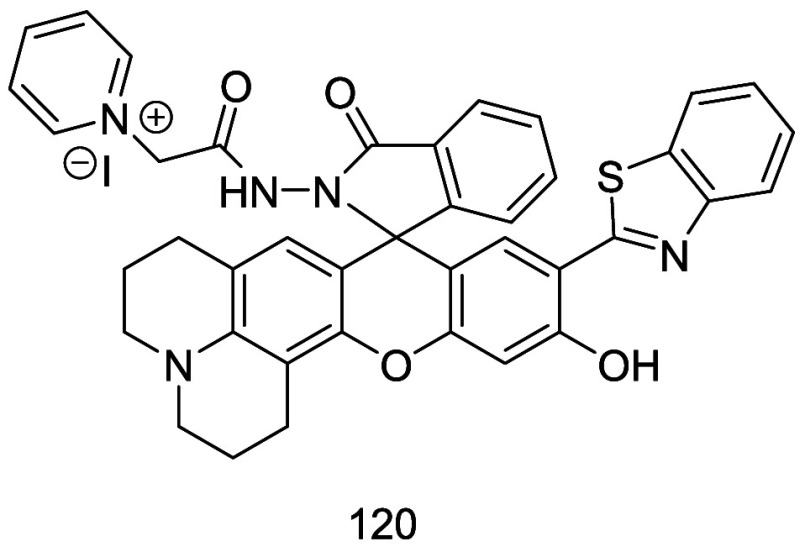
Probe **120** for the mitochondrial imaging of hypochlorous acid in traumatic brain injury.

Mitochondrial hypochlorous acid (HOCl) is closely related to the redox balance in mitochondria (*vide supra*), and abnormal levels of HOCl can induce mitochondrial inactivation and apoptosis. In 2020, Liu et al. published a ratiometric two-photon fluorescence probe **120** combining an ESIPT benzothiazole and rhodol (Figure 50).^198^ In this probe, a modified rhodol dye acts as the fluorophore, dihydrazide is the responsive group, and the cationic quaternized pyridinium serves as the mitochondria-targeting group. On addition of NaOCl, the fluorescence intensity at 453 nm changed very little, while that at 595 nm increased, enabling ratiometric fluorescence imaging of NaOCl. The ratio of two emission intensities (*I*_595_/*I*_453_) was linearly proportional to the NaOCl concentrations up to 174 μM, with a calculated LOD of 55.4 nM. Probe **120** exhibited excellent properties, including a fast response time, high sensitivity, high selectivity, and deep tissue penetration (270 μm). This probe could be used to monitor endogenous HOCl in living cells and tissues with TBI using dual emission channels and two-photon fluorescence microscopy.

**Figure 51:**
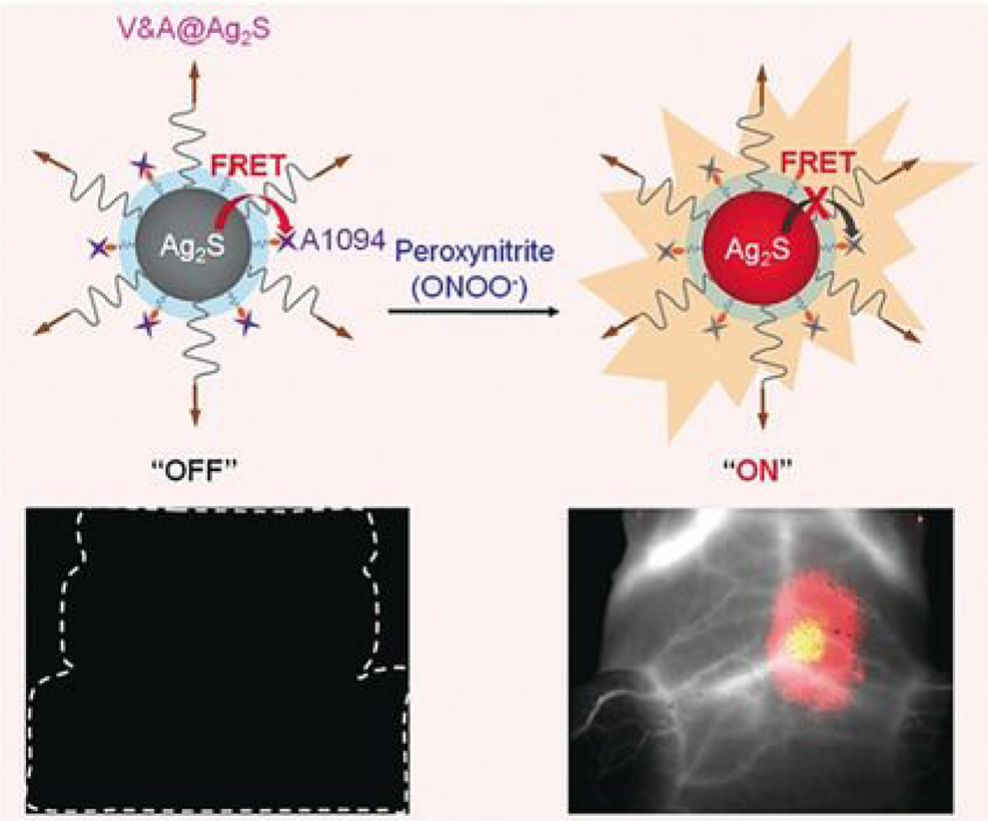
Probe **121** for visualizing TBI regions ONOO^–^-triggered fluorescence response. Reproduced with permission from ref (199). Copyright 2020 John Wiley & Sons.

In 2020, the Li group developed a NIR-II nanoprobe V&A@Ag_2_S (probe **121**) for real-time *in vivo* imaging of the early biomarkers of TBI (Figure 51).^199^ Ag_2_S quantum dots typically have a broad absorption profile and sharp NIR-II emission spectra, and after coupling NIR absorber A1094, a strong absorption peak appears at 1094 nm, with broad overlap with the Ag_2_S emission window, ensuring FRET between the QDs and A1094. This leads to fluorescence quenching, and a resting “off” state. Probe **121** has good specificity for ONOO^–^, showing a good linear relationship of the fluorescence intensity at 1050 nm vs the concentration of ONOO^–^, allowing for successful application of probe **121** to visualize endogenous ONOO^–^ in a mouse model of TBI.

**Figure 52:**
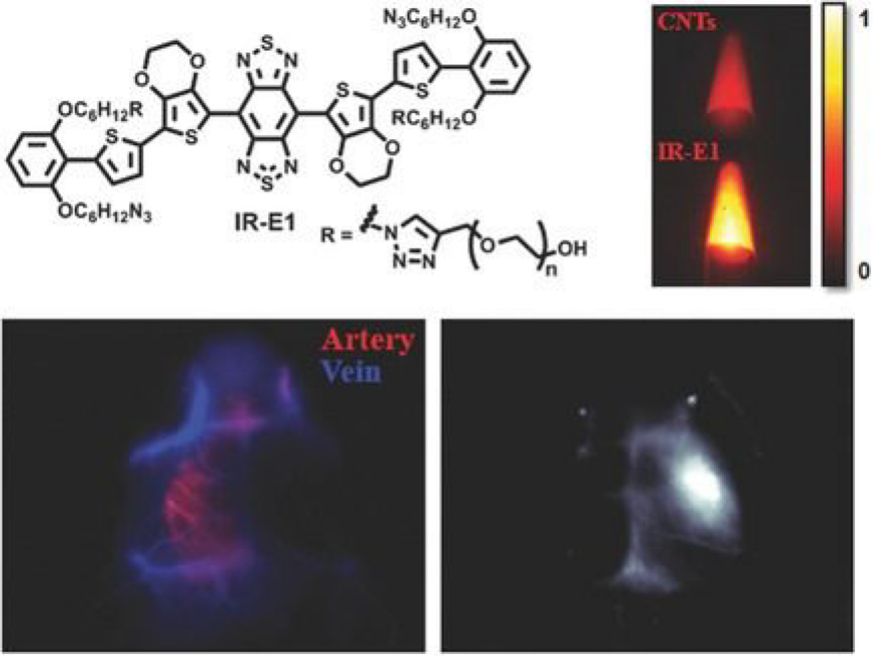
Probe **122** for noninvasive assessment of TBI. Reproduced with permission from ref (200). Copyright 2016 John Wiley & Sons.

In 2016, Dai et al. designed a novel NIR-II fluorescent dye for brain imaging in mice with TBI, whose structure mimics larger OLED-type luminophores (Figure 52).^200^ Their probe, termed IR-E1 (probe **122**), is built around a classical donor–acceptor–donor structure, with benzo[1,2-*c*:4,5-*c*′]-*bis*-([1,2,5] thiadiazole) as the acceptor and thiophene-based units as the donors to afford a fluorophore with a narrow band gap. Bulky 3,4-ethoxylene dioxythiophene acts as a bridging group to protect the conjugated backbone from intermolecular and intramolecular interaction. To enhance the water solubility of probe **122**, PEG chains were introduced into the structure. Under excitation at 808 nm, the probe emitted at 1071 nm in water, PBS, and fetal bovine serum, indicating good solvent and medium stability. Probe **122** was capable of visualizing dynamic vascular changes in mouse TBI models, including initial transient hypoperfusion. Brain imaging studies suggest that this pathology could be used as a biomarker in drug trials or in the clinic, and as a target for therapeutic invention.

TBI is a leading cause of death and disability in children and young adults, but there are currently no treatments to prevent secondary progression of the injury beyond the initial injury. The chronic development of this secondary damage is caused in part by the release of ROS into the surrounding normal brain tissue.

**Figure 53:**
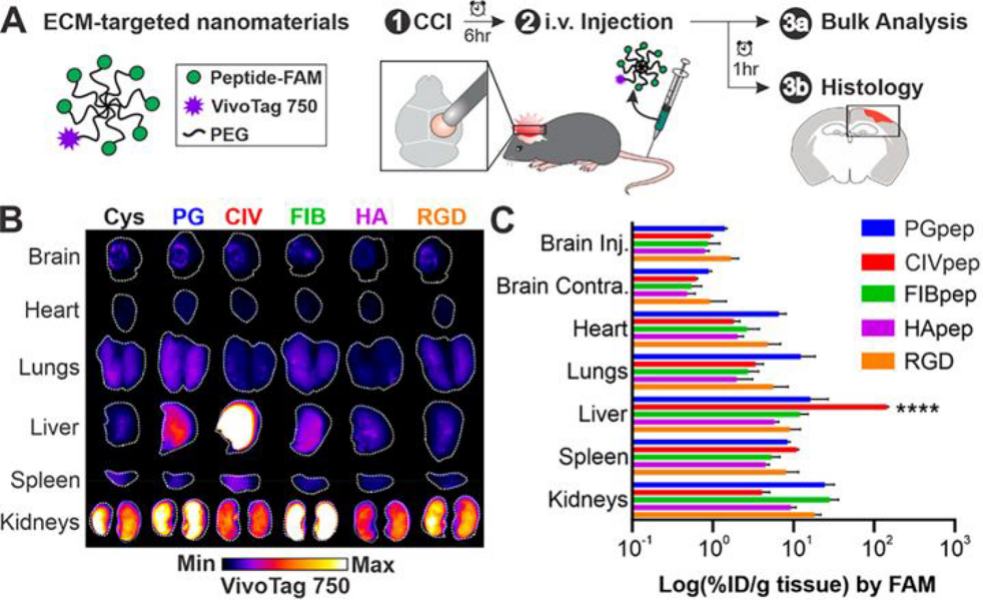
(A) Schematic of ECM-targeting probe **123** and overview of experimental design. (B) VivoTag 750 surface imaging of major organs. (C) Bulk quantification of percent injected dose of nanomaterial per gram of tissue (% ID/g) based on FAM fluorescence (*n* = 3, mean ± SEM; ****, *p* ≤ 0.0001, two-way ANOVA, and Tukey’s multiple comparisons post hoc test within each organ group). Reproduced with permission from ref (201). Copyright 2017 American Chemical Society.

Another biomarker for TBI is changes in the activity of ectopic proteases, as they mediate cell death, extracellular matrix breakdown, and inflammation. In 2021, Kwon and co-workers developed a fluorescent activity-based nanosensor (probe **123**) that accumulates in injured blood vessels in damaged regions of the brain and generates a fluorescent signal in response to calpin-1 lysis,^201^ thereby enabling visualization of TBI-associated calpin-1 protease activity (Figure 53) in real time. To improve the delivery and bioavailability of probe **123** stroma-targeting peptides were evaluated, showing that peptides targeting hyaluronic acid were better distributed in damaged brain tissue, in particular near lesions and in the hippocampal neurons. The hyaluronic acid-targeting peptide-coated probe showed an increase in activation in a ligand valency-dependent way, with up to a 6.6-fold increase in the damaged cortex compared to nontargeted nanosensors.

**Figure 54:**
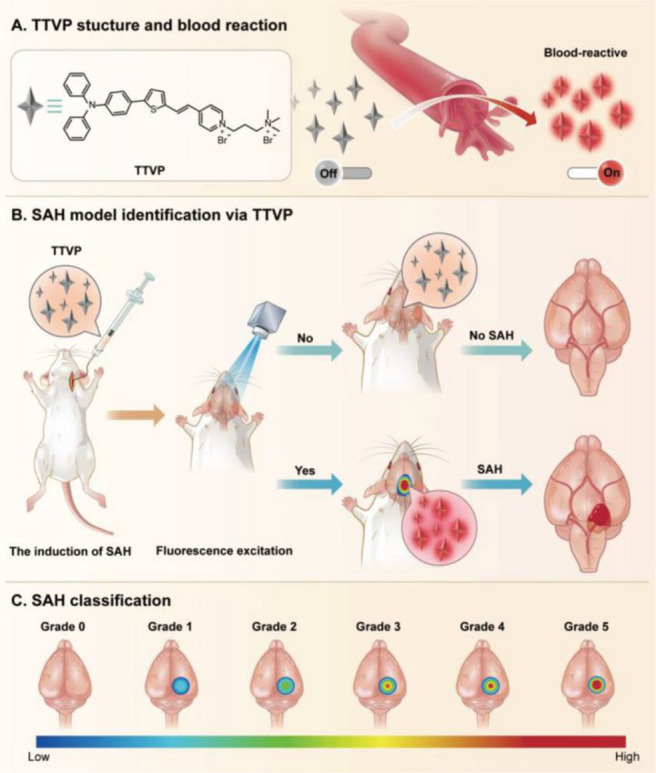
Schematic illustration of the use of probe **124** for SAH detection and classification. (A) Structure of probe **124** and its reaction to blood. (B) Use of probe **124** in mouse model. (C) SAH classification based on the intensity of the fluorescence observed in the brain. Reproduced with permission from ref (202). Copyright 2021 John Wiley & Sons.

Subarachnoid hemorrhage is a severe subtype of stroke caused by a ruptured blood vessel in the brain. Our ability to accurately assess the extent of bleeding in SAH models is critical to understanding brain injury mechanisms and developing treatment strategies. In 2023, the Dai group developed a new bleeding assessment system using a bioprobe TTVP based on AIE (Figure 54, probe **124**).^202^ Probe **124** consists of a prototypical 4-bromo-*N*,*N*-diphenylaniline AIE unit and pyridinium groups, with λ_ex_ = 480 nm, and λ_em_ = 645 nm. Due to the high degree of rotation of its molecular rotor-like structure, probe **124** hardly emits any fluorescence in aqueous solution. By causing the formation of nanoaggregates by addition of tetrahydrofuran, a gradual increase in the photoluminescence could be induced. Using these AIE properties, cell membrane affinity, and albumin targeting capabilities, probe **124** can be used to fluoresce specifically in areas where bleeding is occurring with a high signal-to-noise ratio. Probe **124** was successfully used to detect subarachnoid hemorrhages in the brain of mice, and it is expected to be a promising tool for the sensitive analysis of the bleeding degree of SAH and other hemorrhagic diseases.

**Figure 55:**
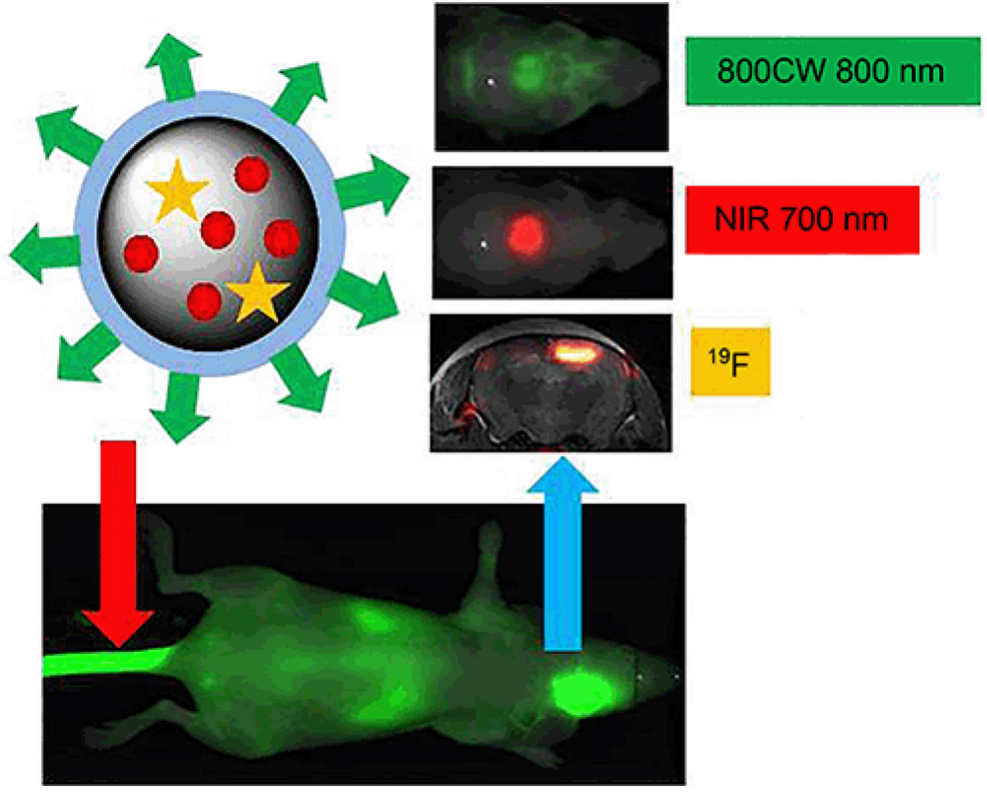
Schematic diagram of bimodal (NIR + ^19^F MRI) probe **125** (NIR700 + PFCE) targeted toward dead cells with an 800CW ligand. Reproduced with permission from ref (203). Copyright 2016 Springer Nature.

Another downstream effect of TBI is death of the tissue, or necrosis. In 2016, Löwik, Cruz, and co-workers reported a bimodal approach for the detection and monitoring of necrosis in TBI using PEGylated poly(lactic-*co*-glycolic acid, PLGA) nanoparticles (Figure 55, probe **125**).^203^ The surface of probe **125** was coated with a PEG–lipid layer to reduce nonspecific binding and allow the binding of specific ligands to target only necrotic areas. Cyanine dyes such as IRDye 800CW are encapsulated within in the probe, acting as both the fluorophore and the targeting group for intracellular proteins of cells that have lost membrane integrity. To enable tandem optical and ^19^F MRI imaging of necrosis in TBI, the authors introduced perfluorocarbons and NIR700 into probe **125**, with both modalities enabling accurate detection of necrosis, although greater sensitivity can be achieved by the optical imaging method. Using probe **125**, both rapid qualitative optical monitoring of the TBI state and quantitative 3D MRI analysis of deeper tissues could be performed to assess the extent of the lesion, demonstrating the great potential of this necrosis-targeting probe **125** for potential clinical applications in the diagnosis of brain injuries.

## 5 Fluorescent Probes for Cardiovascular Diseases

Cardiovascular disease (CVD) is recognized as a major cause of death and disability worldwide, with an estimated 17.9 million CVD-related deaths in 2019, representing 32% of all global deaths. Of these deaths, 85% were due to heart attack or stroke.^204^ The ability to detect abnormal biomolecular trends in cardiovascular diseases in order to investigate the molecular mechanisms of CVD is therefore crucial, as it should enable the further development of effective diagnostic, preventative, and therapeutic methods. In this section, we will look at representative examples of fluorescent probes developed for the imaging and diagnosis of key cardiovascular diseases such as myocardial ischemia/reperfusion injury, atherosclerosis, drug-induced cardiotoxicity, and hypertension (Table 4).

**Table 4:** Selected Fluorescent Probes for Cardiovascular Disease

cardiovascular diseases	probe	λ_ex_/λ_em_ (nm)	LOD	bioactive molecule	biological model	ref
MI/RI	126, 127	323/470, 385/510	800 nM, 10 nM	O_2_^•–^	A549 cells, H9C2 cells, HepG2 cells, C57BL/6 mice	(206)
OGD/R	128	390/525	90 nM	ONOO^–^	H9C2 cells	(208)
I/RI	129	530/560, 670/725	0.085 μM	ONOO^–^	H9C2 cells, HUVECs cells, SD rats	(209)
MI/RI	130	420/600	256, 200 nM	HOBr	H9C2 cells	(210)
myocardial hypoxia injury	131	560/		NO	H9C2 cells, HCASMC, Kunming male mice	(214)
atherosclerosis	132	365/580		ONOO^–^	RAW 264.7 cells, C57BL/6 mice, Ldlr–/– mice	(215)
atherosclerosis	133		72.6 ng/mL	ONOO^–^	A549 cells, RAW 264.7 cells, C57BL/6 mice	(217)
atherosclerosis	134	651/725, 488/575	0.014 U/mL	β-Gal	VSMCs, male C57BL/6 mice	(218)
atherosclerosis	135	458/520		LDs	A549 cells, 4T1 cells, RAW 264.7 cells, Balb/c nude mice, C57BL/6 mice	(220)
atherosclerosis	136	475/662		CD47	SMCs cells, RAW 264.7 cells, HUVECs cells, C57BL/6 mice	(221)
atherosclerosis	137	561/615	87 nM	LD, HClO	RAW 264.7 cells, HepG2 cells, C57BL/6J mice	(222)
atherosclerosis	138, 139		0.5 μM, 0.9 μM	GSH, H_2_O_2_	RAW 264.7 cells, mice	(223)
atherosclerosis	140	380/530, 415/645	3.8 μM	pH, phosphate	Km mice, Wistar mice, Balb/c mice	(224)
atherosclerosis	141	417/650, 550/580	0.28 μM, 0.15 mM	phosphorylation, glucose	HL-7702 cells, mice	(225)
atherosclerosis	142	640/663–738	0.437 μM	O_2_^•–^	RAW 264.7 cells, C57BL/6 mice	(226)
cardiovascular disease	143	532/565,595	3.4 × 10–8 M	NO, GSH	HUVECs cells, zebrafish	(227)
hypertension	144	405/470, 405/560	0.20 mM	H_2_O_2_	SMCC-7721 cells, HL-7702 cells, HeLa cells, Kunming mice	(228)
hyperlipidemia	145			TC, TG	RAW 264.7 cells, C57BL/6N mice	(229)
cardiotoxicity	146	570/630	34 nM	ONOO^–^	H9C2 cells, Kunming mice	(230)
myocardial fibrosis	147	405/515–565		NO	SH-SY5Y cells, RAW 264.7 cells, C57BL/6 mice	(231)

### 5.1 Fluorescent Probes for Ischemia/Reperfusion Injury

Ischemic heart disease consists of a series of cardiac issues associated with the narrowing or blockage of the cardiac arteries, leading to a lack of oxygenation (ischemia), causing acute myocardial infarction (MI, heart attack), which leads to extensive and irreversible damage to the heart.^205^ Timely reperfusion can sometimes slow disease progression by restoring coronary blood flow to ischemic tissue, although the procedure itself does carry its own in risk in the form of myocardial ischemia–reperfusion injury (MI/RI). MI/RI is a complex process involving many factors and mechanisms, including oxidative damage, mitochondrial dysfunction, apoptosis, inflammation, disruption of energy metabolism, and calcium overload.

**Figure 56:**
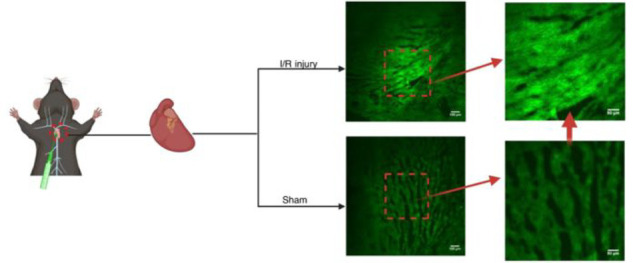
*Ex vivo* imaging of superoxide production during myocardial I/R. Reproduced with permission from ref (206). Copyright 2023 Springer Nature.

Oxidative stress is understood to be one of the main culprits of MI/RI, causing sustained damage after reperfusion. A recurring focus throughout this review, the superoxide anion is typically an excellent biomarker of oxidative stress, as most ROS ultimately originate from it. O_2_^•–^ is generated by a variety of enzymes, such as the mitochondrial electron transport chain and nicotinamide adenine dinucleotide phosphate (NADPH) oxidases. Its interactions with superoxide dismutase yields H_2_O_2_, the starting point for the production of many other ROS. In 2023, the Li group reported the design and synthesis of a series of activity-based sensing probes **126** and **127** for imaging O_2_^•–^ in living cells with high specificity (Figure 56).^206^ The authors identified 1,2,4,5-tetrazine (Tz) as an ultraspecific responsive trigger for O_2_^•–^, that was free of cross-reactivity with other ROS. Due to Tz’s intrinsic fluorescence-quenching ability, its degradation leads to fluorescence being “switched on”, with a strong enhancement of fluorescence upon exposure to O_2_^•–^. By adjusting the probe’s reactivity and emission wavelength with small structural changes, imaging of cellular O_2_^•–^ could be achieved at a variety of colors/wavelengths with outstanding spatial resolution. Given the universality of the probe design, the authors developed a high-content drug-screening model, screening 223 natural products, identifying coprostanone as capable of alleviating oxidative stress-induced damage. This study reports an interesting new superoxide-specific reactive unit and demonstrates its effective use, providing a new functional motif for incorporation into ROS selective fluorescent probes.

**Figure 57:**
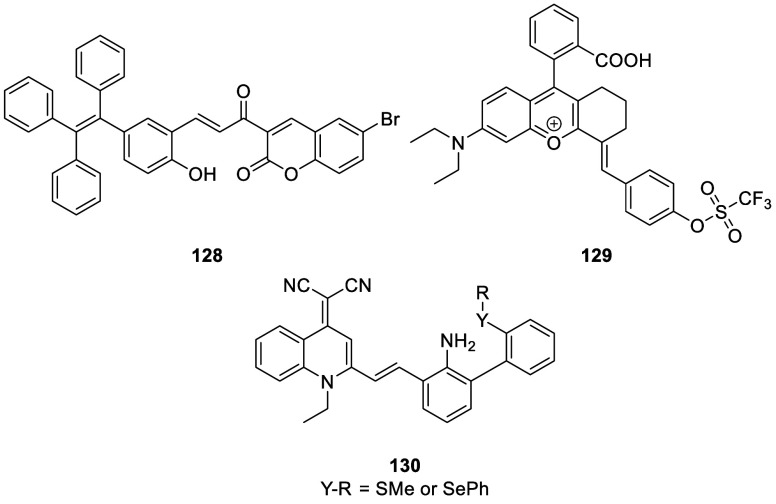
Selected fluorescent probes for MI/RI.

As the root of all ROS, O_2_^•–^ is key in the bioproduction of ONOO^–^, which is generated by the reaction of NO with O_2_^•–^. ONOO^–^ is particularly abundant in reperfusion injuries, and due to its dual ROS/RNS reactivity profile, it is often capable of more cytotoxicity than other ROS or free radicals, its overexpression ultimately leading to the loss of cardiomyocytes.^207^ To better understand this process, the Tang group created fluorescent probe **128** in 2019 for the detection ONOO^–^ in real time.^208^ Probe **128** exhibits high sensitivity and specificity for ONOO^–^ and so could be successfully used to confirm intracellular ONOO^–^ increases during ischemia. Probe **128** (Figure 57) was coadministered with an H_2_S-specific probe to image both H_2_S and ONOO^–^ concentrations simultaneously. Treatment of cells with estradiol E2, which upregulates H_2_S, showed a drastic decrease in the fluorescence of probe **128** and a strong signal from the H_2_S probe, indicating an expected H_2_S concentration increase and associated reduction in ONOO^–^. This indicates that H_2_S does, as expected, decrease ONOO^–^ concentration to reduce oxidative stress and that estradiol E2 could potentially protect heart muscle cells during ischemic events.

**Figure 58:**
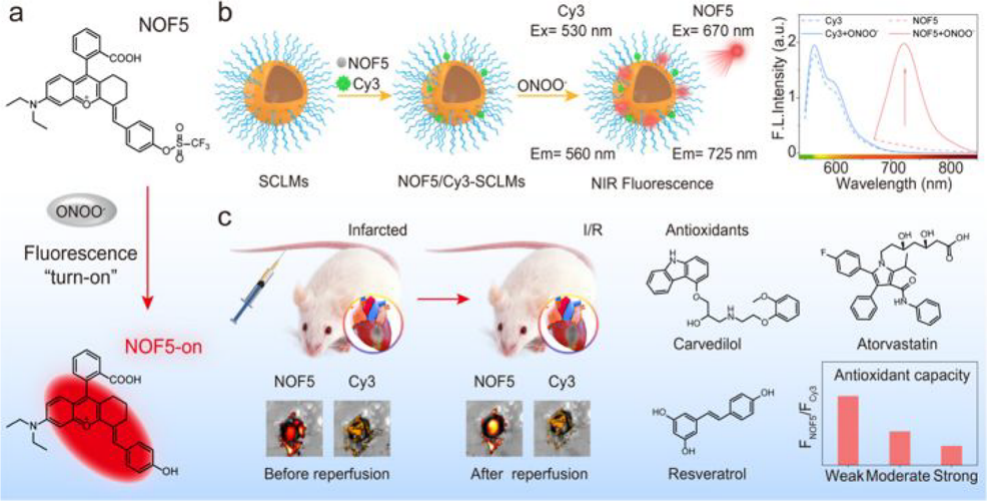
(a) Fluorescence turn-on mechanism of **129** (NOF5). (b) Construction of the ratiometric sensor using Cy3 as an internal reference. (c) The fluorescence ratio FNOF5/FCy3 in the heart could be used to monitor the level of ONOO^–^ in the heart in real time and evaluate the antioxidant capacity of drugs *in situ*. Reproduced with permission from ref (209). Copyright 2022 American Chemical Society.

In 2022, the Peng group developed the highly sensitive ratiometric fluorescent probe **129** for real-time monitoring of antioxidants in the treatment of cardiac I/R injury (Figure 58).^209^ The authors prepared this ONOO^–^-responsive NIR probe by using xanthene as the fluorophores, its ICT properties being quenched by a masking trifluoromethanesulfonate group. This group can then be removed or cleaved by ONOO^–^, thus unmasking the ICT and “turning on” the fluorescence at 725 nm. This probe was used to construct a ratiometric fluorescent nanoprobe (NOF5/Cy3SCLMs) that passively targets infarcted myocardium by coligating NOF5, with ROS-stable Cy3 as a reference fluorophore on silica cross-linked micelles. This nanoprobe was used to monitor ONOO^–^ during reperfusion, successfully evaluating the protective effects on myocardial reperfusion of three antioxidants, resveratrol, carvedilol, and atorvastatin in living cells and *in vivo*.

**Figure 59:**
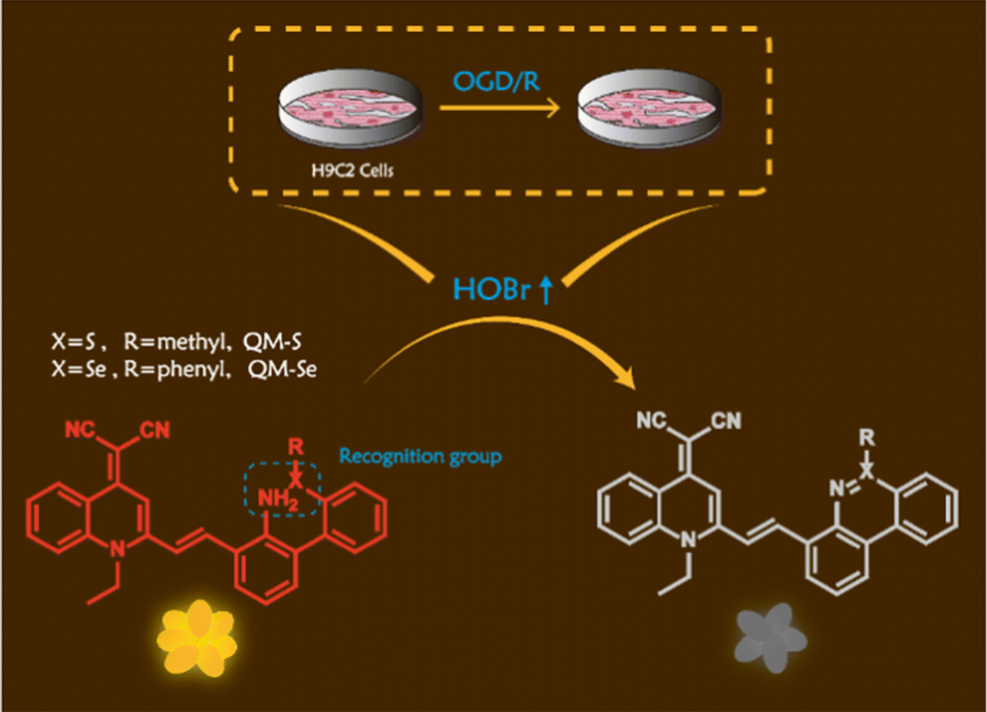
Intracellular visualization of HOBr using probe **130** during MI/RI. Reproduced with permission from ref (210). Copyright 2019 Royal Society of Chemistry.

Two fluorescent probes **130** with AIE characteristics were developed by Tang et al. in 2023 for the visualization of HOBr in MI/RI (Figure 59).^210^ Contrary to most probes in this review, these are “switch off” systems, wherein HOBr oxidizes the probe to suppress fluorescence. These probes indicated a significant increase in HOBr in H9C2 heart cells, suggesting that HOBr contributes to oxidative stress in MI/RI alongside the ROS already identified above. As MI/RI is also associated with inflammation and ferroptosis, the Tang group used antioxidant substances (NAC, E2), COX-2 inhibitors (indomethacin, sulinic acid), and a ferroptosis inhibitor (Fer-1) to reduce HOBr acid levels in H9C2 cells during OGD/R, as observed by an increase in fluorescence intensity. In this work, probe **130** was used as an imaging tool to clarify the correlation between OGD/R and HOBr in H9C2 cells.

### 5.2 Fluorescent Probes for Atherosclerosis

**Figure 60:**
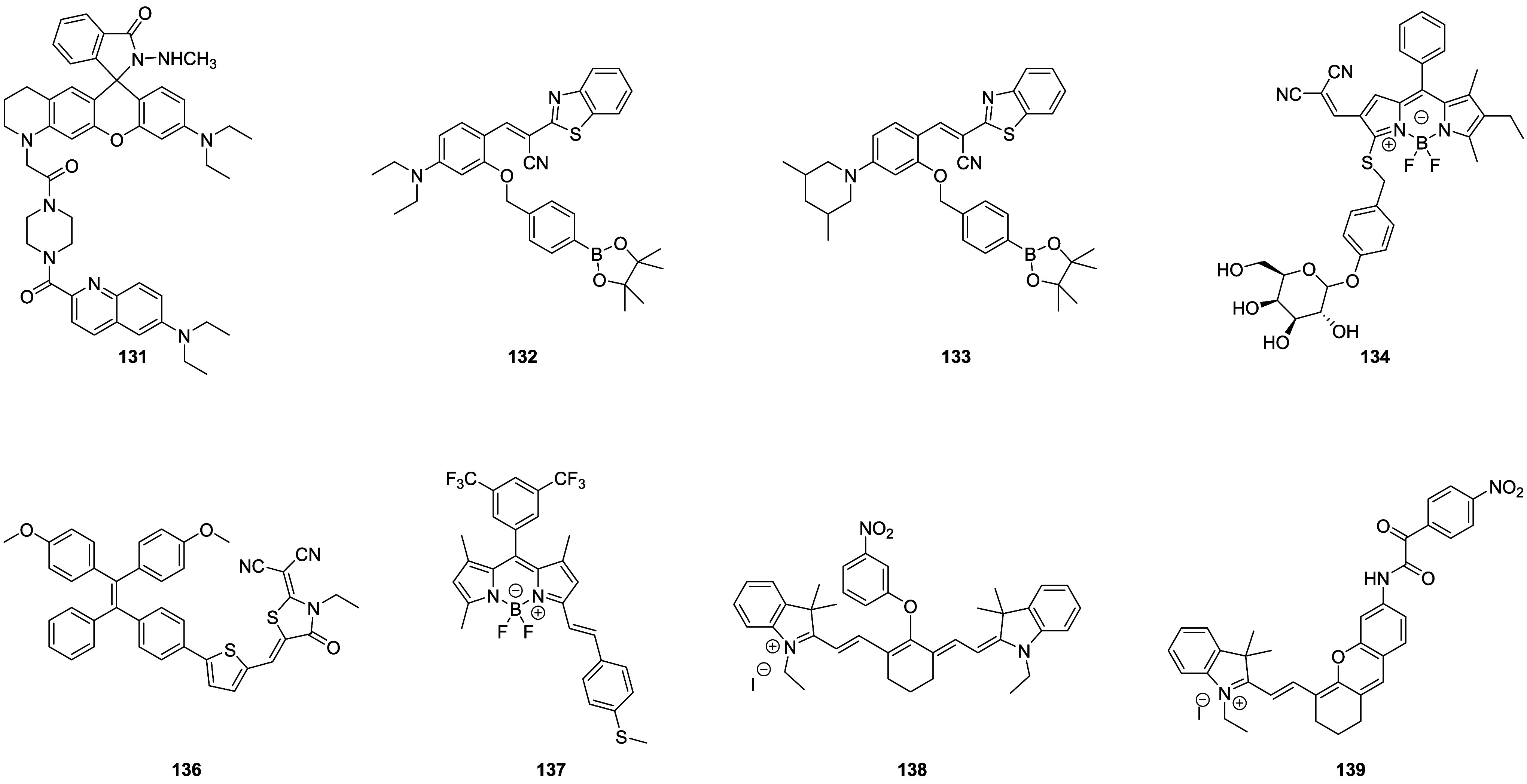
Selected fluorescent probes for atherosclerosis.

Atherosclerosis is the buildup of fats, cholesterol, and other substances in and on the artery walls. This buildup is called plaque, and over time this causes arteries to narrow, thus blocking blood flow, and can cause ruptures, damaging the vessel wall, potentially leading to blood clots.^211−213^ The formation of atherosclerotic plaque is the root cause of various cardiovascular diseases. In recent years, scientists have developed a variety of fluorescent probes based on changes in active molecules to realize the detection and intervention of atherosclerosis (Figure 60).

**Figure 61:**
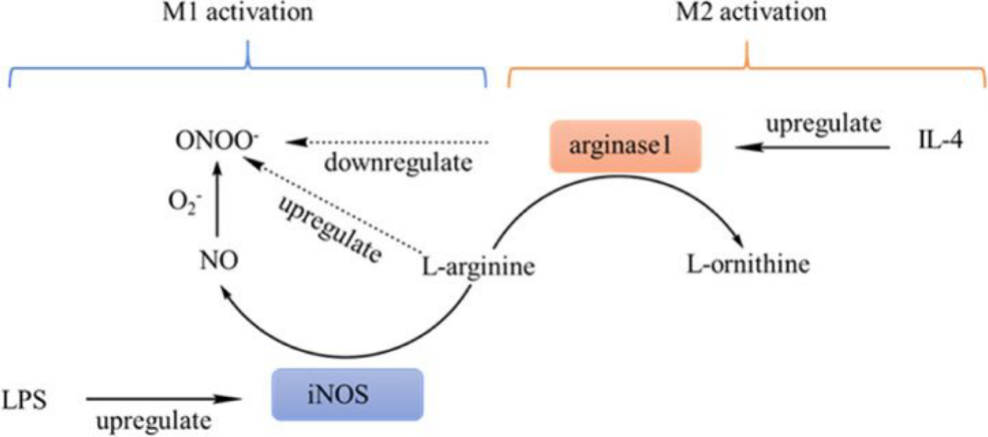
Arginase 1 downregulates ONOO^–^ Reproduced with permission from ref (214). Copyright 2021 American Chemical Society.

In 2021, the Canary group described the development and biological application of fluorescent probe **131** to measure ONOO^–^ levels *in vitro* and *in vivo* (Figure 61).^214^ TP probe **131** is a reaction-based ratiometric probe with a 100 nm red-shift in the emission spectrum, capable of sensing subtle fluctuations in ONOO^–^ concentration. In cell and mouse experiments, it was found that ONOO^–^ in macrophages was inversely correlated with arginase-1 activity. Furthermore, in atherosclerotic mice probe **131** enabled the observation of ONOO^–^ fluctuations in both progressing and regressing plaque, demonstrating increased ROS in atherosclerotic mice. These results support the conjecture that there is enrichment in M2-like macrophages with high expression of arginase-1 during regression, reducing ONOO^–^ levels in regression stages of atherosclerosis. Given the deleterious effects of ONOO^–^ in atherosclerosis, the potential antiatherosclerosis effects of arginase-1, elucidated using probe **131**, could be of vital importance in developing atherosclerosis therapies in the future.

**Figure 62:**
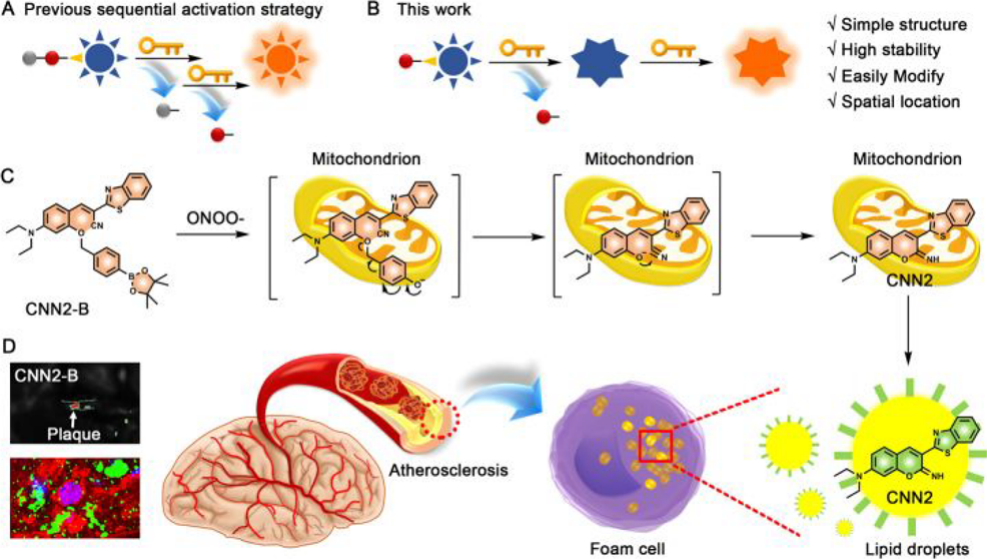
Design of the ONOO^–^/LD sequence-activated fluorescence probe **132**. (A,B) Design strategies employed in previous work (A) and for probe **132** (B). (C) Mechanism of probe **132**. (D) Intraoperative Imaging of probe **132**. Reproduced with permission from ref (215). Copyright 2023 American Chemical Society.

In 2023, Zheng et al. reported a dual-analyte sequentially activated logic-based fluorescence reporter system **132**, which targets ONOO^–^ and lipid droplets to accurately identify atherosclerotic plaques *in vivo* (Figure 62).^215^ This probe was designed as a double-locked fluorescence system, which reacts first with ONOO^–^ to remove the *p*-benzylBPin ROS-recognition motif, liberating a phenolic functionality capable of ring closing with the adjacent acrylonitrile to produce a fluorescent coumarin derivative. The fluorescence of probe **132** is strongly solvent-dependent, and so can differentiate between aqueous and lipid droplets, emitting only in the latter environment. It therefore does not need a secondary targeting or reactive unit, as it is inherently selective for lipid (highly nonpolar) environments. This also means that the fluorescence enhancement on entering lipid droplets is extremely high at 365-fold, thus exhibiting both better selectivity and a better signal-to-noise ratio than typical commercial probes.

**Figure 63:**
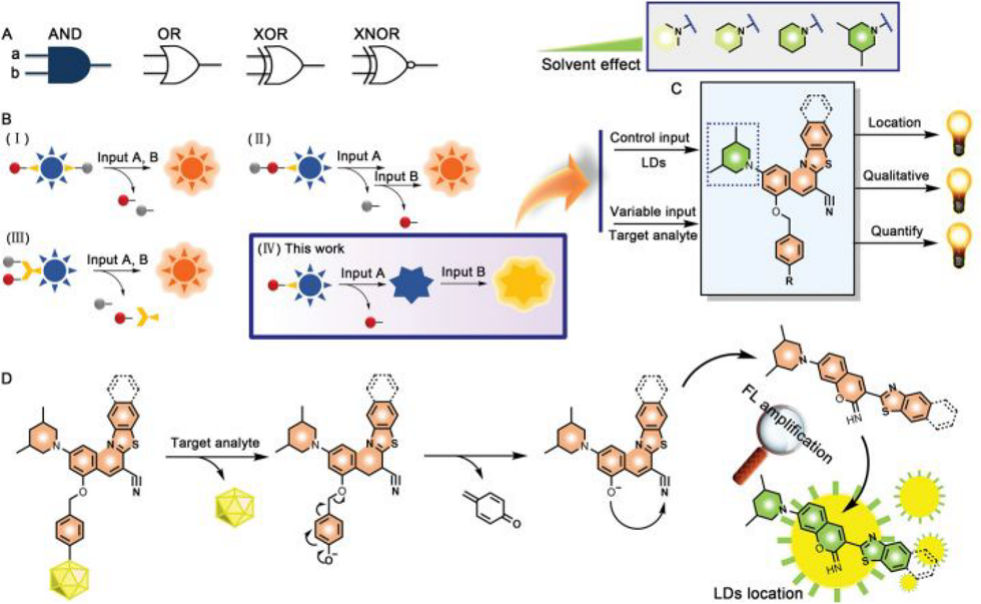
“AND” molecular logic gate probe **133**. (A) Molecular logic gates. (B,C) “AND” molecular logic gate design principles. (D) Fluorescence activation mechanism of probe **133**. Reproduced with permission from ref (217). Copyright 2023 John Wiley & Sons.

This type of “AND” molecular logic gate is becoming increasingly popular, providing researchers with a highly effective way of maximizing signal-to-noise ratios by increasing fluorescence enhancement (*F*/*F*_0_).^216^ In the example of probe **132** above, lipid droplets were utilized as a controllable background input, with the target analyte set as a variable input. Due to this double locking, the fluorescence is fully quenched, thereby obtaining an extreme *F*/*F*_0_ ratio for the target analyte. Using this approach again, the same group redesigned their probe to produce **133** (Figure 63).^217^ Following the same reaction, detection, and fluorescence manifolds, this improved version was capable of achieving an astounding 2600-fold fluorescence enhancement on activation by ONOO^–^ followed by entrance into the droplets. This framework should provide a highly effective and innovative platform for the development of novel high signal-to-noise ratios and impressive fluorescence enhancements.

**Figure 64:**
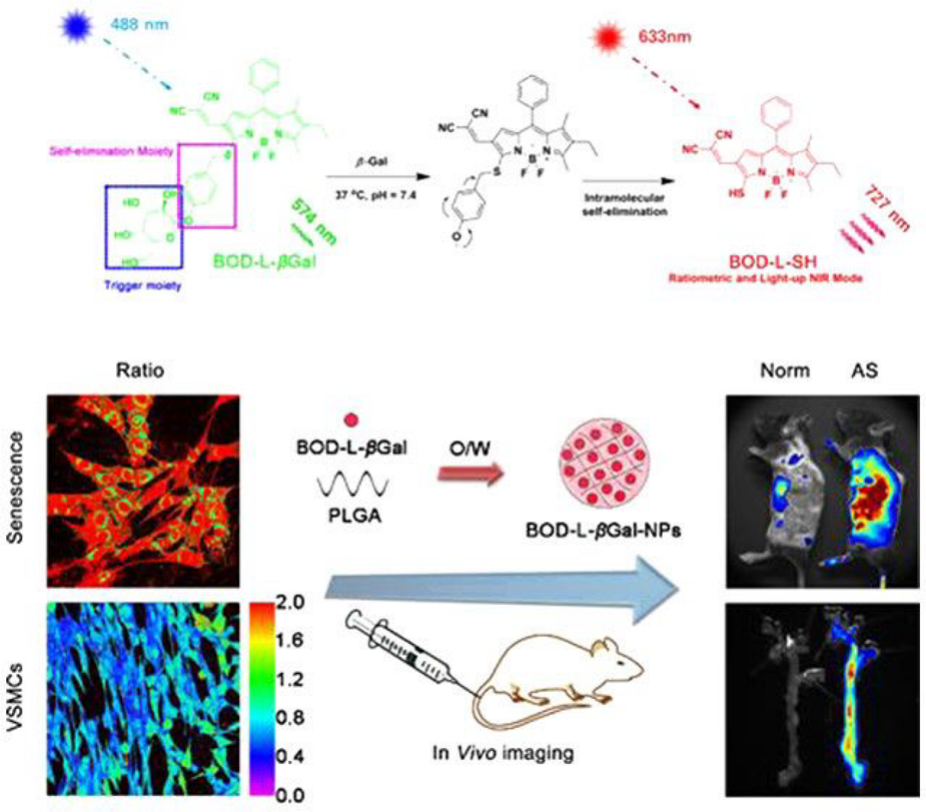
β-Gal sensing mechanism of probe **134**. Reproduced with permission from ref (218). Copyright 2020 American Chemical Society.

Recent developments have indicated that senescent cells (vascular endothelial, smooth muscle, and macrophages) play a role in the formation and development of atherosclerosis. One method of imagining senescence is by imaging senescence-associated β-galactosidase (SA-β-Gal), as demonstrated by the groups of Hu and Gu with NIR probe **134** in 2020. **134** was encased in a PLGA core to prepare SA-β-Gal-sensing nanoparticles (Figure 64).^218^ These nanoprobes were shown to accumulate effectively in arteries, enabling the imaging of senescent cells in atherosclerotic mice.

**Figure 65:**
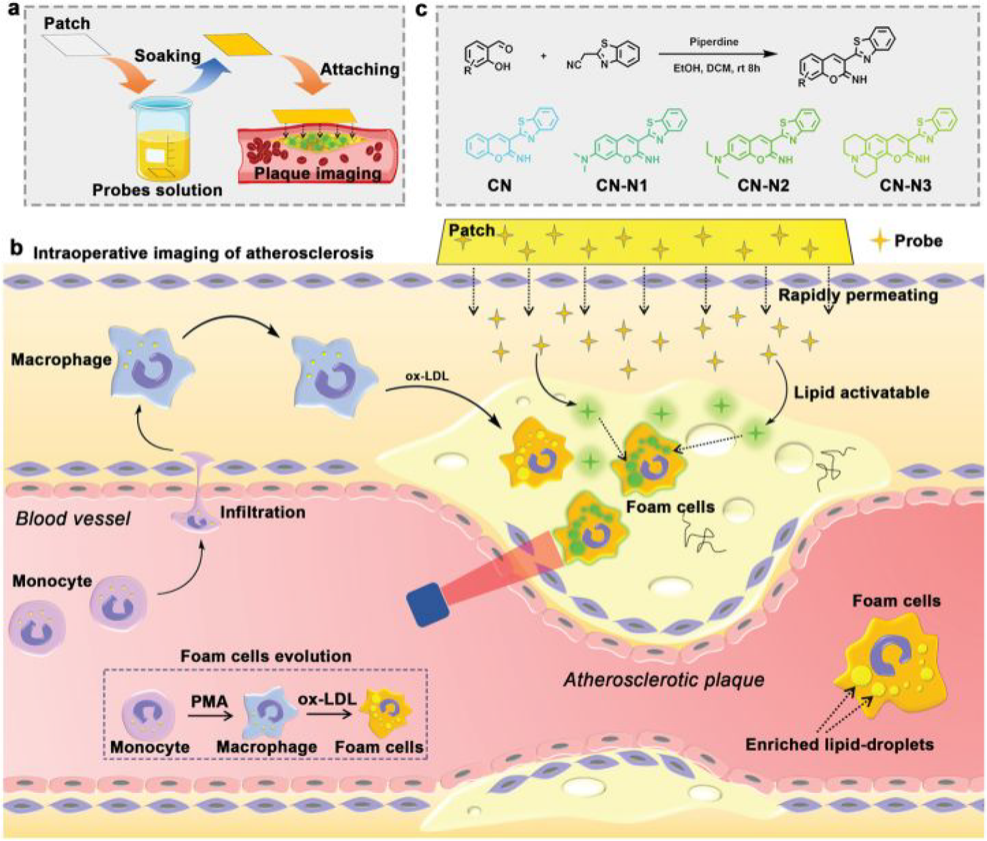
Lipid-activatable fluorescent probe **135** for intraoperative imaging of atherosclerotic plaques using *in situ* patches. Reproduced with permission from ref (220). Copyright 2022 John Wiley & Sons.

As for many progressive diseases, surgical removal of lesions and plaque can be an effective and sometimes the only treatment for atherosclerosis. In this situation, the ability to visualize atherosclerotic plaque during surgery is key to achieving positive outcomes.^219^ As we have discussed for some of the previous examples, fluorescence imaging offers a promising way of achieving this, and new methods for fluorescence-guided surgery of atherosclerosis are being developed. For example, lipid-activated patches soaked with fluorescent probes of a similar type to probes **135** above can be attached to the outside of arteries, diffusing the probe into the tissue to enable quick and precise location of atherosclerotic plaque during surgery in atherosclerotic mice (Figure 65).^220^ By quickly identifying abnormal accumulation of lipid droplets in foam cells, imaging of plaque can be performed within 5 min. A plaque-to-normal fluorescence ratio of 4.3 was achieved, enabling facile identification of plaque and nonplaque tissue for good delineation of carotid atherosclerosis. Visible fluorescence bioimaging using lipid-activated probes was found to accurately identify plaque as small as 0.5 mm in diameter. The development of intraoperative fluorescence imaging of plaque *in situ* during surgery shows good promise for future clinical evaluation, and for the use of fluorescent-probe-soaked patches in image-guided surgical intervention.

In 2022, the Ding group reported an AIE-based nanoprobe **136**, based on a rhodanine derivative for precise, sensitive, and rapid early detection of atherosclerotic plaque and drug screening.^221^ Probe **136** exhibits a high molar extinction coefficient, large photoluminescence quantum yield, and very good absorption/emission spectral red-shift relative to typical reference probes. The nanoprobes were synthesized using amphiphilic copolymers as a matrix for probe encapsulation and were further surface-functionalized with anti-CD47 antibodies to specifically bind to overexpressed CD47 in atherosclerotic plaques. These nanoprobes can effectively identify said plaques at different stages in apolipoprotein E-deficient mice (atherosclerosis model). Of particular note was the ability of this probe to identify atherosclerotic plaques in early stage atherosclerosis, well before CT or MRI imaging was even possible. Probe **136** was therefore used to assess the antiplaque potential of atorvastatin and GW3965, demonstrating the ability of both drugs to reduce atherosclerotic plaques, in line with their known clinical performance.

**Figure 66:**
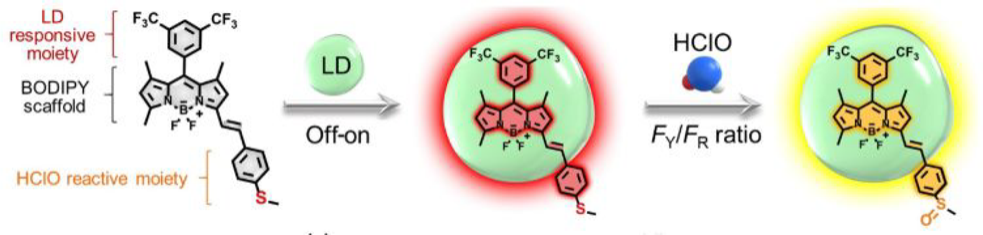
Schematic illustration of iSHERLOCK probe **137** for “off–on” and ratiometric detection of LDs and HClO. FY and FR represent the fluorescent intensities (FI) in the yellow and red channels, respectively. Reproduced with permission from ref (222). Copyright 2022 John Wiley & Sons.

In 2022, the Liu group reported probe **137**, a dual-targeting sequential fluorescence system to precisely identify atherosclerotic plaque *in vivo* and *in vitro*, which they called in-sequence high-specificity dual-reporter unlocking (iSHERLOCK) (Figure 66).^222^ iSHERLOCK detects both HClO and lipid droplets, two hallmarks of atherosclerosis. In a manner similar to previous probes featured in this review, this probe is nonfluorescent in aqueous media, “switching on” on entering nonpolar lipid droplets, with HClO then triggering oxidation to shift the fluorescence signal to create the dual output.

**Figure 67:**
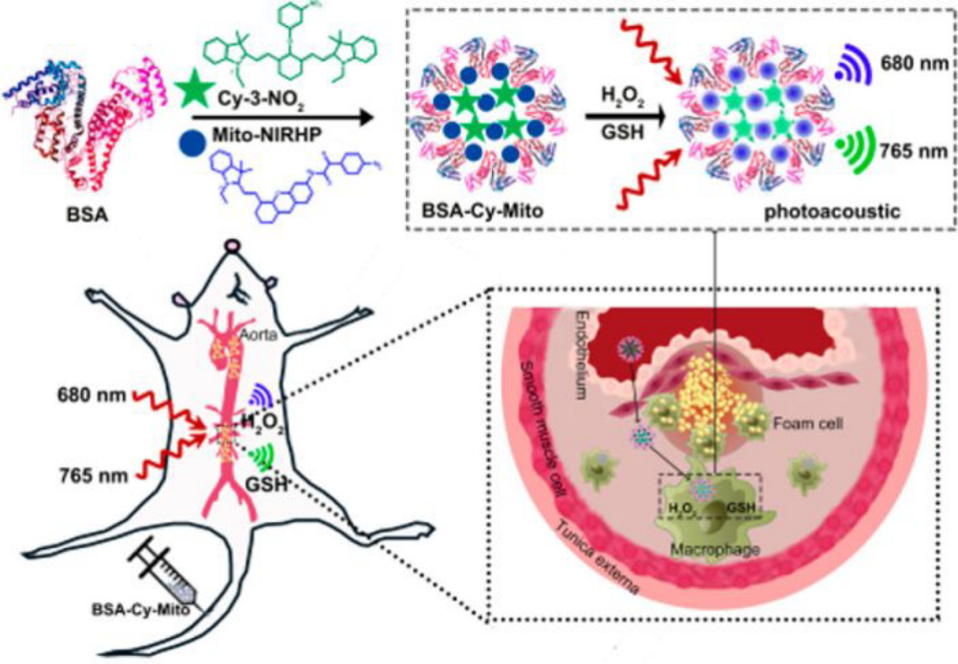
Structures of GSH/H_2_O_2_-responsive BSA-Cy-Mito nanoprobes based on fluorescent probes **138** and **139** for in vivo PA imaging of redox state to assess atherosclerotic plaque vulnerability. Reproduced with permission from ref (223). Copyright 2019 American Chemical Society.

Two NIR fluorescent probes **138** and **139** were reported by the Tang group in 2019 for the photoacoustic imaging of atherosclerotic plaque vulnerability, looking specifically at plaque oxidative stress/inflammatory activity. These probes were used to examine the oxidative stress-linked GSH/H_2_O_2_ redox couple and were combined with bovine serum albumin (BSA) to form BSA-Cy-Mito nanoprobes (Figure 67).^223^ This type of BSA-based self-assembly has good biocompatibility and an extended blood circulation time, highly enhanced permeability and retention, and generates good and strong GSH- and H_2_O_2_-specific outputs at 765 and 680 nm. The BSA-Cy-Mito nanoprobes were used for GSH/H_2_O_2_ detection in oxidized low-density lipoprotein-activated macrophages and in high-fat diet-fed apolipoprotein e-deficient mice, with accurate diagnosis of redox-related inflammatory processes. Systemic administration of BSA-Cy-Mito allowed for the differentiation between vulnerable plaques and stable plaques according to their differing redox states. This sensitive redox-responsive PA nanoprobe may be a powerful tool for early identification of vulnerable plaques to facilitate the implementation of successful preventative treatment strategies.

**Figure 68:**
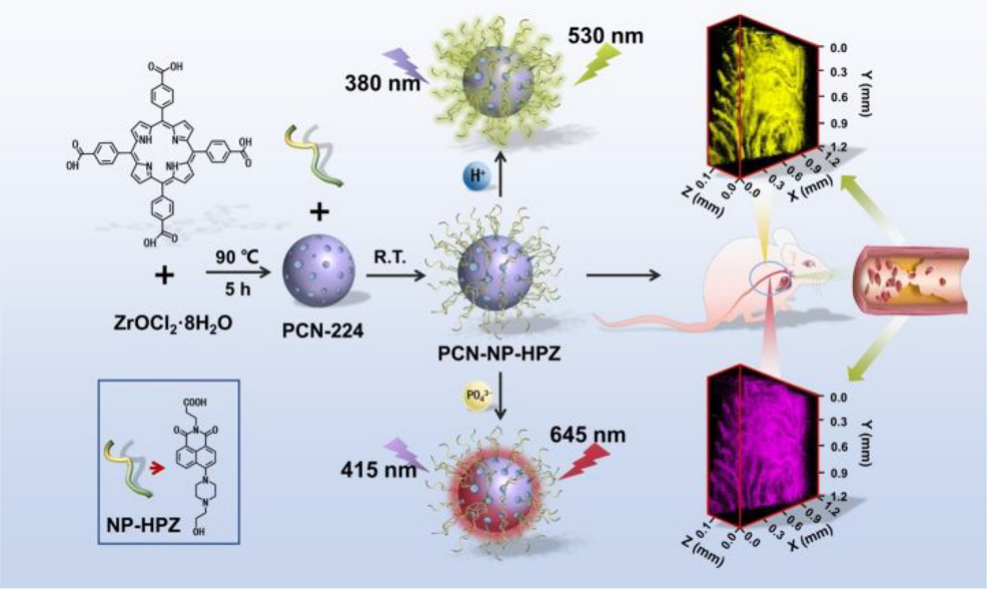
Synthesis of probe **140** and its application in fluorescence detection and two-photon fluorescence imaging of atherosclerotic mice. Reproduced with permission from ref (224). Copyright 2023 John Wiley & Sons.

Inflammatory and distressed environments typically also involve variations in pH, and atherosclerosis is no exception. This was imaged by Tang and co-workers in 2023, using a MOF-based dual-detection fluorescent nanosensor PCN-NP-HPZ (Figure 68, probe **140**).^224^ The simultaneous detection and imaging of pH and phosphorylation was achieved by pH-sensitive groups piperazine and phosphate-binding Zr^IV^. Using probe **140**, Tang et al. were able to monitor changes in blood pH and phosphorylation at various stages of plaque formation, showing increased acidity in the inner wall of the aorta of atherosclerotic mice, which they linked to vascular endothelial inflammation. Concurrently, phosphorylation levels were higher than in normal mice, providing valuable insights into the mechanism of plaque formation and atherosclerotic environments in early stages of atherosclerosis.

**Figure 69:**
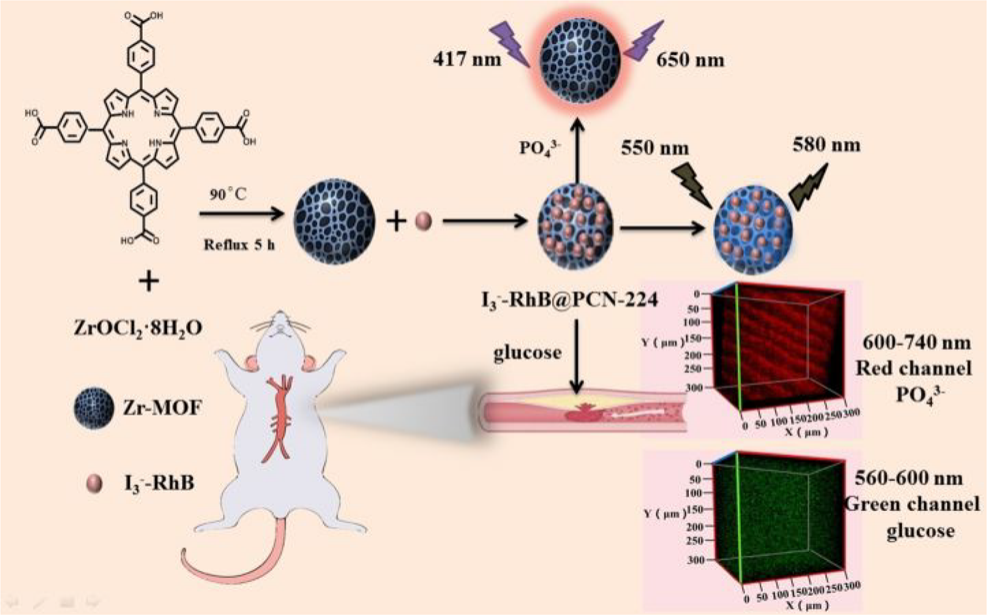
Synthesis of nanoprobe **141** and its application for detection and imaging of phosphorylation and glucose levels in early atherosclerosis models. Reproduced with permission from ref (225). Copyright 2023 John Wiley & Sons.

In 2023, the Tang group also developed another MOF-based sensor for atherosclerosis, this time aimed at monitoring the condition prior to plaque formation. This was achieved with probe I_3_^–^-RhB@PCN-224 (probe **141**) for monitoring phosphorylation and glucose in this instance (Figure 69).^225^ Probe **141** was synthesized by postmodification of the MOF with iodine (I_3_^–^)-rhodamine B. Phosphorylation could again be monitored through interaction of the Zr^IV^ and phosphate, with the I-RhB component recognizing glucose. Using probe **141**, the authors studied atherosclerosis during the early nonplaque phase to identify levels of both targeted analytes. TP imaging results showed that early atherosclerotic mice had higher protein phosphorylation and glucose levels than normal mice, which may provide key insights for future treatment and study.

**Figure 70:**
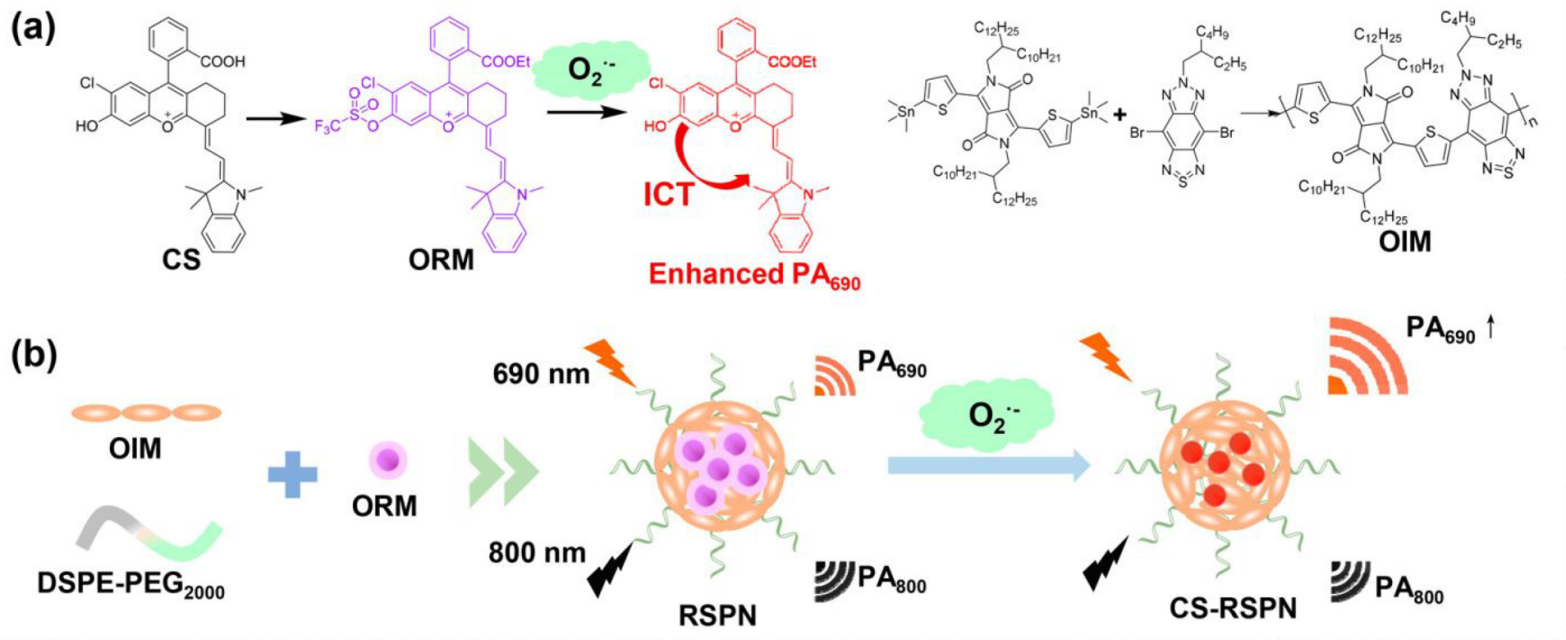
(a) Structure and turn-on mechanism of the fluoroescent probe **142**. (b) One-step self-assembly of RSPNs. Reproduced with permission from ref (226). Copyright 2021 American Chemical Society.

In 2021, the Zhang group developed a novel ratiometric semiconducting polymer nanoparticle (RSPN, probe **142**) for photoacoustic imaging of vulnerable plaques in apolipoprotein e-deficient mice with pneumonia, which is known to greatly increase the risk of plaque rupture (Figure 70).^226^ Probe **142** reacts with O_2_^•–^, exhibiting an enhanced photoacoustic signal around 690 nm, while the 800 nm emission serves as an internal invariable photoacoustic reference. Probe **142** could reliably measure O_2_^•–^ within aortic atherosclerosis in a ratiometric manner, enabling researchers to assess the degree of oxidative stress in vulnerable plaque. Notably, probe **142** was able to distinguish between plaque-bearing mice, plaque-bearing mice with pneumonia, and healthy mice, thus proving a useful tool for predicting plaque vulnerability.

### 5.3 Fluorescent Probes for Other Cardiovascular Diseases

Endothelial cells are effectively the gatekeepers of cardiovascular homeostasis, forming a barrier that selectively restricts or enables the movement of macromolecules between the blood and the vessel wall. There is strong evidence that oxidative stress can cause endothelial dysfunction and thus the development of cardiovascular disease. Clarification of signaling pathways associated with NO (vasodilator) and GSH (NO-reducing) is therefore key to preventing CVD in general and to gaining a deeper understanding of the downstream effects of ROS-mediated endothelial damage.

**Figure 71:**
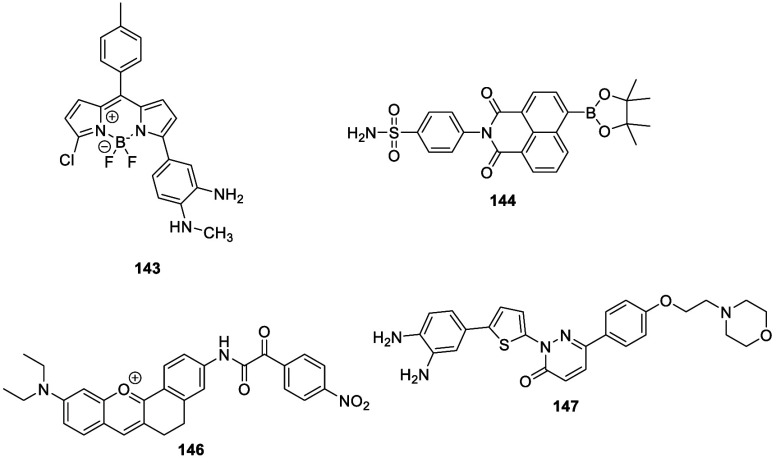
Selected probes for cardiovascular disease.

**Figure 72:**
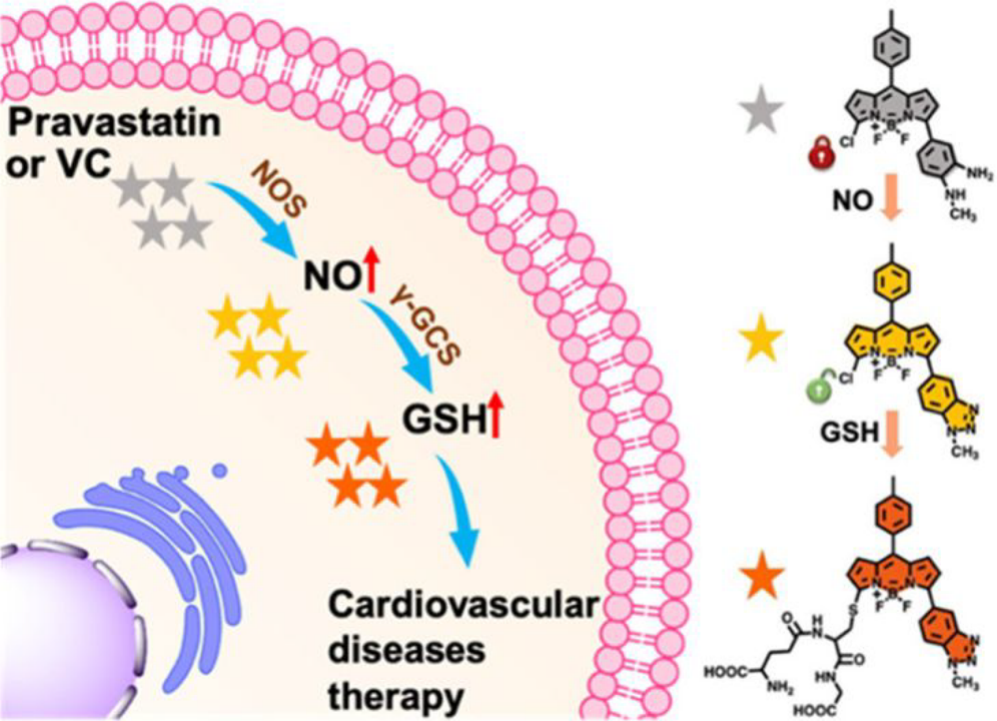
Cardiovascular disease therapy accompanied by sequential generation of NO and GSH monitored by probe **143** in human umbilical vein endothelial cells. Reproduced with permission from ref (227). Copyright 2021 American Chemical Society.

To this effect, in 2021, Yang et al. developed the BODIPY-based fluorescent probe **143** for the identification of NO and GSH (Figure 71, Figure 72).^227^ The probe exhibited a fluorescence turn-on response with NO, followed by red-shifted emission in the presence of GSH. This mechanism of sequential activation allowed NO-induced GSH upregulation to be visualized for the first time in drug-treated endothelial cells and zebrafish, revealing a new NO/γ-glutamylcysteine synthetase/GSH signaling pathway which could have significant implications for the development of cardiovascular therapies.

Golgi apparatus-associated oxidative stress is closely linked to the occurrence and development of hypertension, in particular H_2_O_2_, which is directly correlated to the degree of Golgi oxidative stress. In 2019, the Tang group developed a novel Golgi apparatus targeting probe **144** for *in situ* imaging of H_2_O_2_*in vivo*.^228^ This rather simple probe is composed of three parts: a naphthalimide fluorophore, a BPin peroxide-reactive functional group, and a Golgi-targeting benzenesulfonamide moiety. Synthesis and further modification of this probe are exceedingly simple, and so this basic structural scaffold should be of great use to the sensing community. Using probe **144**, the authors explored the production of H_2_O_2_ in Golgi oxidative stress and found elevated Golgi H_2_O_2_ levels in the kidneys of hypertensive mice.

**Figure 73:**
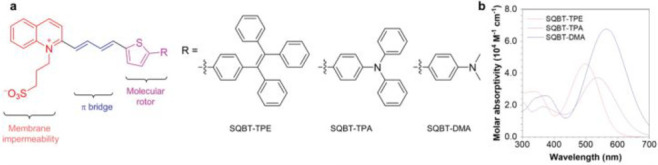
(a) Molecular design and preparation of probes **145**. (b) Absorption spectra of probes **145** in DMSO. Reproduced with permission from ref (229). Copyright 2022 John Wiley & Sons.

Further adding to an already significant body of work in the field, in 2022 the Tang group reported a series of intelligent NIR fluorophores for the diagnosis of hyperlipidemia (Figure 73, probes **145**).^229^ As they are based on a molecular rotor donor–zwitterionic unit acceptor template, they exhibit twisted ICT properties, and so have minimal fluorescence in aqueous environments, but emit strongly on aggregation in highly viscous media. As these species reversibly switch aggregation state and fluorescence state simply based on environment, requiring no chemical reaction or structural change to occur, they are termed “smart aggregates”. Interestingly, these luminescent substances exhibited both NIR-II and NIR-III luminescence with a large Stokes shift (950 nm), suggestive of their great potential for the development of “ultra-tissue-transparent” imaging agents. Both *in vitro* detection and *in vivo* imaging of hypolipidemia (HLP) was achieved in mouse models, with a good linear relationship between emission intensity and multiple pathological parameters in blood samples from HLP patients.

**Figure 74:**
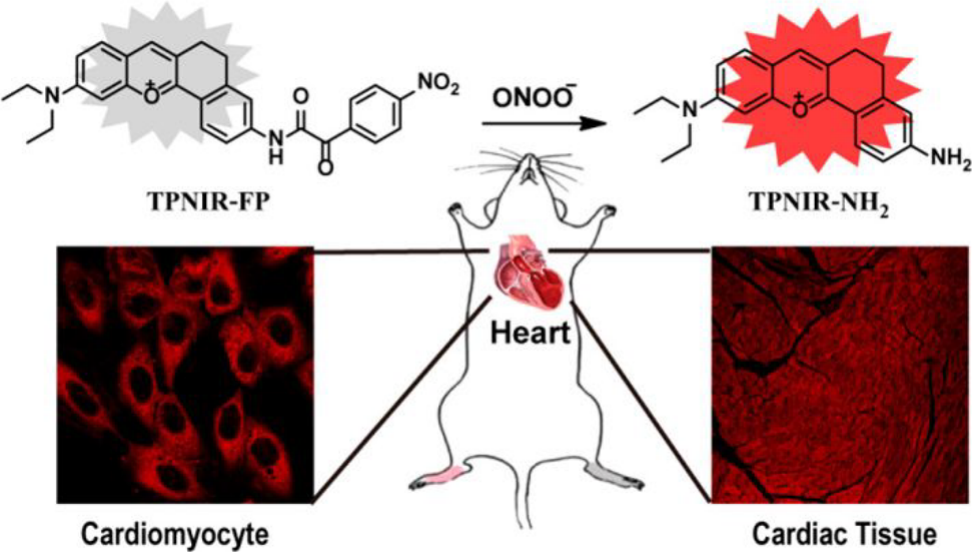
Schematic illustration of probe **146** for ONOO^–^ imaging. Reproduced with permission from ref (230). Copyright 2018 American Chemical Society.

Cardiotoxicity can be a significant issue in the development and adoption of new drugs, an example of which is the anthracycline class of anticancer drugs which boast high efficacy but also significant cardiotoxicity, posing major challenges for clinical use. In 2018, to better assess cardiotoxicity risk, the Tang group developed a TP NIR fluorescent probe, probe **146**, for imaging ONOO^–^ overexpression in mitochondria (Figure 74).^230^ Using this probe, they demonstrated that mitochondrial ONOO^–^ is upregulated in the early stages of anthracycline cardiotoxicity in cardiomyocytes and mouse models. It can therefore be used as an early biomarker of drug-induced cardiotoxicity, allowing for better drug screening in development, and the prevention of negative cardiac outcomes during treatment.

**Figure 75:**
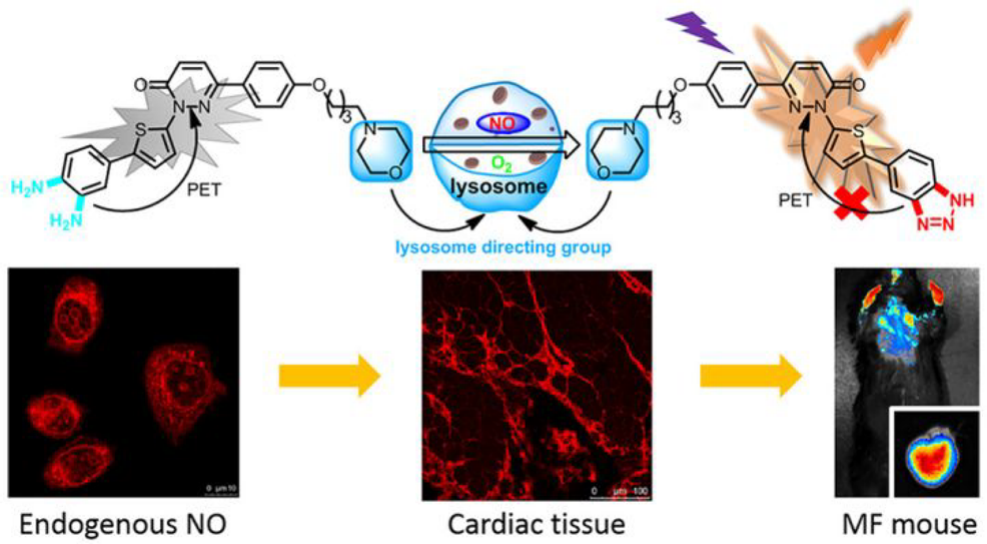
Schematic Illustration of **147** for NO Imaging. Reproduced with permission from ref (231). Copyright 2020 American Chemical Society.

Probe **147**, developed by the Xu group in 2020 for the imaging of myocardial fibrosis (Figure 75).^231^ It was designed to show a rapid response on reaction with endogenous and exogenous NO to cleave its triazine motif, suppressing PeT quenching. This probe was successfully used to track and study the production of NO in animal tissues, specifically lysosomal nitric oxide due to its morpholine lysosome-targeting group. In mouse myocardial fibrosis models, probe **147** exhibited good imaging properties *in vivo*, elucidating a progressive relationship between myocardial NO production and myocardial fibrogenesis.

## 6 Fluorescent Probes for Inflammation

**Figure 76:**
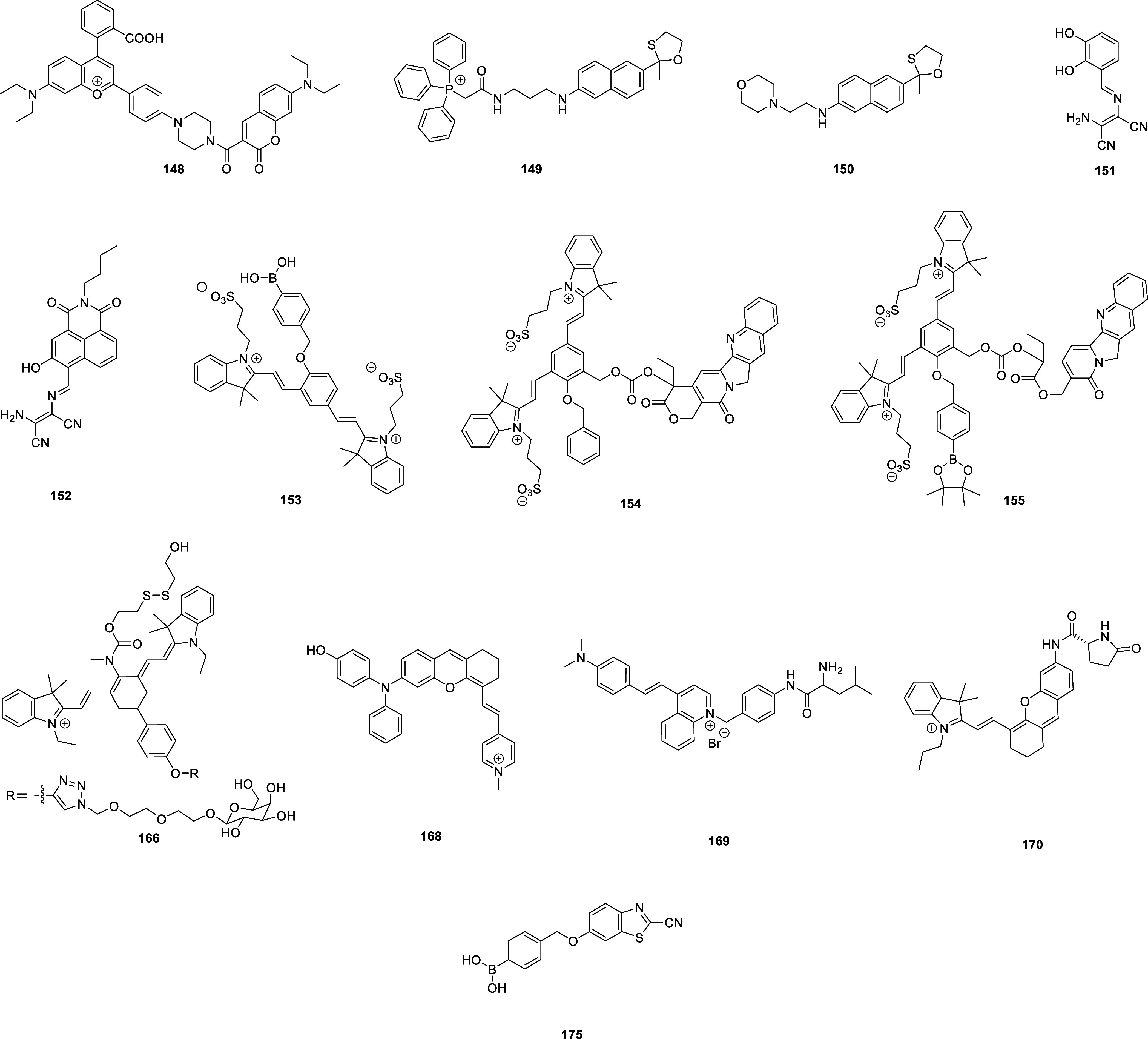
Selected fluorescent probes for inflammatory disease and RA of mice. Probe **152** is particularly effective for evaluating the early therapeutic effects of antiarthritic drugs on HOCl levels in RA mouse models.

Inflammation (inflammatory response) is a basic pathological process typically triggered as part of the defense mechanism of tissue when it is injured, for instance, by trauma or infection. Inflammation manifests itself typically through redness, heat, pain, loss of function, and swelling, as well as systemically through fever and changes in peripheral white blood cell count. Although inflammation is a basic pathophysiological response to damage, its effects are double-sided. Short-term inflammation helps to clear inflammation-causing substances and ultimately promotes recovery, whereas long-term inflammation increases the body’s energy consumption and leads to tissue and organ damage, examples of which have been seen in the previous section. Normal immunity is key to the healthy function of the body; excessive inflammation can aggravate conditions and lead to longer-term damage. Conversely, too weak an inflammatory response can lead to chronic disease arising from ineffective immune response. This section will look more closely at inflammation and discuss a range of fluorescent probes that have been developed for the diagnosis and treatment of various inflammatory conditions (Table 5, Figure 76).

**Table 5:** Selected Fluorescent Probes for Inflammation

inflammation	probe	λ_ex_/λ_em_ (nm)	LOD	bioactive molecule	biological model	ref
hepatitis	**148**	405/630–675	11.30 nM	ONOO^–^	inflamed mouse model	(232)
LPS/MPA induced inflammation	**149**, **150**	375/500–634, 375/500–610	16.6 nM	HClO	inflamed mouse model	(233)
rheumatoid arthritis	**151**, **152**	370/427, 490/575	208.9 × 10^–9^ M 17.3 × 10^–9^ M	HClO	LPS induced zebrafish and rheumatoid arthritis mice	(234)
LPS induced inflammation	**153**	570/715		H_2_O_2_	LPS-induced acute inflammation model in mice	(235)
tumor-related inflammation	**154**, **155**	595/700		H_2_O_2_	mice with U-87 MG tumors	(236)
acute and chronic inflammation	**156**	405/	100 × 10^–6^ M	ONOO^–^	bacterial infected inflammatory skin in mice	(237)
hepatitis	**157**	780/845	0.55 μM	·OH, H_2_S	LPS-induced liver inflammation in mice	(238)
interstitial cystitis	**158**	808/950	0.74 μM	H_2_O_2_	interstitial cystitis mice	(239)
acute liver inflammation	**159**	640/730	0.27 μM	ROS	LPS/D-GalN-induced acute liver inflammation in mouse model	(240)
inflammatory bowel disease	**160**			H_2_O_2_	LPS-activated RAW264.7 cells	(241)
inflammatory bowel disease	**161**	460/709	0.856, 0.26 μM	NO	LPS-induced inflammation mice, inflammatory bowel disease mice	(242)
acute and chronic inflammation	**162**	410/600		ROS	arthritis mice	(243)
immune-mediated inflammatory diseases	**163**	675/730	0.34 μM	ROS	the autoimmune hepatitis and hind paw edema mouse models	(244)
lymph nodes and arthritis	**164**	808/	17.4 μM	H_2_O_2_	lymph nodes and arthritis mice	(245)
tumor-related inflammation	**165**	745/800		pH, H_2_O_2_	inflamed tumor mice	(246)
acute hepatitis	**166**	610/750; 720/800	0.09 μM	Sec	acute hepatitis mice	(247)
liver inflammation	**167**	808/1200		temperature	LPS-induced hepatitis mice	(248)
fatty liver, inflammation and cancer	**168**	560/650–754		ROS	fatty liver and tumor mouse model	(250)
inflammation and cancer	**169**	550/700	0.08691 U/L	LAP	zebrafish inflammation model	(251)
hepatitis	**170**	670/700	0.18 ng/mL	PGP-1	LPS/D-Gal induced mice	(252)
LPS induced inflammation	**171**	540/670		MPO	LPS induced inflammation mice and 4T1-luc tumor-bearing mice	(253)
carrageenan-induced inflammation	**172**	581/605		COX-2, ROS	inflammation and tumor mouse model	(254)
acute and chronic inflammatory diseases	**173**	675/695		caspase-1	endotoxin shock, inflammatory bowel disorder, transplanted cancer, and Alzheimer’s disease mice	(255)
allergic airway inflammation	**174**	600/700–900		ovalbumin	asthma mouse model	(256)
acute inflammation	**175**		0.5 μM	H_2_O_2_, caspase-8	LPS-induced acute inflammation in mouse model	(257)
encephalitis	**176**	808/1030		N/A	brain inflammation mouse model	(258)

One of the key elements of inflammation is oxidative stress and abnormal production and high concentrations of ROS. In 2017, Yuan and co-workers developed probe **148** a novel TP ratiometric fluorescence probe based on FRET, designed using a combined rational design and dye-screening approach.^232^ This innovative approach led them to select a ROS-stable coumarin fluorophore as the FRET donor and a rhodamine FRET acceptor, which also acts as the ONOO^–^-specific cleavable site, destroying the fluorophore in the process, suppressing FRET, and triggering a fluorescence response. Probe **148** has a fast reaction speed (<20 s), high sensitivity (LOD = 11.30 nM), and good selectivity for ONOO^–^. The authors successfully employed this probe to assess the amount of ONOO^–^ in HepG2/RAW264.7 cells, as well as image oxidative stress in inflammatory mouse models.

As a key part of oxidative stress, HOCl is also of interest in inflammation, with its strong oxidizing power harnessed by the innate immune system to fend off pathogens and regulate programmed cell death processes such as apoptosis and pyroptosis. The exact mechanisms of HOCl distribution and production, however, remain unclear, in large part due to difficulties in detecting HOCl arising from its very low concentration and short lifetime. In 2015, Chang et al. reported the first TP fluorescent HOCl probe and its mitochondrial and lysosome-targeting derivatives, probes **149** and **150**.^233^ These probes respond rapidly to HOCl exposure (seconds) and exhibit good selectivity, and high sensitivity (LOD = 20 nM). Each probe was shown to successfully detect HOCl in their given organelle in both live cell experiments and *in vivo* in mouse models, with high HOCl content visualized in macrophages in an inflammatory state.

HOCl sensing is also a key for the diagnosis and early evaluation of therapies for rheumatoid arthritis (RA). The need for accurate bioassays has been in part the focus of Zhang, Meng, and co-workers, who in 2018 developed two fluorescent probes **151** and **152** for the quantitative monitoring and visualization of inflammatory response-related HOCl levels *in vitro* and *in vivo*.^234^ In the presence of HOCl, both probes exhibited fluorescence “off–on” response due to the specific HOCl-triggered imine C=N oxidative cleavage. Both probes showed fast response, high sensitivity, and good selectivity, making them suitable for the quantification of HOCl under simulated RA physiological conditions. Using probe **152**, fluorescence imaging and flow cytometry were used to analyze the level of HOCl in the lysosomes of inflammatory mimic cells and to visualize the HOCl production in endotoxin-induced inflammation of adult zebrafish.

As one of the most abundant reactive oxygen species, H_2_O_2_ plays an important role in the occurrence and development of inflammation. In 2011, the Shabat group designed a new NIR cyanine fluorochrome (probe **153**) by modulating the π-electron system of cyanine molecules through small structural changes, introducing a “turn on” mechanism to enable H_2_O_2_ quantification in LPS-induced inflammation mouse models.^235^ Probe **153** was synthesized via a two-step process and exhibited excellent optical properties with a high fluorescence quantum yield of 16% and large extinction coefficient of 52,000 M^–1^ cm^–1^. Probe **153** proved successful in imaging the endogenous production of H_2_O_2_ in mice with LPS-induced inflammation. Similarly, based on the fluorogenic dye QCy7, the Shabat group also demonstrated a new modular approach for preparing prodrugs (control **154** and probe **155**) with NIR fluorescence properties.^236^ Probe **155** could again be triggered by endogenous H_2_O_2_, this time produced by tumor cells and releasing camptothecin in the process.

**Figure 77:**
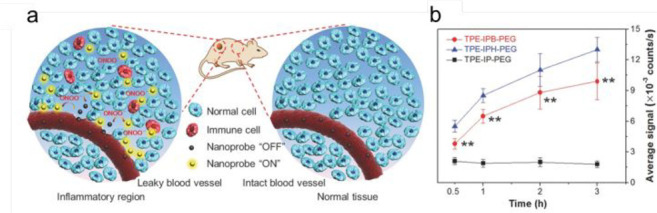
(a) Schematic illustration of probe **156** applied for specific *in vivo* inflammation imaging. (b) Time-dependent *in vivo* fluorescence images of inflammation-bearing mice before and after iv injection of probe **156**. The white circles indicate the MRSA-infected region. Reproduced with permission from ref (237). Copyright 2016 John Wiley & Sons.

In 2016, the Ding group designed a fluorescent imine functionalized tetraphenylethene (TPE)-derived nanoprobe **156** for the selective *in vivo* imaging of inflammation and visualization of the therapeutic effects of anti-inflammatory drugs (Figure 77).^237^ These TPE derivatives were encapsulated in lipid–PEG matrixes to form probe **156**, which showed no fluorescence in aqueous solution. On reaction with ONOO^–^, the phenylboronic ester undergoes oxidation to the phenol to produce an intramolecular hydrogen-bond with bright-yellow fluorescence. *In vivo* fluorescence imaging showed that probe **156** accumulated at inflammatory sites in mice, with a fluorescence “turn-on” response observed at high levels of ONOO^–^. The high specificity of probe **156** for ONOO^–^ enables precise and noninvasive monitoring of anti-inflammatory drug treatment effects.

**Figure 78:**
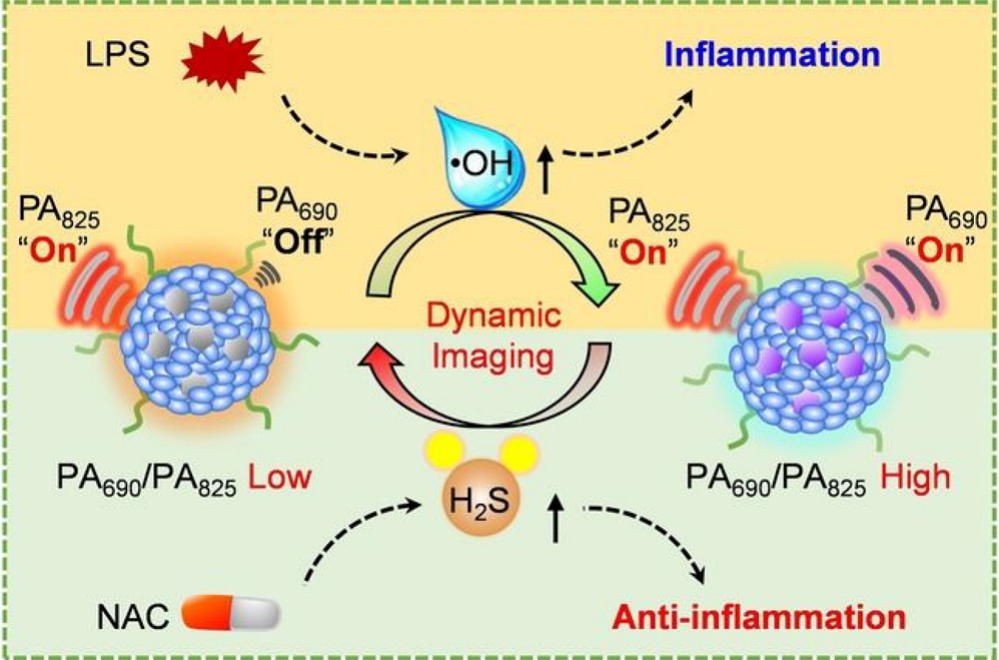
Design and functional principles of nanoprobe **157** for reversible ratiometric photoacoustic imaging of the •OH/H_2_S the redox cycle. Reproduced with permission from ref (238). Copyright 2022 John Wiley & Sons.

Reversible imaging probes enable dynamic visualization of the redox cycle between •OH and H_2_S, which is critical for studying the pathological processes involved in redox imbalances *in vivo*. Investigating oxidative stress, in 2022 Ye’s group reported a reversible ratiometric photoacoustic imaging nanoprobe 1-PAIN (probe **157**) for the imaging of the •OH/H_2_S redox in real time (Figure 78).^238^ This work builds on their previous research into electron-rich π-extended electrochemical materials to create probe EM-1 that is reversibly oxidized to its radical form by hydroxyl radical, and then reduced back to the π-conjugated probe by H_2_S. By encapsulating EM1 into a ROS/H_2_S-inert NIR semiconducting PCPDTBT polymer the desired PA imaging probe was obtained. Using the PA_690_/PA_825_ of probe **157**, the production of •OH during lipopolysaccharide-induced inflammation could be monitored, as it caused a 5-fold increase in ratio, reversibly switching back on when exposed to a high H_2_S-environment (e.g., by H_2_S induction with *N*-acetyl cysteine). Probe **157** may serve as a new and efficient tool to reversibly detect redox states in living organisms, thereby further advancing the study of diseases related to redox imbalances.

**Figure 79:**
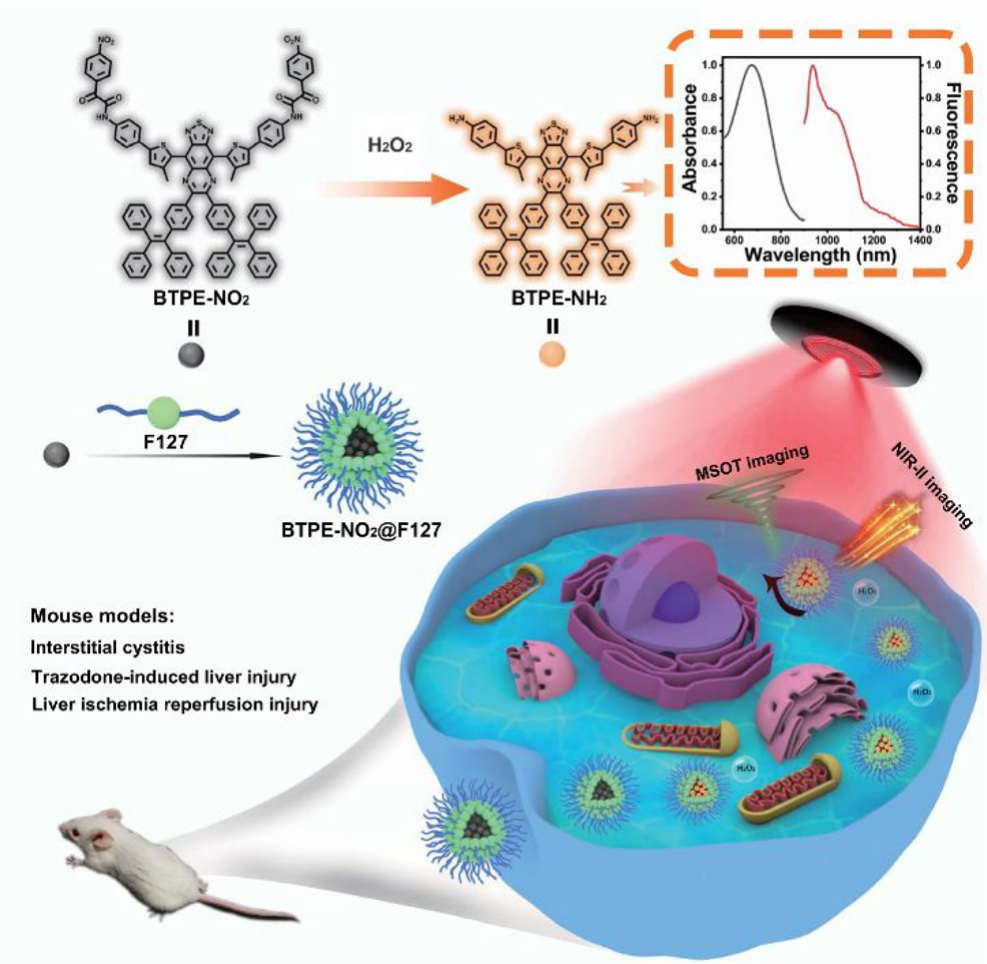
Preparation and use of nanoprobe **158** and its application for sensing H_2_O_2_ in interstitial cystitis. Reproduced with permission from ref (239). Copyright 2021 Springer Nature.

Interstitial cystitis is an inflammatory bladder disease characterized by pelvic pain and frequent and urgent urination. It is difficult to diagnose, as it does not currently have a specific clinical test, meaning that it is typically detected by exclusion. As with other inflammatory conditions, ROS levels are increased, and so H_2_O_2_ can be used for imaging of interstitial cystitis. This was demonstrated by the Zhao group in 2021, who created BTPE-NO_2_@F127 (probe **158**) by linking a benzothiazole core to two tetraphenyl hydrophobic molecular rotors and a nitrophenyl-oxoacetamide unit at either end as the recognition groups and fluorescence quenching agents (Figure 79).^239^ An amphiphilic polymer pluronic F127 was used to encapsulate BTPE-NO_2_ to complete this nanoprobe. The nitrophenyl-oxoacetamides were cleaved on exposure to H_2_O_2_ in interstitial cystitis and thereby activated the NIR-II fluorescence of the probe in the 950–1200 nm range. This also produces an ultrasonic signal that allows for multimode imaging of the disease. This nanoprobe should serve as a powerful tool for NIR-II fluorescence and multispectral photoacoustic tomography imaging of inflammatory interstitial cystitis, providing a potential new avenue for the diagnosis, study, and treatment of this condition.

**Figure 80:**
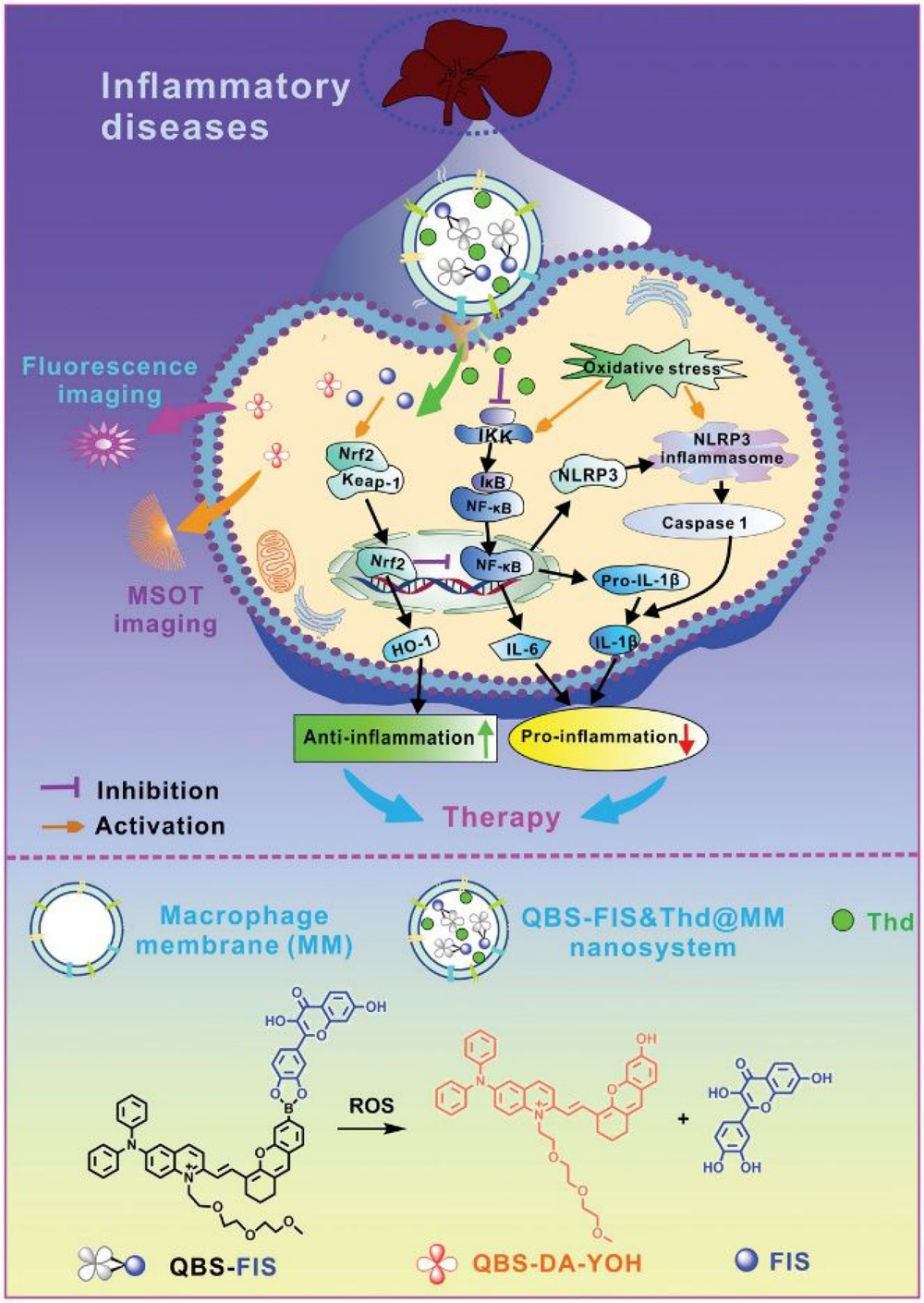
Schematic illustration of multifunctional probe **159** for diagnosis and therapy of acute liver inflammatory diseases. Reproduced with permission from ref (240). Copyright 2021 John Wiley & Sons.

In 2021, Wu developed a multifunctional nanosystem for targeting, imaging, and treating inflammatory diseases on demand by modulating inflammatory pathways (Figure 80).^240^ This system is based around a chromophore-drug QBS-FIS dyad, containing a NIR fluorophore linked to the Nrf2 activator fisetine (FIS) through a ROS-cleavable boronate ester, which also acts as the fluorescence quencher. Probe **159** was coated with macrophage membrane (*vide supra*) and coencapsulated with the drug thalidomide to improve therapeutic activity, producing the final probe QBS-FIS&Thd@MM. After intravenous injection, probe **159** was seen to migrate to the site of inflammation, where H_2_O_2_ would cleave the boronate ester to activate the fluorophore, emitting strong fluorescence signals at 715 nm, with a LOD 0.27 μM. FIS and thalidomide are both released *in situ* following ROS activation. This nanosystem can be used for liver/kidney inflammatory disease imaging, diagnosis, and recovery evaluation via fluorescence and photoacoustic imaging as well as being able to treat acute liver inflammation by release of active drugs.

**Figure 81:**
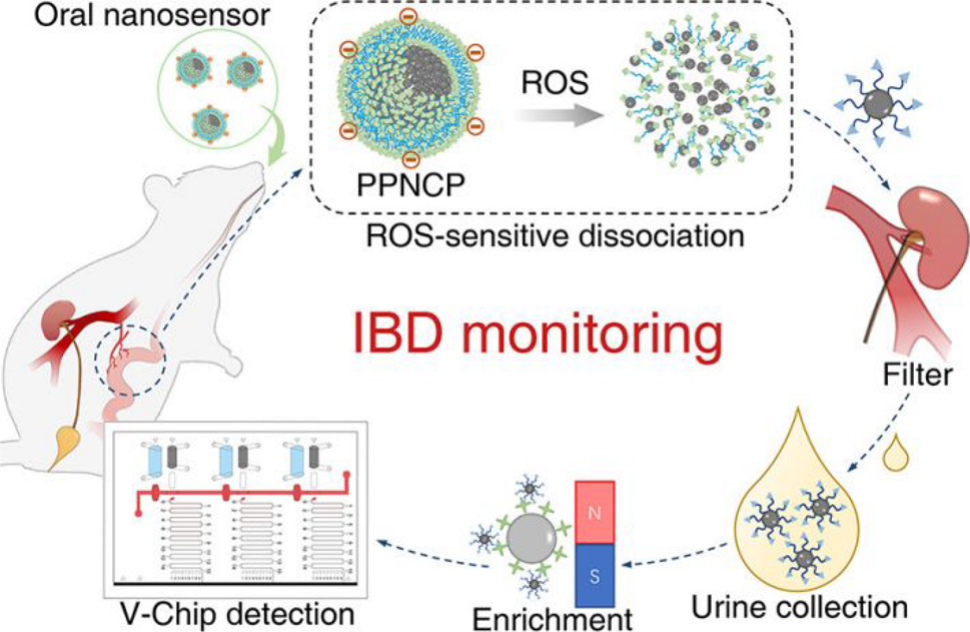
Orally administered nanosensor probe **160** dissociates into ultrasmall platinum nanoclusters in IBD-related inflammatory microenvironments for renal clearance and noninvasive urinary readout. Reproduced with permission from ref (241). Copyright 2022 American Chemical Society.

Inflammatory bowel disease (IBD) is a chronic autoimmune condition of increasing worldwide prevalence. In 2022, Song et al. designed an oral platinum nanomarker with a scalable urinary readout platform for noninvasive monitoring of IBD (Figure 81).^241^ Catalytic platinum nanoclusters that can be readily cleared by the kidney are encapsulated in ROS-responsive poly(1,4-phenylacetone dimethylene thione) (PPADT) to form supernanoparticles. This is further modified by addition of poly(styrenesulfonate) (PSS) to form PPNC@PSS (probe **160**) designed to target positively charged proteins overexpressed at inflammatory intestinal mucosa. Probe **160** can react with overabundant ROS, destroying the polymer coating to release the platinum nanoclusters. These will be cleared through the kidneys into urine, where Pt levels can be readily monitored. This probe showed significant signal differences between IBD model mice and healthy mice, proving a potentially useful new dual therapeutic and diagnostic tool. Similar approaches can be readily envisaged for targeted imaging of other inflammatory or oxidative-stress-based conditions by varying the structure of the polymer modifier.

**Figure 82:**
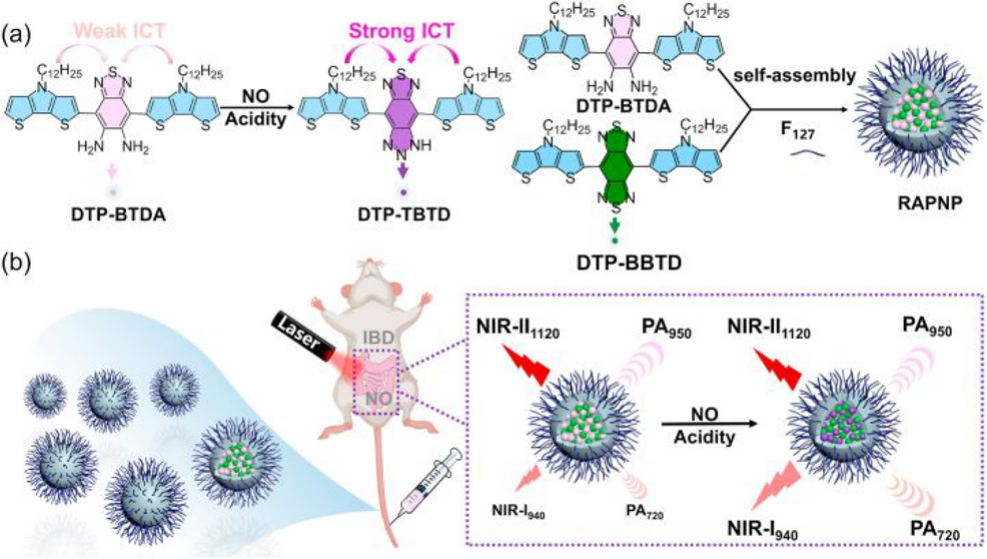
(a) Preparation and assembly of probe **161**, and its response to NO and acidity with DTP-BBTD as an internal reference. (b) Activation of the fluorescence signal at 940 nm and the PA signal at 720 nm of probe **161** in IBD mice by endogenous NO. Reproduced with permission from ref (242). Copyright 2023 Elsevier BV.

In 2023, the Wu group designed a NIRF/PA nanoprobe for the ratiometric imaging of NO *in vivo* (Figure 82).^242^ Ratiometric imaging was achieved by the use of two fluorophores combined into the single probe **161** RAPNP, with a nonresponsive DTP-BBTD serving as the reference, and a NO/acidity-responsive DTP-BTDA. Both are based on a highly electron donating dithiophenepyrrole (DTP) motif, which boasts strong ICT properties and a long emission wavelength. The BTDA portion of DTP-BTDA can be rapidly oxidized by NO, in weakly acidic environments, achieving activation of both the NIR and PA signals. Using a combination of both probes (in F127 polymer-encapsulated micelles) as a contrast agent, the ratiometric imaging of endogenous NO in inflammatory bowel disease was carried out by NIRF/PA modes.

**Figure 83:**
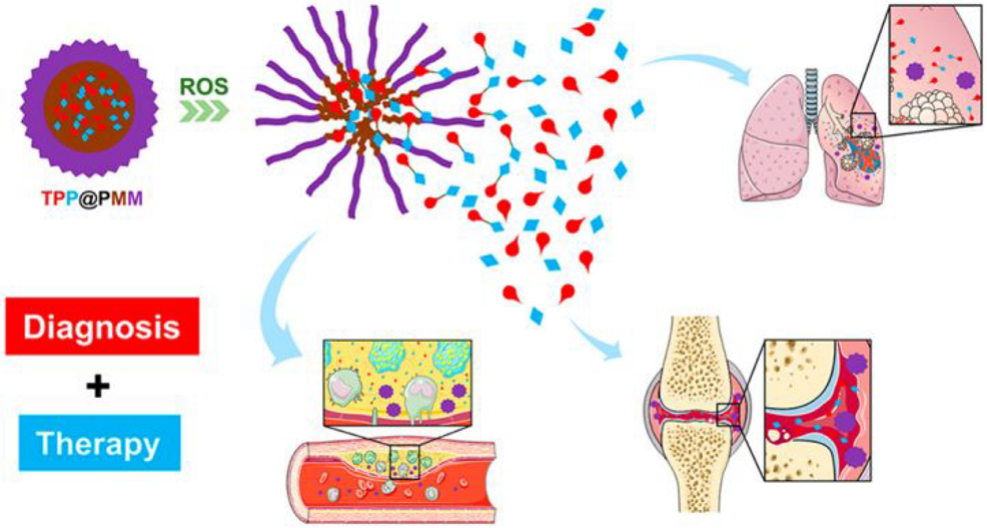
Prednisolone (Pred) is bridged to a two-photon fluorophore (TP) developed using a ROS sensitive bond to form a diagnosis-therapeutic compound TPP, which is then loaded by the amphipathic polymer PMPC–PMEMA (PMM) through self-assembling into the core–shell structured micelles (probe **162**). With a particle size of 57.5 nm, probe **162** can realize the accumulation in the inflammatory site via the oedematous tissue and the accurate release of anti-inflammatory drug Pred through the serial response to the local overexpressed ROS. Reproduced with permission from ref (243). Copyright 2020 American Chemical Society.

In 2020, the Wang group constructed a therapeutic nanoplatform TPP@PMM (probe **162**) with serial ROS-responsiveness for dimensional diagnosis and accurate treatment of inflammation (Figure 83).^243^ Prednisolone is bound to a TP fluorophore via a ROS-sensitive bond, forming the diagnostic/therapeutic compound TPP, which is then self-assembled with and amphiphilic polymer PMPC–PMEMA (PMM) to form probe **162** with a core–shell structure that can accumulate at inflammatory sites by penetrating oedematous tissue. Upon exposure to oxidative stress, multiple ROS responses occur. First, the ROS triggers decomposition of the micellar structure, turning the PMEMA polymer from hydrophobic to hydrophilic. This allows the TPP to be released into the inflamed system. Next, TPP reacts with ROS through cleavage of its ROS-sensitive bond, resulting in delivery of both prednisolone and the fluorophore directly at the site of inflammation, delivering effective therapeutic intervention, and allowing for high-resolution inflammation diagnosis using TP imaging.

**Figure 84:**
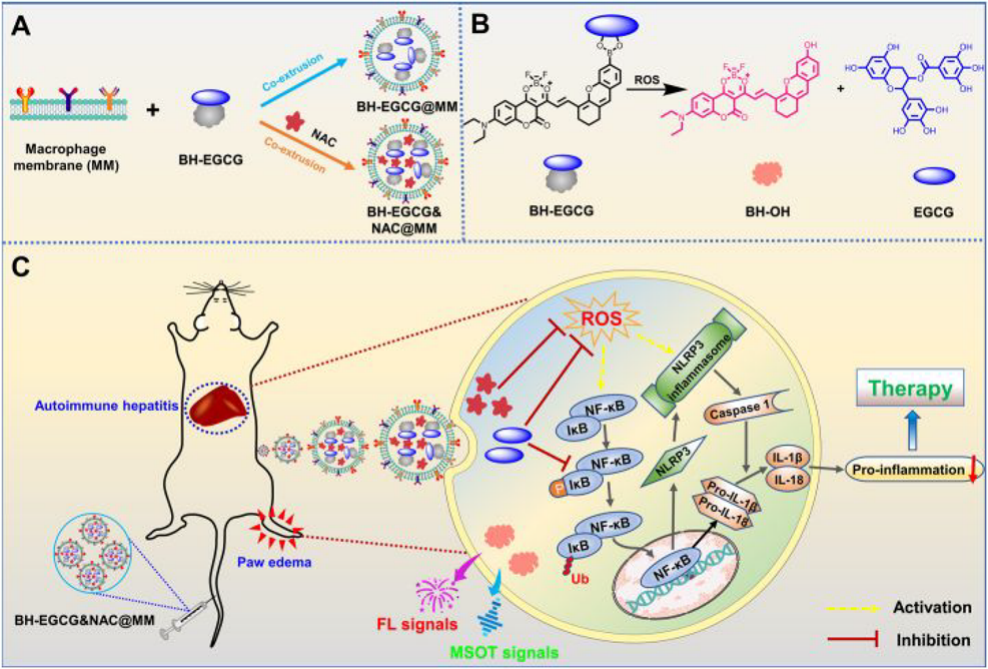
(A) Preparation of BH-EGCG@MM and BH-EGCG&NAC@MM. (B) Response mechanism of BH-EGCG toward ROS. (C) Multifunctional activity of BH-EGCG&NAC@MM, including diagnosis of ConA-induced autoimmune hepatitis and CAR-induced hind paw edema via NIR fluorescent imaging and optoacoustic imaging, and efficient therapy of ConA-induced acute autoimmune hepatitis and CAR-induced hind paw edema via inhibiting NF-κB pathway and suppressing NLRP3 inflammasome formation. Reproduced with permission from ref (244). Copyright 2022 Elsevier BV.

In 2022, Wu et al. proposed an activatable targeted nanosystem BH-EGCG&NAC@MM (probe **163**) for detecting and imaging lesions in immune-mediated inflammatory diseases, concurrently providing treatment and inhibition (Figure 84).^244^ In probe **163**, a NIR chromophore and NF-κB/NLRP3 inhibitor epigallocatechin-3-gallate were combined via a boronic acid linker, which, as seen repeatedly above, is ROS-cleavable. BH-EGCG units formed stable nanoparticles in aqueous solution, which were then encapsulated in macrophage cell membrane, a common technique seen previously in this review. Furthermore, *N*-acetylcysteine, a potent antioxidant could coencapsulate to further enhance the probe’s therapeutic ability to produce the final nanosystem BH-EGCG&NAC@MM. Guided by the macrophage membrane, this nanosystem can travel to sites of inflammation, where the boronate ester linker can be oxidatively cleaved, releasing the fluorophore and both drugs. This enables both imaging and therapeutic treatment, as confirmed in mouse models of autoimmune hepatitis and hind limb edema.

**Figure 85:**
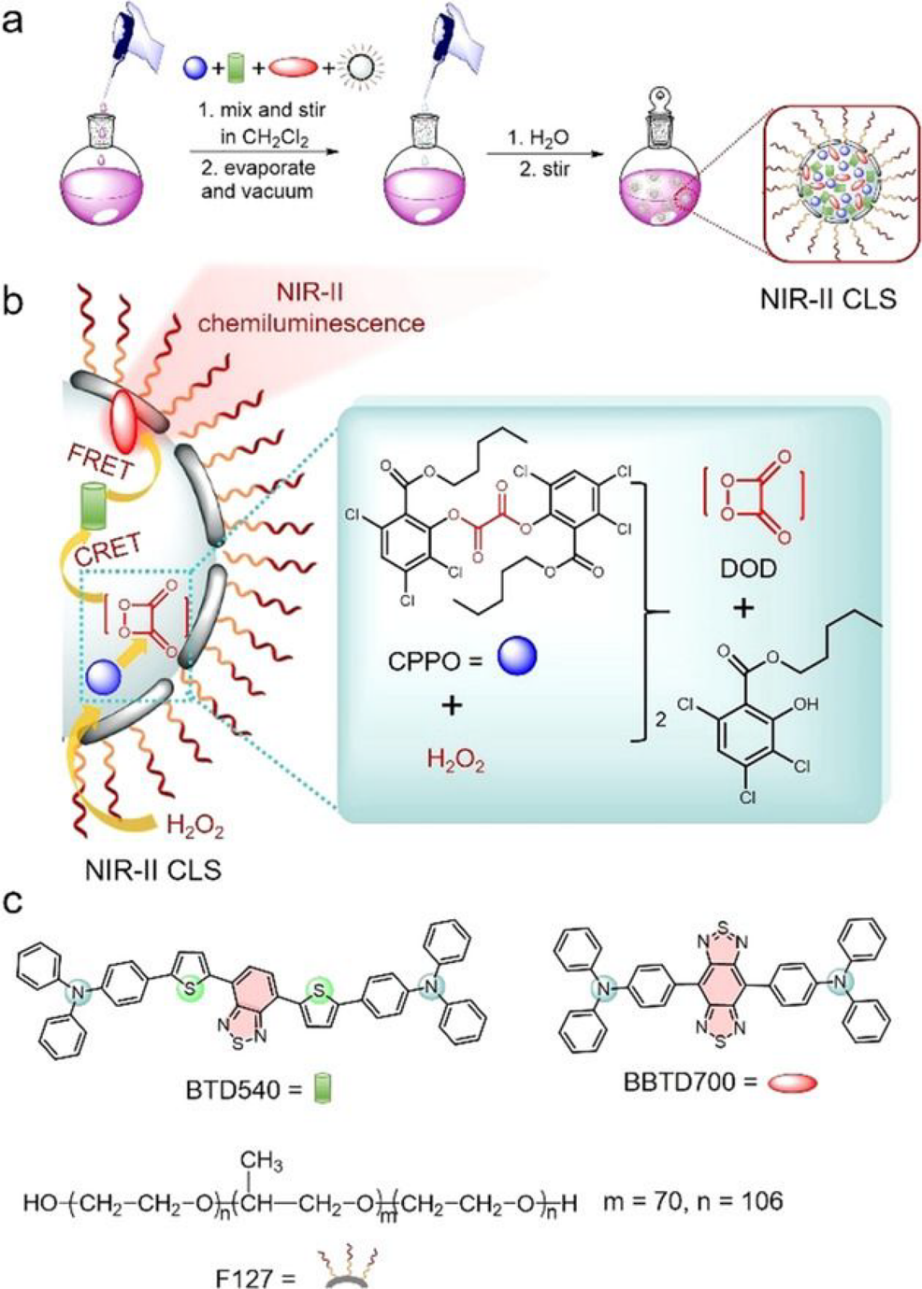
(a) Preparation of probe **164**. (b) Working principle of probe **164** for generating emission in the presence of H_2_O_2_. (c) Structure of fluorophores BTD540, BBTD700, and F127. Reproduced with permission from ref (245). Copyright 2020 John Wiley & Sons.

A new chemiluminescent NIR-II probe **164** was proposed by Zhang et al. in 2020 for imaging inflammation with deep tissue penetration (approximately 8 mm) (Figure 85).^245^ Probe **164** takes the form of a multicomponent assembly, wherein bis-[3,4,6-trichloro-2-(pentyl-oxycarbonyl)phenyl]oxalate is first oxidized by H_2_O_2_ to produce an unstable 1,2-dioxetanedione (DOD) intermediate. DOD can then transfer its chemical energy to fluorophore BTD540 via chemiluminescent resonance energy transfer (CRET), and this is then passed on to fluorophore BBTD700 through FRET, with this latest compound producing NIR-II fluorescence emission. Probe **164** was successfully used to detect local inflammation in mice, with an acceptable signal-to-noise ratio of 4.5.

**Figure 86:**
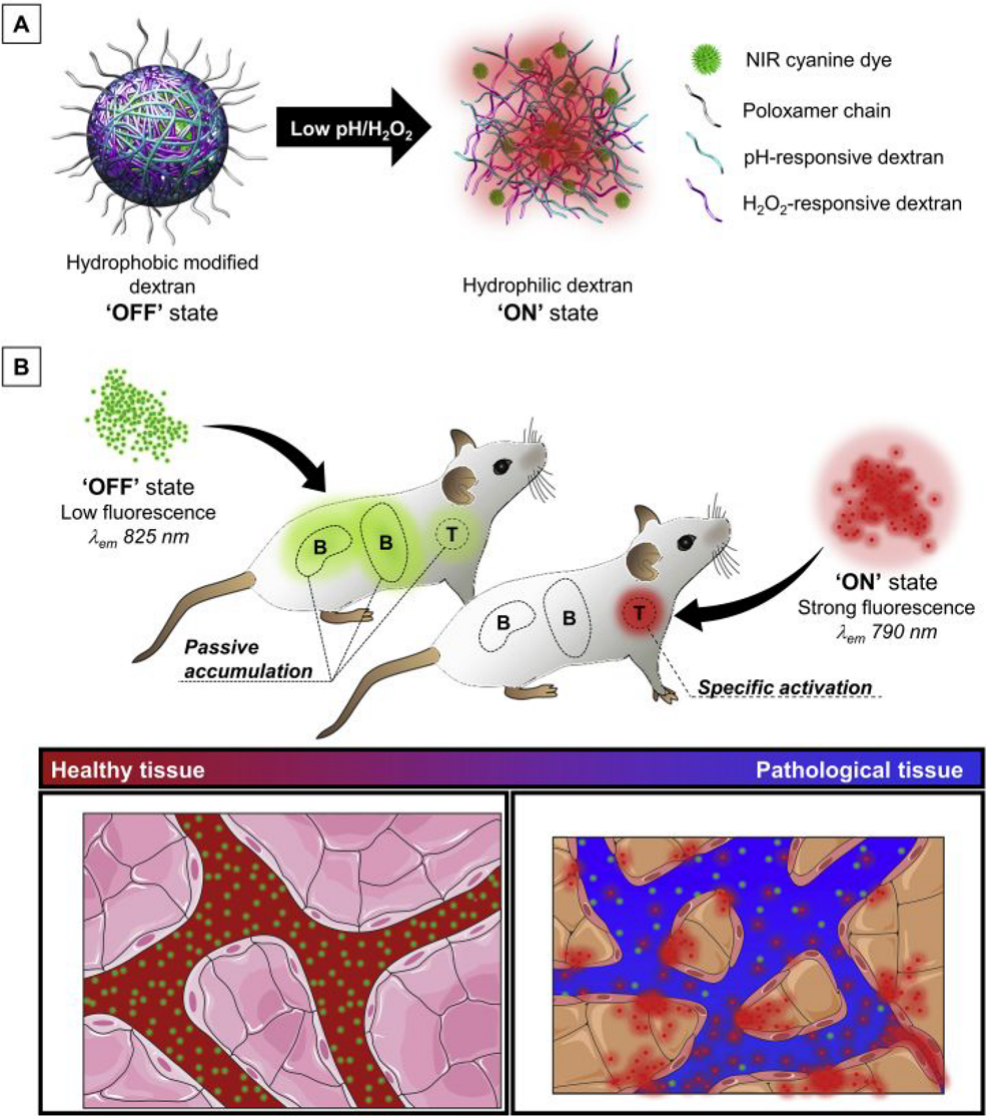
(A) pH- and H_2_O_2_-responsive polymeric materials control the fluorescent intensity and spectral profile IR-780 dye molecules. Particles disassemble under acidic and/or oxidative stress conditions as the modified polymers return to their native water-soluble state, which releases large amounts of dyes, relieves self-quenching, and triggers fluorescence activation. (B) The nanoprobes remain “off” in blood and healthy tissue and are turned “on” by extracellular acidosis and oxidative stress in damaged/pathogenic tissue. Reproduced with permission from ref (246). Copyright 2017 Elsevier BV.

In 2017, Almutairi et al. reported a combination of stimuli-responsive polymers and NIR fluorescent probe **165** as a powerful tool for the concurrent detection of acidosis and oxidative stress associated with an inflammatory microenvironment (Figure 86).^246^ In their report, dextran-based materials were employed to encapsulate NIR IR-780 dyes, with the enforced close proximity leading to fluorescence quenching, as well as spectral modulation to produce a weak emission signal centered around 825 nm. The dextran-based nanoparticles used were designed to respond to both acidic pH and H_2_O_2_ by using different forms of dextran in the same probe: one H_2_O_2_-cleavable and the other sensitive to acidity. Therefore, on exposure to inflammation, the nanoparticles were activated, the polymers dissolved, and the fluorophores released. This causes both a significant fluorescence emission enhancement as well as a shift in the emission at 790 nm. These nanoprobes **165** were applied to detect pH and H_2_O_2_ changes in inflammatory tumor tissues and can be used for *in vivo* detection of arthritic joints and inflamed paws of mice.

Selenocysteine (Sec) is one of the primary sources of reactive selenium in cells, with a known antioxidant role in a range of liver diseases. Due to its high reactivity and instability, Sec content is difficult to determine in living cells as well as *in vivo*. Chen and Yu began to address this issue in 2017 by developing NIR probe **166** for qualitative and quantitative determination of Sec in living cells and *in vivo*.^247^ The design of probe **166** is based around a heptamethine cyanine NIR fluorophore, bis(2-hydroxyethyl) disulfide as the Sec-reactive motif, and d-galactose as the liver-targeting unit. In the presence of Sec, the disulfide bond of the probe is reduced, leading to rapid intramolecular cleavage of the carbamate motif. This unmasks a *N*-methyl group capable of strong electron donation, causing a significant spectral blue-shift due to the ICT nature of the system. As the concentration of added Sec is gradually increased, the maximum absorption intensity of probe **166** gradually decreased at 782 nm, with a new enhanced maximum absorption peak appearing at 610 nm. Correspondingly, the maximum fluorescence wavelength shifts from 800 to 750 nm. The ratiometric fluorescence intensity log(*F*_750_/*F*_800_) exhibits good linearity, with Sec concentrations up to 20 μM (*F*_750_/*F*_800_), with a LOD of 90 mM. Probe **166** was shown to have excellent specificity and biocompatibility, and selectively accumulated in the liver. Hence, this probe was successfully employed to target the liver and detect Sec concentrations in normal and acute hepatitis BALB/c mice models.

**Figure 87:**
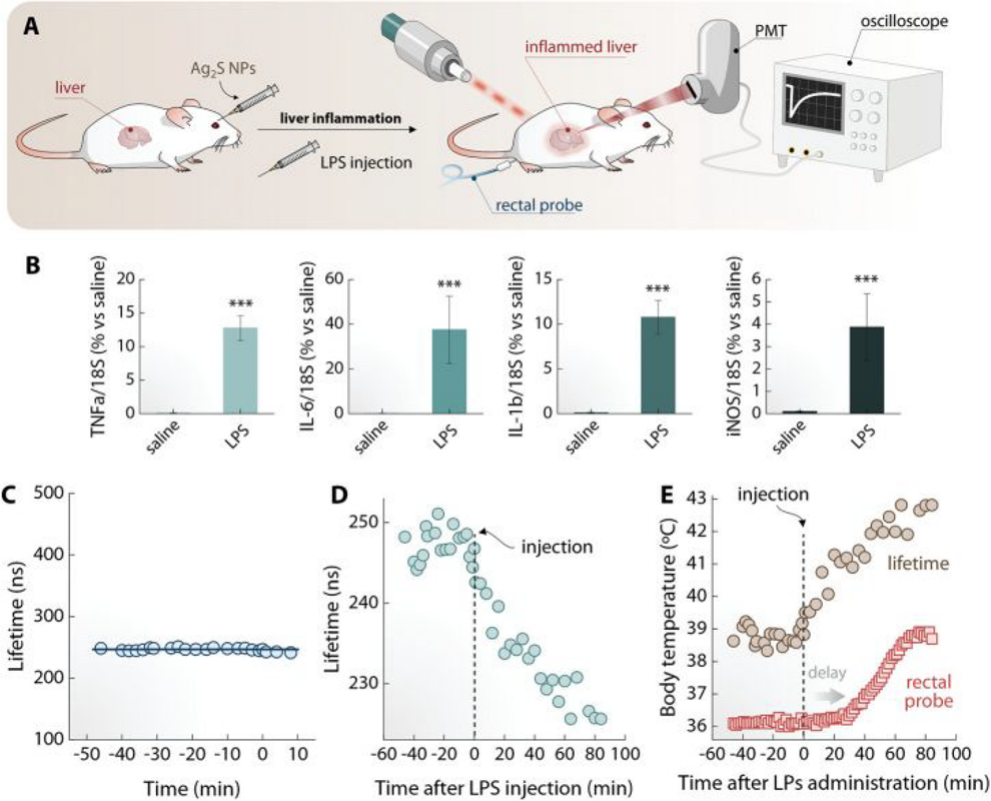
(A) Schematic representation of the experimental procedure for monitoring liver temperature during LPS-induced inflammation using Ag_2_S nanoparticles (**167**). (B) Gene expression of proinflammatory markers (TNF-a, IL-6, IL-1b, and iNOS) in hepatic tissue after LPS injection (*** *Pv* < *v*0.001 vs saline). (C) Time evolution of the fluorescence lifetime before LPS injection. (D) Time evolution of the fluorescence lifetime after LPS injection. (E) Time evolution of liver temperature after LPS injection (brown circles) and rectal temperature (red squares). Reproduced with permission from ref (248). Copyright 2022 John Wiley & Sons.

Temperature is key to the normal function of organs and is known to increase when tissues enter an inflammatory state following trauma or infection. In 2022, Benayas and co-workers developed luminous Ag_2_S nanoparticles with a temperature- dependent fluorescence lifetime for measuring absolute liver temperature in mice with lipopolysaccharide-induced inflammation (Figure 87).^248^ In order to allow for operation in aqueous media, the surface of these Ag_2_S nanoparticles was coated with PEG, producing pegylated Ag_2_S nanoprobes emitting a strong NIR-II signal at 1250 nm under 800 nm excitation. These PEG-Ag_2_S probes **167** were successfully used to monitor liver temperature in a mouse model of inflamed liver, with a remarkable thermal sensitivity (3% °C^–1^) and thermal resolution (<0.3 °C).

Clarifying the intrinsic relationship between diseases and mitochondrial viscosity is of great importance for the diagnosis and treatment of fatty liver, inflammation, and cancer, with elements of this already highlighted in previous sections already. However, the development of a single mitochondrial viscosity fluorescent probe capable of visualizing multiple disease models and achieving photodynamic treatment is still a significant challenge.^249^ In 2022, Dong and co-workers began to address this challenge, designing the mitochondria targeting and viscosity sensitive NIR probe **168**.^250^ This probe was composed of a substituted oxanthracene as the electron donor and a pyridinium cation unit as the acceptor and mitochondria-targeting group. Once within the mitochondria, an increase in viscosity causes probe **168** to exhibit a significant “turn-on” response at 720 nm, with a good linear relationship with mitochondrial viscosity (0.89 cP–945 cP). Using probe **168**, visualization studies were conducted on mitochondrial viscosity in tissue samples from fatty liver tissue, inflammatory live mice, live tumor mice, and clinical cancer patients. Additionally, probe **168** was applicable for use in photodynamic cancer therapy, with irradiation at 635 nm leading to the production of ROS with direct therapeutic effect, showing a 25% decrease in tumor size after 21 days of administration. Thus, probe **168** is capable not only of imaging mitochondrial viscosity in a variety of inflammatory diseases but also effectively delivering targeted photodynamic treatment, which is a promising theranostic combination for such systems.

In 2022, Yuan and co-workers designed a multicolor fluorescent probe **169** for monitoring cell viscosity, polarity, and leucine aminopeptidase (LAP) in inflammation-mediated cancer progression.^251^ The probe was formed by the condensation of a 4-methylquinolinium salt and *N*,*N*-dimethylaminobenzaldehyde, resulting in the formation of a typical *D*–π–*A* system. The positive charge in the structure of probe **169** enables the probe to target mitochondria, and *N*,*N*-dimethylaminobenzaldehyde enables viscosity recognition as it can undergo intramolecular rotation to produce twisted intramolecular charge transfer, thereby quenching the fluorescence within low-viscosity environments. The leucine element is the specific recognition site for LAP, which on cleavage removes the cationic component of the probe, thus weakening ICT and blue shifting the fluorescence emission. Probe **169** has a good linear response to viscosity, and the emission is unaffected by temperature and can detect LAP up to 50 U/L. In the 0–10 U/L range, the fluorescence intensity is linear against LAP, with a reported LOD of 0.08691 U/L. As the polarity of the system increases, the fluorescence emission wavelength is gradually red-shifted from 489 to 612 nm. Probe **169** was successfully used to reveal changes in mitochondrial viscosity in inflamed cells, changes in LAP in cancer cells, and decreases in mitochondrial polarity in cells during epithelial–mesenchymal transformation. Additionally, probe **169** has been successfully used to assess changes in the microenvironment during inflammatory responses in zebrafish.

To explore the physiological role of pyroglutamate aminopeptidase 1 (PGP-1) in inflammation, Wu et al. designed NIR fluorescence probe **170** in 2018, boasting high selectivity and ultrahigh sensitivity for the detection of PGP-1.^252^ A hemicyanine fluorophore was selected for its long excitation and emission wavelength with good biocompatibility. In the presence of PGP-1, the peptide bond of pyroglutamic acid linked to the fluorophore is severed, leading a fluoroescence enhancement with a good linear response to PGP-1 in the concentration range of 0.01–0.25 μg mL^–1^, and a LOD of 0.18 ng mL^–1^. Using probe **170**, the upregulation of PGP-1 in the legs and liver of BALB/c mice was monitored under stimulation by lipopolysaccharides/d-galactosamine. This work suggests that PGP-1 is directly involved in inflammatory responses in the body and may be a previously overlooked inflammatory cytokine.

**Figure 88:**
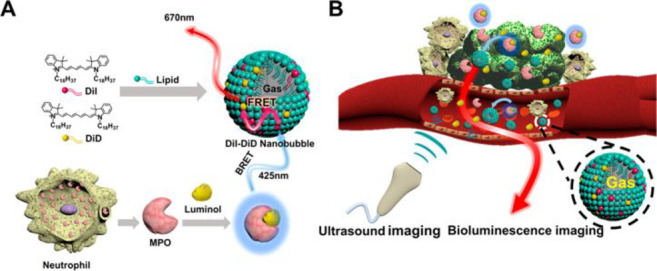
Schematic illustration of luminol + probe **171** for dual-modal imaging of MPO activity. Reproduced with permission from ref (253). Copyright 2019 American Chemical Society.

Myeloperoxidase (MPO) is a key component of the inflammatory process, as it is produced by neutrophils and monocytes as part of the innate immune system’s defense mechanisms. Detection and monitoring of MPO can therefore serve as a good biomarker of inflammation. Until recently, this was done using luminol bioluminescent assays. However, the blue light (λ_max_ = 425 nm) it emits is ultimately ineffective in tissue-based or *in vivo* assays due to poor penetration. Therefore, in 2019, the Dai group prepared doped fluorescent dye nanobubbles (DiI–DiD NBs, probe **171**) as energy transfer relay agents to improve the emission wavelength (Figure 88).^253^ These Dil–DiD NBs are composed of lipophilic dyes capable of red-shifting the luminol-emitted blue light into the NIR. Energy transfer and wavelength red-shifting are achieved by combined bioluminescence resonance energy transfer and fluorescence resonance energy transfer (BRET–FRET) within a single probe, thanks to the large overlap of spectra between the two fluorophores. In mice with lipopolysaccharide-induced inflammation and triple-negative breast cancer, probe **171** emitted a bright fluorescence signal at lesion sites after intravenous injection. By combining the BRET–FRET fluorescence system with the advantages of ultrasound imaging, this system may be able to address a critical need for the use/need of multimodal inflammation imaging probes in medical diagnostics.

**Figure 89:**
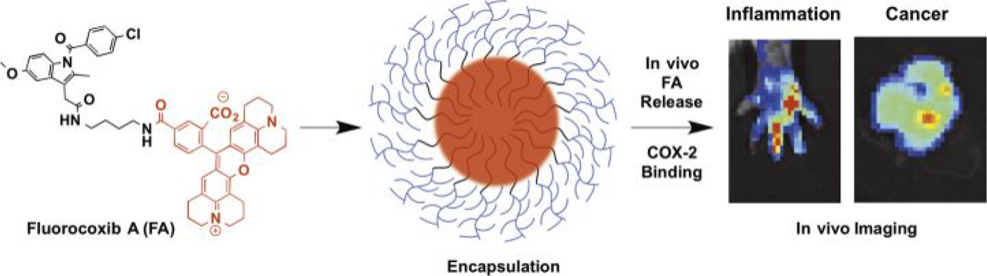
ROS-responsive micellar nanoparticle which solubilize FcA for COX-2 visualization *in vivo*. Reproduced with permission from ref (254). Copyright 2016 Elsevier BV.

COX-2, a common cancer biomarker (see section 3), is typically overexpressed in inflammation and so is a potentially powerful biomarker for early clinical detection of both cancer and inflammatory diseases. In 2016, the Duvall group developed a ROS-responsive micellar nanoparticle FcA-NPs (probe **172**), which can solubilize fluorescent COX-2 selective inhibitor fluocoxib A (FcA), enabling COX-2 visualization *in vivo* in inflammation (Figure 89).^254^ As with the PMEMA polymer discussed previously, exposure to ROS renders the hydrophobic material hydrophilic (although oxidation from sulfide to sulfoxide and sulfone), releasing FcA at the site of inflammation where it is free to bind to COX-2. After intravenous administration of probe **172** in wild-type mice, its pharmacokinetic and biological distribution profiles were evaluated, and optimal imaging fluorescence was observed after 4–8 h. Probe **172** successfully imaged the inflammation induced by carrageenan in rat and mouse foot pads and 1483 HNSCC tumor xenografts, with a 10-fold increase in fluorescence over normal tissue. Pretreatment with COX-2 inhibitor indomethacin blocked the targeted binding of FcA, which confirmed the specific binding of COX-2 and the local retention of FcA at the site of release. Probe **172** represents the first formulation of FcA for intravenous administration with good signal-to-noise ratio in inflammatory, precancerous and malignant tissues and could help enable the clinical adoption of low water-solubility FA.

As illustrated throughout this review, inflammation is not only a standalone disease state but plays a key role in other conditions such as cancer, organ damage, and Alzheimer’s disease. In 2022, Kwon et al. developed the universal Cas-1 probe **173** for monitoring the activity of caspase-1 in a variety chronic inflammatory disorders.^255^ Probe **173** was synthesized by linking fluorophore Cy5.5 and quencher BHQ-3 using caspase-1 substrate (G-W-E-H-D-G-K) as the linker. In the presence of caspase-1, the quencher was removed, and the fluorescence of Cy5.5 was restored. Good biocompatibility led to good cell penetration, and so it could be used to effectively image caspase-1 in a variety of inflammatory mouse models, including endotoxic shock, inflammatory bowel disease, transplanted cancer, and Alzheimer’s disease. The authors noted that their probe could detect neuroinflammation two months prior to the onset of any cognitive impairment in AD models. The ability of this probe to image caspase-1 activity efficiently and with good spatiotemporal resolution in such a range of inflammatory diseases is very promising and could lead to its adoption in a variety of research and/or clinical settings.

**Figure 90:**
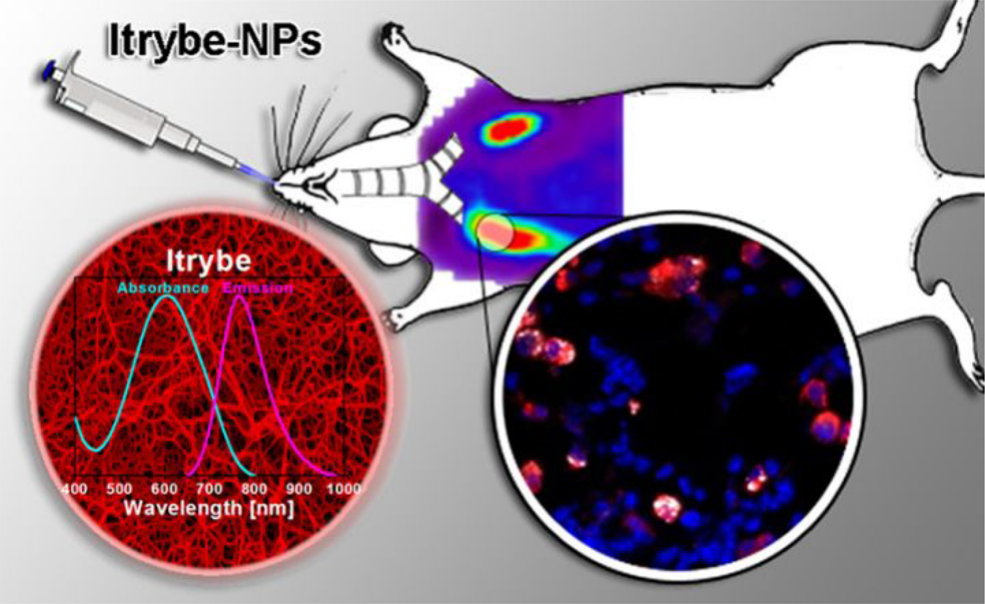
*In vivo* and *ex vivo* fluorescence reflectance imaging of an ovalbumin-based allergic airway inflammation using intranasal application of probe **174** for imaging alveolar M2 macrophages. Reproduced with permission from ref (256). Copyright 2015 American Chemical Society.

In 2015, Alves et al. designed inhalable polystyrene nanoparticles (Itrybe-NPs, probe **174**) loaded with near-infrared fluorescent dye Itrybe to image innate immune cells in mouse lungs (Figure 90).^256^ As the recruitment of the innate immune system is crucial in the inflammatory processes, this serves as a good tool for imaging asthma. After introducing probe **174** through the nose SKH-1 mice with ovalbumin allergic airway inflammation (AAI), *in vivo* and *in vitro* fluorescence reflection imaging showed that the fluorescence intensity of AAI lungs were significantly higher than that of the control group. *In vitro* immunofluorescence analysis of lung tissue indicated that the probe was mainly taken up by CD68^+^CD_11c_^+^ECF-L^+^MHCII_low_ cells in the peribronchial and alveolar regions, identifying them as M2 macrophages. Through confocal microscopy, overlapping section analysis, and flow cytometry, it was found that the number of macrophages containing Itrybe-NPs in the lungs of AAI mice was significantly greater than that of the control group. This imaging method and nanoprobe type can be used to monitor AAI over time and provides a new tool for the imaging of lung inflammation and to help study the role of macrophages and could be useful in determining the effectiveness and mechanism of inhalable nanotherapies.

**Figure 91:**
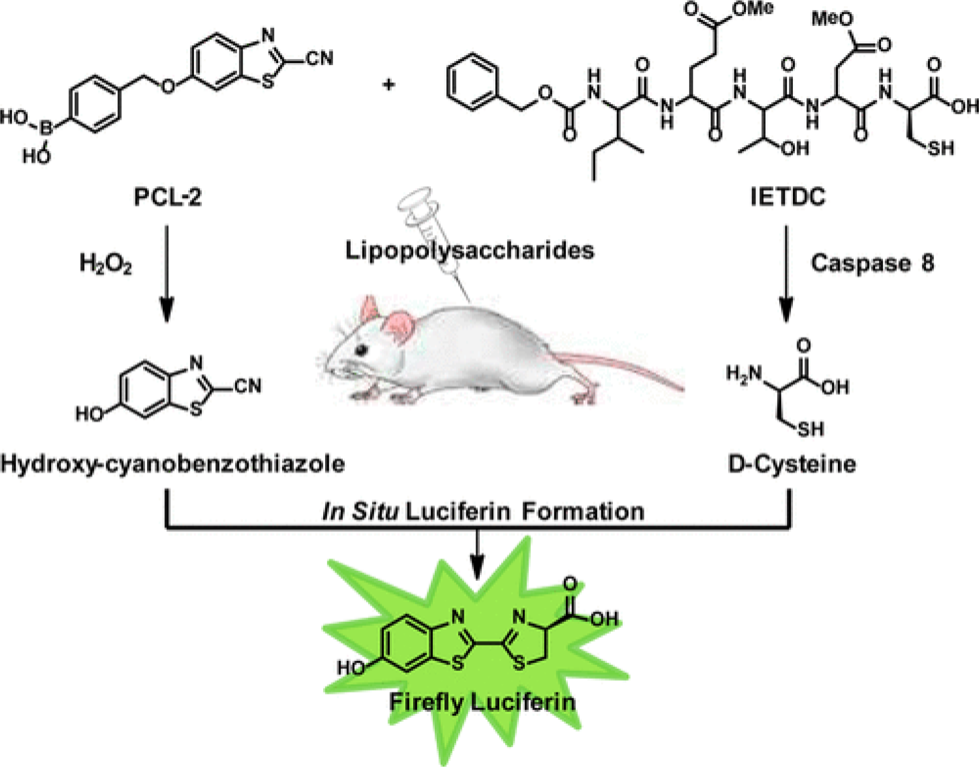
Design strategy behind probe **175** for simultaneous detection of H_2_O_2_ and caspase 8 activity through release of HCBT and d-cysteine and *in situ* formation of firefly luciferin. Reproduced with permission from ref (257). Copyright 2013 American Chemical Society.

In 2013, Bertozzi and Chang developed what they termed a general strategy for dual-analyte detection in living animals.^257^ Uniquely, this sensing platform does not require activation or localization of a probe-derived fluorescent species but instead relies on the *in situ* formation of firefly luciferin from two separately administered precursors (Figure 91). To achieve this, peroxy-caged luciferin-2 (PCL-2) was developed, capable of releasing 6-hydroxy-2-cyanobenzothiazole (HCBT) on reaction with H_2_O_2_. Alongside this, peptide-based species z-Ile-Glu-TrAsp-d-Cys (IETDC) was also administered, releasing d-cysteine in the presence of caspase-8. d-Cysteine and HCBT then combine *in situ*, producing fluorescent firefly luciferin, effectively producing a dual H_2_O_2_/caspase-8 fluorescent probe **175**. This approach to fluorescent probe design offers an incredibly powerful new perspective on the development of dual-analyte probes and a powerful approach for imaging oxidative stress and inflammatory processes *in vivo*.

**Figure 92:**
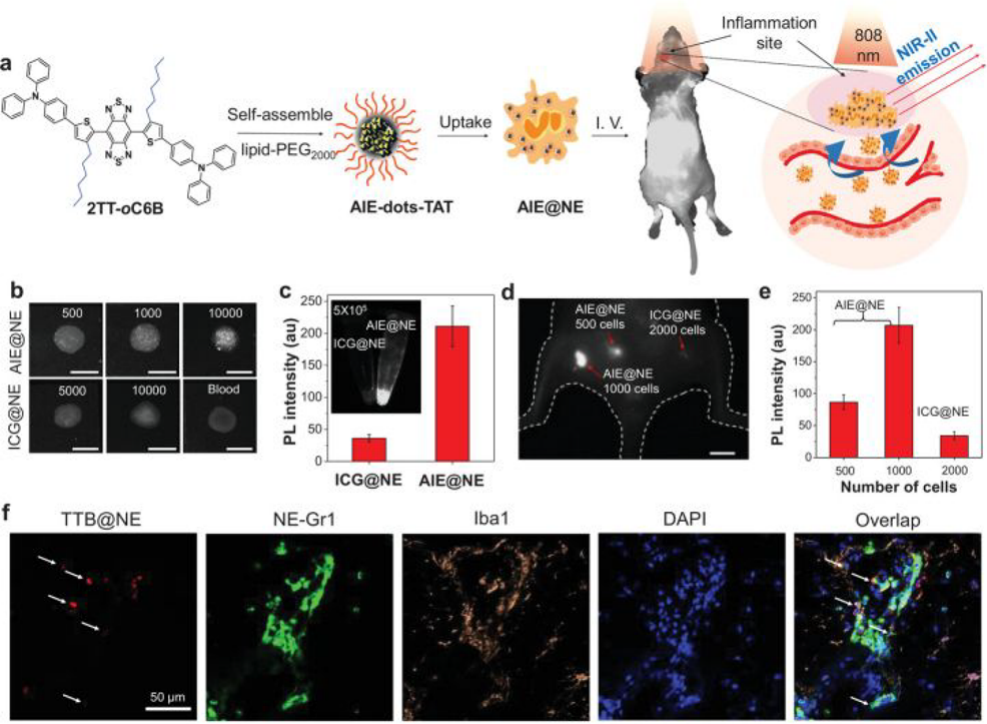
(a) Schematic of the NE-mediated NIR-II AIE dots for brain inflammation imaging. (b) NIR-II fluorescence images with different cell number (1000 nm LP, 50 ms). (c) Average fluorescence signals at cell number of 5 × 10^5^. (d) Subcutaneous fluorescence images with different cell number. (e) Average fluorescence signals of the data from (d). Reproduced with permission from ref (258). Copyright 2020 John Wiley & Sons.

The lack of high quantum yield organic NIR-II fluorophores is a bottleneck to the development of truly effective bioimaging fluorescent probes that operate within that range. With this in mind, in 2020, the Tang and Ding groups proposed a strategy for overcoming this issue by using simple structural isomerization of AIE-based fluorophores. Simply put, it uses existing AIE-capable systems and designs structural isomers to induce backbone distortion and rotor twisting (Figure 92, probe **176**).^258^ This was demonstrated with the development of probe 2TT-*o*C6B for brain inflammation imaging. This probe was derived from 2TT-*m*C6B by simple shifting the hexyl groups from the 4- to the 3- position of the thiophene rings, resulting in a change in wavelength and fluorescence intensity and an increase in quantum yield to 11%, with an impressive signal-to-noise of 30.6. By appending this probe to neutrophils, the authors successfully transported the probe **176** through the BBB for accumulation in inflamed parts of the brain for noninvasive identification of inflammation through intact scalp and skull, up to a depth of approximately 3 mm.

## 7 Conclusion and Outlook

Over the past decade, fluorescence imaging technologies for human disease diagnosis have progressed rapidly, with myriad small molecule sensors, chemodosimiters, nanoprobes, protein probes, polymeric assemblies, etc., now available to visualize and evaluate disease-related bioactive molecules. As illustrated throughout this review, different tools boast different unique advantages and downsides, and careful selection of a specific probe is needed for each targeted problem.

Small organic molecule-based probes have the advantage of simple structures, convenient operation, stable optical properties, and excellent biocompatibility, and have favorable clinical application prospects. Nanoprobes can be used to develop multimodal and multifunctional fluorescent imaging materials with the practical advantage of easy functionalization and derivatization. Protein-derived probes have the merit of preferable targeting and superior performance, and are more appropriate for visualization at high temporal and spatial resolution. It should be noted that many nanoparticle probes incorporate protein/peptide groups as their targeting units. Similarly, nucleic acid probes boast inherently high selectivity for DNA and RNA, and as such are best suited for such targeted applications.

In this review, we have provided an extensive overview of recently reported fluorescent probes for the characterization and study of different human diseases. The molecular mechanisms of specific recognition and fluorescence manifolds have been described, and the biological applications of these systems emphasized, with a particular focus on the detection of key disease biomarkers, cellular tracking *in vivo*, drug efficacy evaluation, and surgical navigation. Despite the significant progress over the last few decades, we believe the development of fluorescent probes for disease diagnosis is still in its infancy, with many more probes still being developed. However, many challenges remain, and it is our belief that chief among these are the following:

(1) Issues of metabolism and biotoxicity of probes *in vivo* need to be addressed in the case of molecular probes and nanoprobes containing heavy metal ions. Toxicology and pharmacokinetic studies should be routinely conducted when developing such systems to ensure biocompatibility and to maximize the potential for future clinical adoption. Sustained efforts should be made to reduce the size and number of nanomaterials required while making them biodegradable and rapidly metabolized and clearable.
(2) Continued improvement of fluorescence systems is needed, with a focus on the chemistry underpinning these systems. In particular, the development of novel specific recognition reactions for either existing or newly identified biomarkers of diseases is critical and necessary to continue to expand the scope of activatable fluorescent probes. In combination with new and improved fluorophores with high quantum yields, this will help improve the specificity and sensitivity of fluorescent probes for accurate and precise diagnosis.
(3) Additional work to improve the targeting of probes toward specific tissue types is essential for both improving targeting groups and moving beyond specific targeting motifs, with the view to generate a more inherent targeting strategy.
(4) Further progress on activatable fluorescent probes with emission wavelengths in the NIR-II region is expected to bring about significant improvement in spatial resolution, while resolving many of the issues associated with autofluorescence and tissue penetration depth.
(5) Combining fluorescence technologies with various other imaging techniques such as MRI, PA, PET, etc., some examples of which have been discussed throughout this review. This will enable exploitation of multimodal imaging technologies to achieve accurate tracking and labeling of disease-related bioactive molecules in living systems.
(6) Finally, the development of low-cost, high-efficiency, scalable, and sustainable production strategies that satisfy clinical needs and promote the commercialization and industrialization of fluorescent probes will be key to large-scale adoption of fluorescent probes in clinical settings.

In conclusion, while currently only a limited number of fluorescent probes have entered clinical environments,^259,260^ we expect that the development of novel fluorescent probes for diagnostic (and therapeutic) applications for a diverse range of human diseases will remain at the forefront of future fluorescence imaging research. The continuing improvements being made to fluorescent probes will soon enable rapid detection of critical disease biomarkers at deeper biological sites, at lower concentrations, and at an earlier stage in a disease’s development, thereby enabling the provision of more accurate clinical diagnosis alongside a more extensive understanding of the physiological roles of bioactive molecules in the targeted diseases.
